# Gold(I) Complexes with Bulky Phosphanes: A Dual Approach to Triplet Harvesting and Hydroamination Catalysis

**DOI:** 10.1021/acs.inorgchem.4c04964

**Published:** 2025-02-11

**Authors:** Araceli de Aquino, Nazaret Santamaría, Artur J. Moro, David Aguilà, Auxiliadora Prieto, M. Carmen Nicasio, João Carlos Lima, Laura Rodríguez

**Affiliations:** † Departament de Química Inorgànica i Orgànica, Secció de Química Inorgànica, Universitat de Barcelona, and Institut de Nanociència i Nanotecnologia (IN2UB), Martí i Franquès 1-11, 08028 Barcelona, Spain; ‡ Departamento de Química Inorgánica, 16778 Universidad de Sevilla, Calle Profesor García González 1, 41012 Seville, Spain; § LAQV-REQUIMTE, Departamento de Química, Faculdade de Ciências e Tecnologia, Universidade Nova de Lisboa, 2829-516 Caparica, Portugal

## Abstract

Two families of mononuclear gold­(I) complexes featuring Au-chromophore units, with chromophores being carbazole (**a**), phenanthrene (**b**), or dibenzofuran (**c**), were synthesized. The Au­(I) atoms are coordinated to two phosphanes, either PMe_2_Ar^Xyl2^ (Ar^Xyl2^ = 2,6-C_6_H_3_-(2,6-C_6_H_3_–Me_2_)_2_) (**P1**) or the bulkier PCyp_2_Ar^Xyl2^ (Cyp = cyclopentyl) (**P2**). The photophysical properties of these complexes were extensively studied, with a particular focus on the effects of phosphane bulkiness and chromophore electron-donating capacity on triplet state quantum yields (Φ_T_). Nanosecond-laser flash photolysis measurements were employed to calculate Φ_T_. Time-dependent density functional theory (TD-DFT) calculations supported the absorption and emission assignments, providing insights into the electronic state gaps involved in photophysical processes and their relative populations. The parent complex AuCl**P2** in combination with NaBAr_4_^F^, as a chloride scavenger, served as an efficient catalyst for the hydroamination of a variety of alkynes and amines, under mild conditions and with low Au loading (0.1–0.2 mol %). Luminescent studies allowed us to check the active catalytic species.

## Graphical abstract

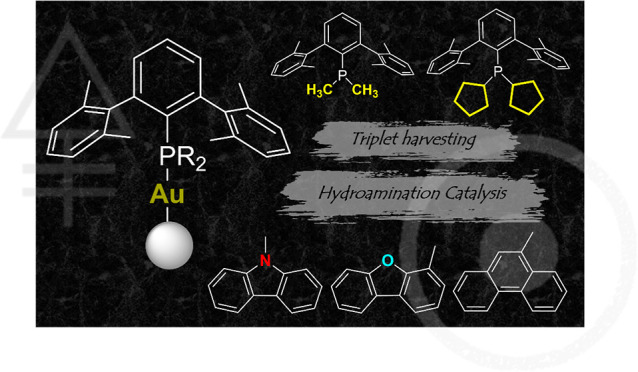

## Introduction

Phosphorescence emission is strongly sought due to its wide range of applications such as in photodynamic therapy,^1^ oxygen sensing,^2^ light-emitting diodes (PhOLEDs),^3,4^ or photon upconversion,^5,6^ among others. To achieve this kind of emission, it is mandatory to control and understand the formation of T_*n*_ states, which are responsible for the phosphorescence in the first step, and later the ratio between the rates for radiative and nonradiative decay of the triplets returning to the ground state.

When the phosphorescent emitters are incorporated in electrically excited devices (e.g. light emitting devices, LEDs), the triplet states can significantly increase the internal quantum efficiency of the device emission due to the spin-statistics, since 75% of the excitons are known to populate triplet states, compared with the 25% that produce S_*n*_ states.^7^

To populate the triplet state photophysically, we need to enhance the intersystem crossing transition (S_1_ → T_*n*_), since only the S_0_ → S_1_ is allowed upon optical excitation. The intersystem crossing transition is favored by the presence of heavy metal atoms, that increase the spin–orbit coupling and facilitate the transfer of the S_1_ excitons obtained by optical excitation to T_*n*_ states. Specifically, we use gold­(I) for this purpose, as it is known to exhibit the highest spin–orbit coupling among the d-block metals.^8–11^

Gold­(I) compounds can also display the so-called metallophilic interactions,^12–14^ which can affect their photophysical properties.^15,16^ In our group, we have recently investigated the influence of aurophillic contacts (Au···Au) and its nature (either intra- or intermolecular) in the harvesting of the T_*n*_ states.^17^

In this work, we aim to explore new methods for modulating the population of T_*n*_. To achieve this, we prepared two families of compounds with the structure chromophore-Au-PR_3_ and their AuCl**Px** precursors (**Px** = **P1** and **P2**). The chromophores used are carbazole (Cz), dibenzofuran (Fu), and phenanthrene (Phen), selected for their varying electron-donating abilities to study their effects on the promotion of T_*n*_. The luminescent ligands carbazole, phenanthrene, and dibenzofuran were chosen for their distinct electronic properties to systematically study their impact on photophysical behavior. Carbazole, as a strong electron donor, enhances conjugation and triplet state formation but lowers fluorescence quantum yields due to efficient intersystem crossing (ISC). Phenanthrene, with moderate electron-donating ability, balances fluorescence and phosphorescence, offering insight into S_1_-T_1_ transitions. Dibenzofuran, the weakest electron donor, shows the highest fluorescence yields by favoring radiative decay pathways and minimizing ISC.

Additionally, we used two different phosphanes with varying steric volumes, PMe_2_Ar^xyl2^ (**P1**) and PCyp_2_Ar^xyl2^ (**P2**), to examine how the ligand bulkiness influences intermolecular interactions and, consequently, the population of T_*n*_. In our previous work,^17^ we demonstrated that the conformation of phosphanes could promote either intramolecular or intermolecular aurophilic contacts due to the specific arrangement of the molecules. In the current study, we will investigate the influence of the phosphane bulk on the T_*n*_ harvesting process to determine if this factor is crucial for optimizing the triplet state population.

Moreover, electron-rich ancillary ligands with high steric demand have proven to be superior in gold­(I)-catalyzed hydroamination reactions.^18,19^ In particular, ligands with a pendant group that stabilizes the gold center through secondary interactions enhance substantially the catalytic activity of the Au­(I) complexes. Thus, highly competent gold­(I) catalysts with phosphane^20^ or NHC^21^ ancillary ligands have been designed, which enable the hydroamination of primary amines and alkynes at catalyst loadings lower than 0.5 mol % under mild reaction conditions. Terphenyl phosphanes can stabilize low-coordinate structures by adopting hemilabile coordination modes involving weak M···C_arene_ interactions with a flanking aryl ring of the terphenyl moiety.^22^ Herein, we demonstrate the excellent catalytic performance of our gold­(I) compounds in the intermolecular alkyne hydroamination.^23,24^ Furthermore, emission experiments allowed us to verify the stability of the catalyst during the reaction. This additional exploration aims to expand the potential applications of these gold­(I) compounds beyond their photophysical properties.

## Results and Discussion

### Synthesis and Characterization

Two families of mononuclear gold­(I) compounds with phosphanes displaying different levels of bulkiness have been synthesized following a two-step synthesis. Initially, the phosphane–Au–Cl derivatives were prepared by the reaction of AuCl­(tht) and the corresponding phosphane (Scheme S1).

In the second step, AuCl**P1** and AuCl**P2** were reacted with three chromophores that present different electron-donating or electron-withdrawing properties (carbazole (**a**), phenanthrene (**b**), dibenzofuran (**c**)) following two different reaction conditions displayed in Scheme 1.

**Scheme 1:**
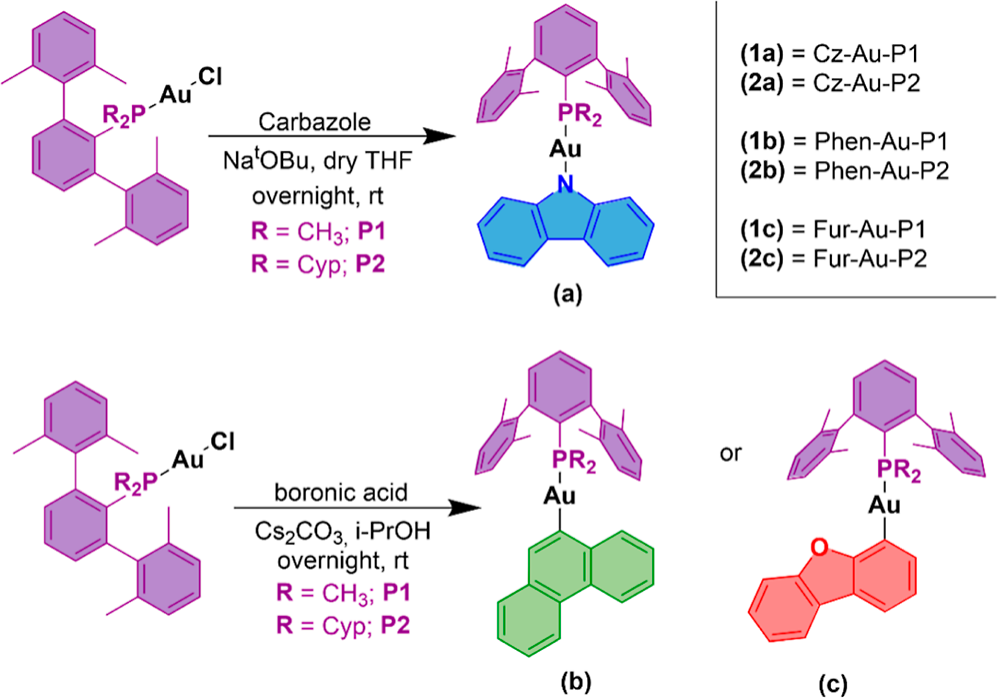
Synthesis of gold­(I) Complexes **1a**–**c** and **2a**–**c**

For the synthesis of **1a** and **2a**, the precursor AuCl**Px** was dissolved together with the carbazole ligand **La** in the presence of NaO^*t*^Bu and the reaction mixture was allowed to react overnight at room temperature in dry THF. However, the coordination of either phenanthrene (**b**) or dibenzofuran (**c**) to the metal center was achieved following our previously reported procedure.^17,25^ A solution of the corresponding boronic acid derivative with the AuCl**Px** compound was reacted in the presence of Cs_2_CO_3_ in 2-propanol at room temperature overnight (see Experimental Section for details). All the compounds were characterized by ^1^H, ^13^C­{^1^H}, and ^31^P­{^1^H} NMR spectroscopy and mass spectroscopy (see Supporting Information).

Single crystals suitable for X-ray diffraction analysis were successfully grown for **1a**, **1b**, **2b**, and **2c** (Figure 1) from slow diffusion of hexane into dichloromethane solutions of the compounds at room temperature. The crystal data and structure refinement can be found in Table S1, and the selected bond distances and angles are displayed in Table 1.

**Figure 1:**
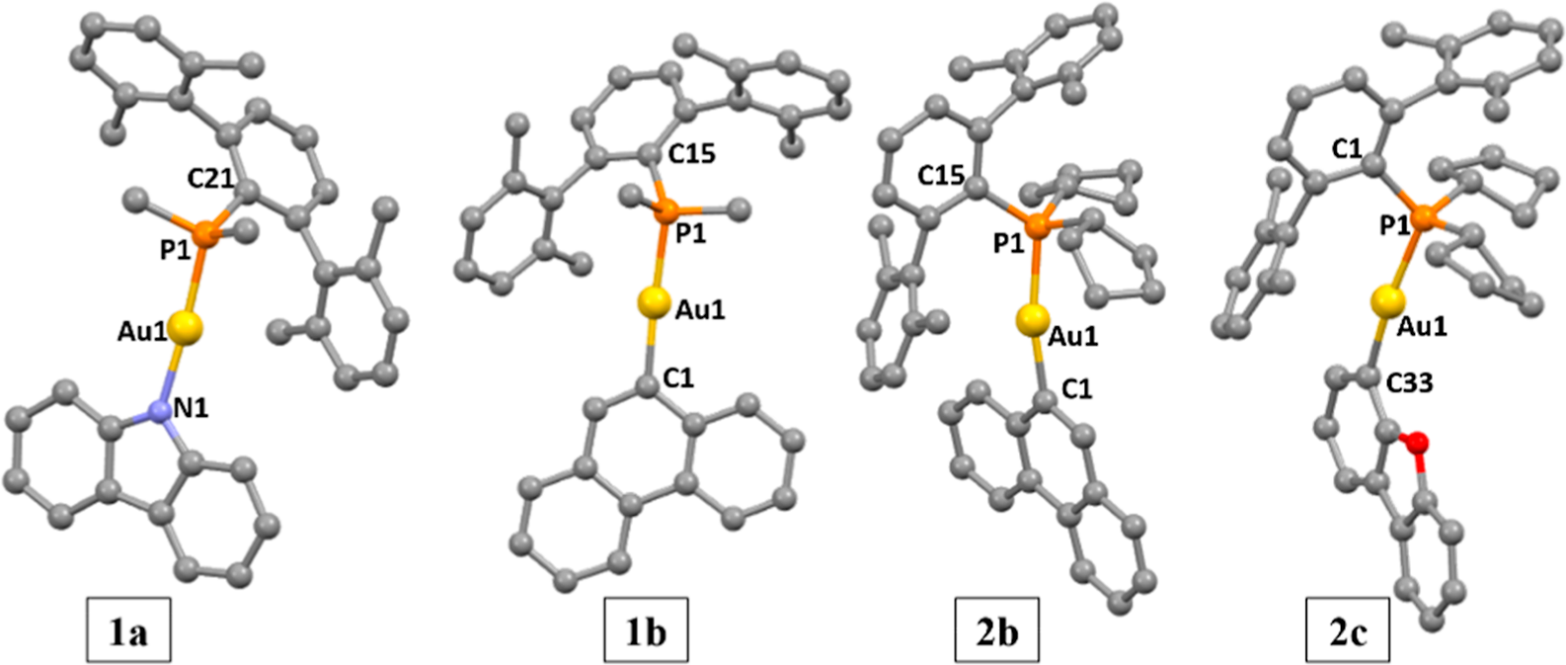
X-ray crystal structures of gold­(I) compounds **1a**, **1b**, **2b**, and **2c**. Yellow: gold; orange: phosphorus, blue: nitrogen, red: oxygen; gray: carbon. Thermal ellipsoids at 50% probability and hydrogen atoms were omitted for clarity.

**Table 1:** Selected Bond Lengths (Å) and Angles (°) for Complexes **1a**, **1b**, **2b**, and **2c**

**1a**		**1b**		**2b**		**2c**	
Distances (Å)							
N1–Au1	2.044(2)	C1–Au1	2.070(4)	C1–Au1	2.06(1)	C33–Au1	2.054(7)
Au1–P1	2.248(8)	Au1–P1	2.301(9)	Au1–P1	2.306(2)	Au1–P1	2.299(2)
Angles (°)							
N1–Au1–P1	175.99(7)	C1–Au1–P1	173.5(1)	C1–Au1–P1	168.7(3)	C33–Au1–P1	171.7(2)
Au1–P1–C21	114.06(9)	Au1–P1–C15	118.3(1)	Au1–P1–C15	122.4(3)	Au1–P1–C1	120.6(2)

The coordination of the gold­(I) unit to the corresponding atom from the fluorophore is almost linear with slightly distorted C–Au–P or N–Au–P angles from 168° to 176°, with compound **2b** being the most distorted one and the carbazole derivative **1a** the most linear. The corresponding packing of the molecules is, as expected, being affected by the bulkiness of the phosphanes. π···π and C–*H*···π weak intermolecular contacts between the carbazole groups and aromatic rings of the phosphane can be detected in compounds **1a** and **1b** (Figures S38 and S39). Meanwhile, the X-ray crystal structures of the compounds containing the bulkier phosphane (**P2**) shows dimeric assemblies in a head-to-tail conformation, where two molecules interact through C–*H*···π contacts as can be seen in Figure 2. No metallophilic intermolecular interactions were detected for none of the compounds independently of the bulkiness of the phosphane.

**Figure 2:**
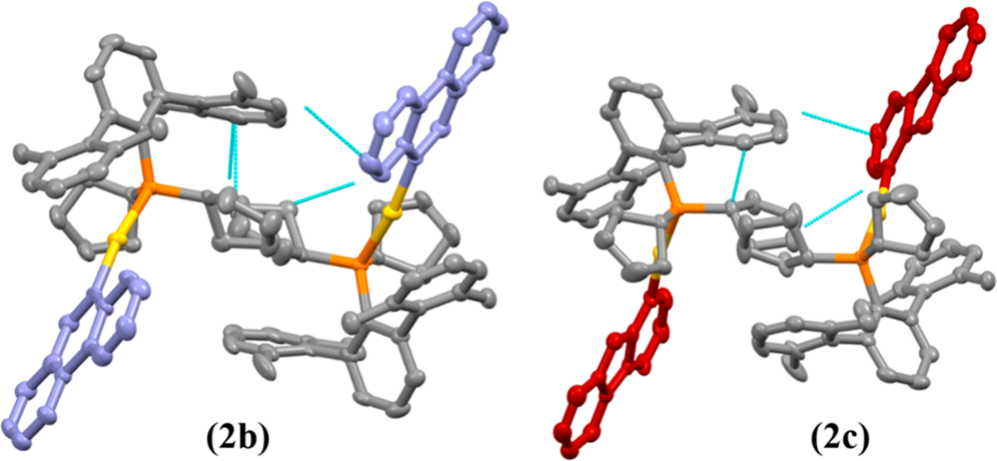
3D crystal packing and intermolecular interactions present at complexes **2b** and **2c** viewed down the *b*-axis. Yellow: gold; orange: phosphorus, blue: nitrogen, gray: carbon. Thermal ellipsoids at 50% probability and hydrogen atoms were omitted for clarity. The chromophores have been colored in order to clearly see the head-to-tail conformation. Dashed lines represent the C–*H*···π contacts.

### Photophysical Characterization

The absorption and emission spectra of all the compounds **1**(**a**–**c**) and **2**(**a**–**c**) were recorded in dichloromethane solutions at room temperature. The obtained data are summarized in Table 2. The electronic absorption spectra of all the complexes show an intense band at ca. 310 nm for La and Lb ligands and at ca. 290 nm for Lc. These bands are red-shifted upon coordination to the metal center (Figure 3). These transitions can be assigned to ligand-centered π–π* transitions according to the literature.^26–28^ The higher energy absorption band around 260 nm is ascribed to the **P** **x** moieties (see Figure S40). All compounds display a well-resolved emission band at 377 nm for **a** and **b** derivatives and at 340–350 nm for **c** derivatives ascribed to ligand-to-ligand charge transfer fluorescence transitions (see below theoretical calculations)^17,28–30^ (Figure 3). The emission lifetimes in the order of nanoseconds and the small Stokes’ shifts agree with this fluorescence emission assignment.

**Table 2:** Absorption and Emission Maxima and Fluorescence Quantum Yield Data of the gold­(I) Derivatives in Dichloromethane at Room Temperature

compound	absorption λ_max_ (nm), (10^4^ ε (M^–1^ cm^–1^))	fluorescence emission, λ_exc_ = 311 (a,b), 280 (c) nm; (solution, λ_max_ (nm))	fluorescence quantum yield (Φ_Fl_)	fluorescence quantum yield N_2_ sat. (Φ_Fl_)
**La**	292 (1.67)	355	0.22 (±0.02)	0.25 (±0.02)
**1a**	306 (1.26)	382	0.02 (±0.002)	0.04 (±0.003)
**2a**	308 (1.05)	380	0.03 (±0.002)	0.09 (±0.007)
**Lb**	299 (0.92)	372	0.04 (±0.003)	0.06 (±0.005)
**1b**	310 (1.42)	380	0.07 (±0.006)	0.07 (±0.006)
**2b**	311 (1.21)	376	0.07 (±0.006)	0.07 (±0.006)
**Lc**	289 (1.37)	330	0.35 (±0.03)	0.34 (±0.03)
**1c**	288 (1.64)	345	0.12 (±0.009)	0.13 (±0.01)
**2c**	288 (1.61)	345	0.14 (±0.01)	0.15 (±0.01)

**Figure 3:**
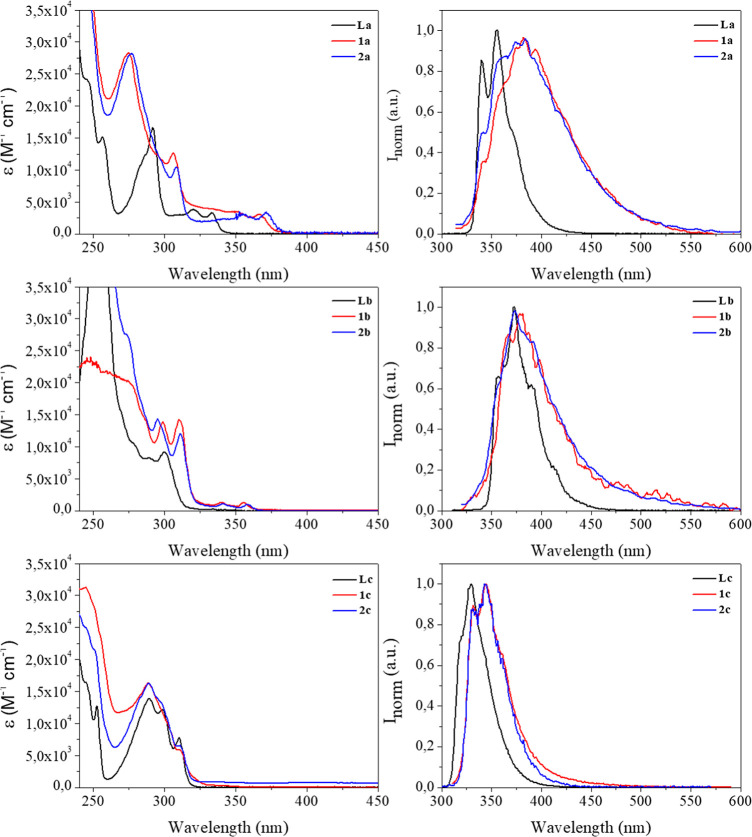
Absorption (left) and emission, λ_exc_ = 310 nm (**a** and **b** derivatives), and 290 nm (**c** derivatives) (right) spectra of dichloromethane solutions of complexes **1**(**a**–**c**) and **2**(**a**–**c**) at room temperature.

As shown in Table 2, compounds with dibenzofuran as the chromophore, **1c**, **2c**, exhibit the highest fluorescence quantum yield values (Φ_Fl_), whereas carbazole derivatives have the lowest values at room temperature. The recorded fluorescence quantum yields, and decay times are not significantly affected by the presence of oxygen in the samples (Figures S41, S42, S46–S57).

Phosphorescence emission at room temperature can be detected at ca. 480 nm only for phenanthrene derivatives (**1b** and **2b**) after removing O_2_ from the solutions (Figure 4), while the other gold­(I) complexes do not present any variation under these experimental conditions (Figures S41 and S42).

**Figure 4:**
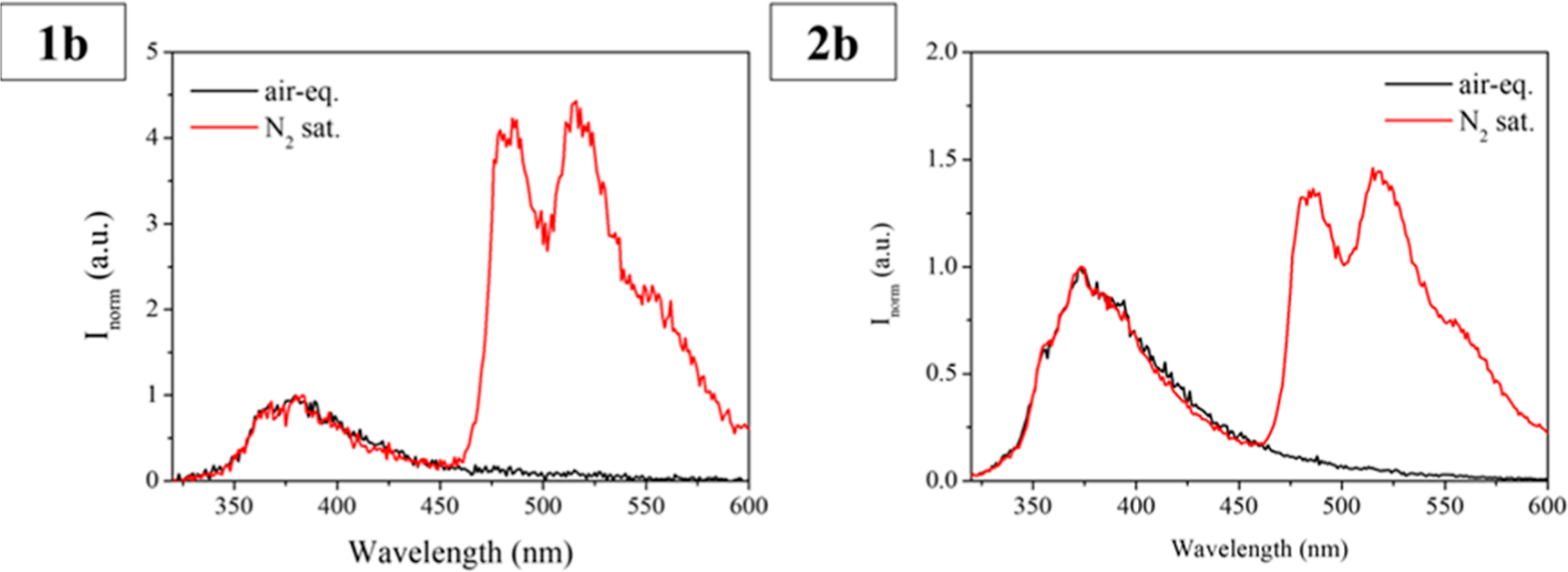
Emission spectra of N_2_ saturated dichloromethane solutions of complexes **1b** (left) and **2b** (right) at room temperature. λ_exc_ = 311 nm.

At low temperatures, the emission of all gold­(I) complexes is dominated by the phosphorescence band (see Figure S43), indicating that despite the absence of phosphorescence at room temperature, all complexes have a substantial increase in the triplet state population when compared to the organic precursors **L**(**a**–**c**). This is due to enhanced intersystem crossing from the lowest singlet excited state to the triplet manifold which is also responsible for the significant decrease in the observed fluorescence quantum yields of the complexes relative to **L**(**a**–**c**). Absorption and emission spectra were also recorded for the **Px** and AuCl**Px** precursors (see Figure S44). The **Px** ligands exhibit emission bands at 353 and 365 nm for **P1** and **P2**, respectively. These emissions are significantly quenched upon coordination to the Au­(I) center in the AuCl**Px** compounds. As expected, intersystem crossing (ISC) processes are much more efficient in the AuCl**Px** compounds. This is evidenced by the spectra recorded at 77 K, where the gold­(I) complexes show pure phosphorescence, in contrast to the dual emission observed for the uncoordinated **Px** ligands.

### Triplet Formation Quantum Yield

Quantum yields of triplet formation, Φ_T_, and triplet decay times in the presence and absence of oxygen were measured using nanosecond transient absorption, and the results are summarized in Table 4. The steric properties of both phosphanes have been calculated with the percent buried volume (%*V*_bur_) parameter and the topographic steric maps using the *SambVca 2.1* web application^31–33^ (Figure S45) for comparison purposes since we aimed to determine whether its bulkiness could affect the photophysical parameters with impact in the triplet state population. The rate constants related to the intersystem crossing (*k*_ISC_) and the S_0_ ← S_1_ internal conversion transition (*k*_IC_) have been calculated (see Supporting Information, eqs 4 and 5). The steric maps are viewed down the *z*-axis, and the orientation of ligands is also indicated in the figure. The red and blue zones show the more- and less-hindered parts in the ligand, respectively. As can be seen, **P2** is significantly bulkier in comparison to **P1**, with a %*V*_bur_ of 42.9 and 34.2%, respectively. As expected, the areas more hindered are those where the substituents CH_3_ or Cyp are localized.

The transient decay times recorded for the gold­(I) complexes (Figures S57–S68) are in the range of nanoseconds when oxygen is present in the solution and increase to the microseconds range (Table 4 and Supporting Information) in the absence of oxygen, confirming efficient quenching of the triplet state by molecular oxygen, being potential candidates for producing singlet oxygen (see below).

From the decay times in Table 3 and the triplet quantum yields in Table 4, it is possible to calculate the values for the intersystem crossing rate constant, *k*_ISC_, as well as the values for the rate constant of internal conversion between S_1_ and S_0_. There is a clear correlation between *k*_ISC_, and *k*_IC_ in the large majority of the cases, but **1a** represents a notable exception with a very large internal conversion rate constant. This could suggest that there is a similar contribution from molecular vibrations to facilitate both singlet relaxation pathways: the internal conversion for the ground state and the intersystem crossing to the triplet state, also observed for metal-free organic molecules.^34^

**Table 3:** Fluorescence Decay Times Values of Aerated Solutions and N_2_-Saturated Solutions in DCM Recorded for all the Compounds

Compound	τ (air-equilibrated) (ns)	τ (N_2_) (ns)
**1a**	6.2 (±0.3)	5.9 (±0.3)
**2a**	7.6 (±0.4)	9.3 (±0.5)
**1b**	11.4 (±0.6)	14.0 (±0.7)
**2b**	12.9 (±0.6)	16.7 (±0.8)
**1c**	2.3 (±0.1)	3.0 (±0.1)
**2c**	3.2 (±0.2)	3.6 (±0.2)

**Table 4:** Quantum Yields of Triplet Population for the Dichloromethane Solutions (Φ_T_), Decay Times in Air-Equilibrated, τ_T_(O_2_), and Nitrogen Saturated, τ_T_(N_2_), Dichloromethane Solutions; Rate Constants for Intersystem Crossing (*k*_ISC_) and Singlet Internal Conversion to the Ground State (*k*_IC_)

compound	Φ_T_	τ_T_ (O_2_) (ns)	τ_T_ (N_2_) (μs)^b^	*k*_ISC_ (10^6^ s^–1^)	*k*_IC_ (10^6^ s^–1^)
**1a**	18.9	30	8.7	32.0	131.2
**2a**	44.3	50	5.5	47.6	50.7
**1b**	29.0^a^	323	15.5	20.7	46.1
**2b**	43.0^a^	187	5.5	25.8	30.2
**1c**	47.1	150	1.5	157.0	133.0
**2c**	37.2	183	0.4	103.3	134.2

a Quantum yield of triplet formation estimated from the quantum yield of singlet oxygen production (see below).

b These values are in microseconds while those in air-equilibrated conditions are in nanoseconds.

We anticipate that the rate constant for intersystem crossing (*k*_IC_) is influenced by the bulk and rigidity of the phosphanes, but we could also expect an impact in *k*_ISC_, since it was shown that intermolecular gold–gold interactions can affect the intersystem crossing.^35^ While the first phenomenon occurs because steric hindrance likely restricts some vibrations within the medium, resulting in a diminished contribution to the vibrational relaxation process, the latter is related to the steric hindrance for the formation of intermolecular gold···gold contacts.

In the case of **a** and **b** it is clear that the bulkier the phosphane, the smaller the corresponding *k*_IC_, with a small impact in *k*_ISC_, while in the case of **c**, the largest phosphane has an impact in a decrease of *k*_ISC_, which could be evidence for some degree of intermolecular association affecting the retrieved constants.

### Singlet Oxygen Production

The efficient quenching of the triplet state of the gold­(I) complexes by molecular oxygen is generally related to energy transfer leading to ^1^O_2_ photosensitization or the presence of other mechanisms involving oxygen, e.g. electron transfer. The direct measurement of the characteristic ^1^O_2_ emission at 1270 nm, which belongs to ^1^Δ_g_ → ^3^Σ_g_^–^ transition, was used as a direct proof of this photosensitization process.

To do that, the samples were excited at λ_exc_ = 311 nm in air-equilibrated dichloromethane solutions and using perinaphthenone as the reference (Φ_Δ_ = 79%). Dibenzofuran complexes (**1c** and **2c**) have technical difficulties that make not possible the measurement of the ^1^O_2_ production.

The triplet formation quantum yields of the phenanthrene gold­(I) complexes were not accessible through ns-laser flash photolysis measurements using the depletion method (strong overlap between ground state bleaching and triplet absorption) but were able to display a significant production of ^1^O_2_ under our experimental conditions, with Φ_Δ_ values of 29% for **1b** and 43% for **2b** (Figure S69).

The measured Φ_Δ_ values are always in the lower limit of the Φ_T_ of the photosensitizer, i.e., assuming an efficiency of energy transfer to oxygen close to unity yields Φ_Δ_ = Φ_T_, and lower efficiencies lead to higher values of Φ_T_.^36^

### Theoretical Calculations

TD-DFT calculations were conducted for all compounds to optimize geometries without restraints, and harmonic frequency calculations confirmed converged structures as potential-energy minima. These calculations were performed with continuum solvation in dichloromethane, primarily to identify the lower singlet and triplet states of the molecules. All compounds present monoexcitations involving the highest occupied molecular orbital (HOMO) and the lowest unoccupied molecular orbital (LUMO). Figures S70–S75 show these molecular orbitals for all of them, and Tables S3–S5 summarize the obtained results.

It is anticipated that the most significant intersystem crossing transitions will occur between the S_1_ state and the energetically closest T_*n*_ state with suitable symmetry^37^ (see Tables S3–S5, where the most probable T_*n*_ state is highlighted in bold). The corresponding values from TD-DFT calculations are also presented in Table 5.

**Table 5:** Calculated Energy Differences between S_1_–T_1_ and S_1_–T_*n*_ ^a^

compound	Δ*E*(S_1_–T_1_) (meV)	Δ*E*(S_1_–T_*n*_) (meV)	*k*_ISC_ (10^6^ s^–1^)
**1a**	173.5	23.8	32.0
**2a**	165	24.6	47.6
**1b**	1102.6	171.1	20.7
**2b**	1089.8	192.4	25.8
**1c**	1121.2	3.1	157.0
**2c**	1112.7	6.1	103.3

a The values of *k*_ISC_ have been extracted from Table 4 for clarity.

Interestingly, compounds with larger values of Δ*E*(S_1_–T_1_) also exhibit higher fluorescence quantum yields (Φ_Fl_). This observation aligns with less efficient intersystem crossing in compounds with a larger energy gap, thereby increasing the probability of S_1_ returning to S_0_ through an emissive decay.

There is a clear correlation observed in Table 5 between the smaller singlet–triplet energy gap (Δ*E*(S_1_–T_*n*_)) and the more efficient ISC process. That is, *k*_ISC_ dibenzofuran (**c**) > carbazole (**a**) > phenanthrene (**b**), while the Δ*E*(S_1_–T_n_), for dibenzofuran (**c**) < carbazole (**a**) < phenanthrene (**b**). However, due to the complex interplay of vibrational modes and internal conversion, this correlation does not straightforwardly translate into an increase in Φ_T_ across all cases. Conversely, the S_1_–T_1_ energy gap shows a weak correlation with *k*_ISC_, suggesting that intersystem crossing likely occurs between S_1_ and higher triplet states.

### Catalytic Studies

The dual utility of chloride gold­(I) complexes (AuCl**P1** and AuCl**P2**) is a cornerstone of this study, bridging photophysical applications and catalytic functionality. The chloride complexes serve not only as precursors for the luminescent compounds but also demonstrate exceptional activity in hydroamination catalysis. This dual functionality underscores the versatility of the complexes and highlights the critical role of the bulky phosphane ligands. To assess the impact of the steric profile of terphenyl phosphanes on gold-catalyzed alkyne hydroamination, we screened the catalytic competency of the gold AuCl**Px** compounds^38^ in the hydroamination of phenylacetylene with aniline (Table 6).

**Table 6:**
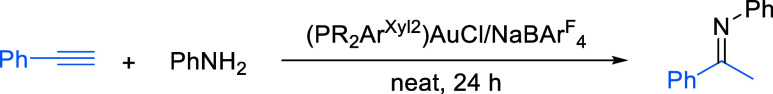
Catalytic Activity of AuCl­(PR_2_Ar^Xyl2^) in the Hydroamination of Phenylacetylene with Aniline^a^

entry	catalyst	Au (mol %)	*T* (°C)	conversion (%)	TON
1	**AuClP1**	0.1	50	59	590
2	AuCl**P2**	0.1	50	98	980
3	AuCl**P2**	0.1	25	99 (96)^b^	960
4	AuCl**P2**	0.05	25	86	1720
5	AuCl**P2**	0.05	50	99 (94)^b^	1980
6	Y_Mes_PCy_2_AuCl/NaBAr_4_^F^	0.05	50	97	1950^c^
7	(CB_11_Cl_11_)P*i*Pr_2_Au(THT)	0.01	50	>95	>9500^d^
8	(NHC)AuCl/NaBAr_4_^F^	0.01	80	76	7600^e^

a Reaction conditions: phenylacetylene (5 mmol), aniline (5 mmol), Au­(I) complex: NaBAr_4_^F^ = 1:1, reaction time = 24 h (unoptimized). Conversions determined by GC using dodecane as an internal standard.

b Yields of isolated products are given in brackets.

c From ref 20c.

d From ref 20a (reaction time 16 h).

e From ref 21 (Au­(I) complex: NaBAr_4_^F^ = 1:16; reaction time = 37 h).

The reactions were performed in the presence of equimolar amounts of the gold complex and the chloride scavenger NaBAr_4_^F^ (Ar^F^ = 3,5-(CF_3_)_2_C_6_H_3_), using reaction conditions similar to those reported for other ligands for ease of comparison.^20,21^ We found that both AuCl**Px** complexes were active in the hydroamination of a 1:1 neat mixture of the alkyne and the amine with 0.1 mol % of the gold catalyst and NaBAr_4_^F^ at 50 °C (Table 6, entries 1 and 2). However, only complex AuCl**P2** supported by the bulkier terphenyl phosphane, PCyp_2_Ar^Xyl2^ provided complete conversions. Consequently, further catalytic tests were conducted only with AuCl**P2**. We observed excellent conversions when the hydroamination was performed at room temperature with 0.1 mol % AuCl**P2**/NaBAr_4_^F^ (entry 3). A further reduction of the catalyst loading to 0.05 mol % still provided the imine in a high 86% yield, albeit to reach full conversion the mixture required heating at 50 °C (entries 4 and 5). The catalytic activity displayed by AuCl**P2**/NaBAr_4_^F^ under such mild reaction conditions compares well to those observed for the most efficient Au­(I) catalysts under similar conditions (entries 6–8).

We examined the scope of the hydroamination reactions with different alkynes and a variety of aryl amines under the optimized reaction conditions (Scheme 2). Both, electron-rich and electron-deficient anilines added to phenylacetylene at room temperature affording the corresponding imines in good to high yields (Scheme 2, **ab**–**ad**). Sterically hindered anilines such as mesitylamine and 2,6-diisopropylphenylamine were also suitable substrates for the addition of phenyl acetylene at 50 °C (**ae** and **af**). Regarding the alkyne scope, *p*-substituted phenylacetylenes with different electronic demands, as well as alkylacetylenes underwent hydroamination with aniline, although reactions of the latter required increasing the catalyst loading to 0.2 mol % (**ag**–**aj**). Diphenylacetylene, a reluctant substrate that usually requires high catalyst loadings and harsher reaction conditions to accomplish this transformation,^39^ could be successfully functionalized using 0.2 mol % catalyst at 80 °C, providing the expected hydroamination product in good yield as a 3:1 mixture of the imine and enamine derivatives (**ak**). Finally, we sought to test the reactivity of the gold catalysts in the amination of phenylacetylene with challenging secondary amines.^39b,39c^ The corresponding enamines were obtained in good yields with 2.5 mol % of gold catalyst (**al**, **am**). With *N*-methylaniline the reaction yielded exclusively the Markonikov adduct, whereas with piperidine a mixture 1:3 of the Markonikov and anti-Markonikov products was obtained. Overall, these results demonstrate the excellent performance of the terphenyl phosphane ligand PCyp_2_Ar^Xyl2^ in gold-catalyzed hydroamination of alkynes.

**Scheme 2:**
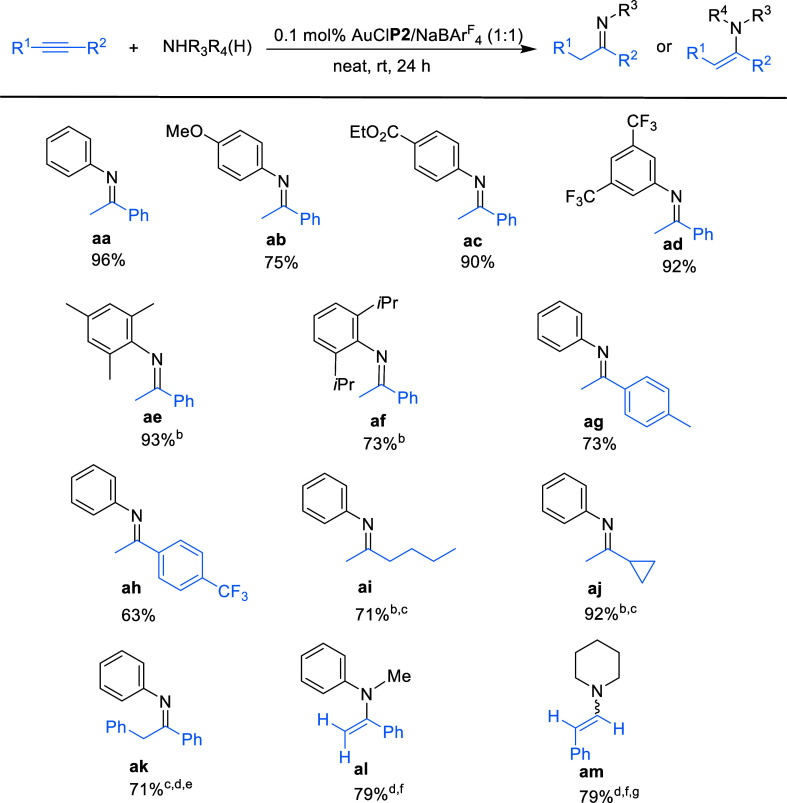
Intermolecular Hydroamination of Alkynes with Primary and Secondary Aryl Amines Catalysed by AuCl**P2**/NaBAr_4_^F^

a Reaction conditions: phenylacetylene (2.5 mmol), aniline (2.5 mmol). Yields of isolated product.
b
*T* = 50 °C.
c Catalyst loading (0.2 mol %).
d
*T* = 80 °C.
e Selectivity imine/enamine 3:1 determined by ^1^H NMR.
f Catalyst loading (2.5 mol %).
g Selectivity *gem*/*E*/*Z* = 1:1.4:1.4 determined by ^1^H NMR.

The catalytic reaction was followed by emission spectroscopy in order to monitor the catalyst stability over time (see Figure S76). As stated above, the demetalation of the catalyst should induce an increase on the emission intensity of the phosphane, what would indicate the degradation of the catalyst along the reaction process. The reason for this emission enhancement is that the loss of gold­(I) leads to a decrease on the quenching mechanism imposed by the intersystem crossing. As we did not observe any increase in the emission intensity, this means that the phosphane is kept coordinated to the metal center without degradation. Additionally, if phosphane decoordination occurs, it should be accompanied by the reduction of Au­(I) to Au(0), which is readily observable to the naked eye. The persistence of the yellow color throughout the reaction, along with its intensification during the formation of the final product, further supports the stability of the AuCl**Px** catalyst.

By modulating steric hindrance through these ligands, we observed significant effects on both photophysical parameters, such as quantum yield (Φ_T_) and intersystem crossing rates (*k*_ISC_), and catalytic efficiency. For instance, **P2**, with its bulkier cyclopentyl substituents, enhances the stabilization of intermediates in hydroamination reactions, leading to superior catalytic performance even under mild conditions. Simultaneously, the steric bulk influences the suppression of nonradiative decay pathways in photophysical processes, allowing higher triplet state populations. Thus, the interconnection lies in the shared dependency of both applications on ligand steric and electronic properties. Increasing steric bulk provides a dual advantage by optimizing both photophysical performance and catalytic functionality.

## Conclusions

This work highlights the dual role of bulky gold­(I) complexes as both efficient phosphorescent materials and robust hydroamination catalysts. The X-ray structures obtained for compounds **1a**, **1b**, **2b**, and **2c** revealed the significant role of the presence of two different monophosphanes with varying bulkiness in the 3D packing of gold­(I) derivatives. The bulk of these monophosphanes was found to influence molecular vibrations in the medium, resulting in differences in the *k*_IC_ rate constants, typically higher for the smaller phosphane.

Three chromophores with distinct electron-donating capabilities were utilized: carbazole (**a**), phenanthrene (**b**), and dibenzofuran (**c**). All gold­(I) compounds exhibited fluorescence emission at room temperature, while only phenanthrene derivatives showed phosphorescence under nitrogen-saturated conditions. However, all gold­(I) compounds demonstrated phosphorescence when cooled to 77 K.

Φ_T_ was measured for all complexes, with Φ_Δ_ taken rigorously as Φ_T_ for phenanthrene derivatives. Rate constants *k*_IC_ and *k*_ISC_ were calculated, with compounds featuring dibenzofuran as the chromophore displaying the highest *k*_ISC_ values.

The stabilization of the gold­(I) center through steric bulk ensures excellent activity and selectivity in hydroamination reactions, with potential implications for broader catalytic applications. By elucidating these connections, this study not only advances the understanding of gold­(I) complexes but also provides a framework for their application in interdisciplinary research.

All in all, it can be said that (i) The bulky phosphane ligands enhance triplet harvesting by modulating intersystem crossing rates and suppressing nonradiative decay pathways; (ii) the stabilization of the gold­(I) center through steric bulk ensures excellent activity and selectivity in hydroamination reactions, with potential implications for broader catalytic applications; (iii) the interplay of steric and electronic effects in ligand design offers a pathway to multifunctional complexes for advanced material and catalytic applications.

By elucidating these connections, this study not only advances the understanding of gold­(I) complexes but also provides a framework for their application in interdisciplinary research.

## Experimental Section

### General Procedures

All manipulations have been performed under prepurified N_2_ using standard Schlenk techniques. All solvents have been distilled from appropriate drying agents. Commercial reagents dibenzofuran-4-boronic acid and 9-phenanthreneboronic acid were purchased from Fluorochem, while cesium carbonate carbazole and NaO^*t*^Bu were purchased from Merck. Phosphanes PMe_2_Ar^Xyl2^,^40^ **P1** and PCyp_2_Ar^Xyl2^,^22a^ **P2** were prepared by previously reported procedures.

### Crystal Data

Data for compounds **1a**, **1b**, **2b**, and **2c** were collected at BL13-XALOC beamline^41^ of the ALBA synchrotron (λ = 0.72931) at 100 K. Crystals were mounted with Paratone N grease on a MiTegen kapton loop and placed in the N_2_ stream of an Oxford Cryosystems Cryostream. The structures were solved by intrinsic phasing with SHELXT^42^ and refined by full-matrix least-squares on *F* ^2^ with SHELXL.^43^

### Physical Measurements

Infrared spectra have been recorded on a Fourier transform infrared (FT-IR) 520 Nicolet Spectrophotometer. ^1^H NMR (δ­(TMS) = 0.0 ppm) and ^31^P­{^1^H} NMR (δ­(85% H_3_PO_4_) = 0.0 ppm) spectra have been obtained on a Bruker 400. ES­(+) mass spectra were recorded on a Fisons VG Quatro spectrometer. Absorption spectra have been recorded on a Varian Cary 100 Bio UV spectrophotometer, and emission spectra have been recorded on a Horiba Jobin-Yvon SPEX Nanolog spectrofluorimeter. Quantum yields have been recorded on a Hamamatsu Absolute PL Quantum Yield Spectrometer C11347. Luminescence lifetimes were measured on JYF-DELTAPRO-NL equipment upon excitation of the samples with a 285 nm NanoLED and collecting the decays through a bandpass filter of 340, 400, or 500 nm.

Transient absorption experiments were measured with a laser flash photolysis LK60 Applied Photophysics system in absorption mode after laser pulse excitation at 355 nm (for **1a**, **2a** and **1b**, **2b**) and 266 nm (for **1c**, **2c**) at the Departamento de Quimica-Universidade Nova de Lisboa.

### Theoretical Calculations

Density functional calculations were carried out by using the GAUSSIAN package.^44^ The geometries of all compounds in this study were fully optimized at the TZVP level of theory using Gaussian09. The hybrid density function method known as B3LYP was applied.^45,46^ Effective core potentials (ECP) were used to represent the innermost electrons of the gold atom and the basis set of valence triple-ζ quality with an extra d-polarization function.^47^ A similar description was used for all main group elements.^48^ Atomic charges and population analysis have been confirmed from the analysis of natural bond order.^49^ The solvent effects of dichloromethane were taken into account by PCM calculations,^50^ keeping the optimized geometries for the gas phase without symmetry restrictions. Excited states and absorption spectra were obtained from the time-dependent algorithm implemented in Gaussian09.^51^

### Synthesis and Characterization

#### Synthesis of [AuCl­(PMe_2_Ar^Xyl2^)], AuCl**P1**

AuCl­(tht) (236.1 mg, 0.74 mmol) and P1 (PMe_2_Ar^Xyl2^) (255.1 mg, 0.74 mmol) were solved in a dichloromethane solution under Schlenk conditions and stirred at room temperature for 1 h. Then, the solution was concentrated, and hexane was added in order to precipitate the compound in a pure form. In the end, the solution is filtrated, and the white solid obtained is completely dried under vacuum. Yield 90% (383.9 mg). ^1^H NMR (400 MHz, CDCl_3_, 25 °C, δ): 7.60 (td, 1H, ^3^ *J*_HH_ = 7.5 Hz, ^5^ *J*_HP_ = 2 Hz, *p*-C_6_H_3_), 7.29 (t, 2H, ^3^ *J*_HH_ = 7.6 Hz, *p*-Xyl), 7.16 (d, 4H, ^3^ *J*_HH_ = 7.6 Hz, *m*-Xyl), 7.12 (dd, ^3^ *J*_HH_ = 7.6 Hz, ^4^ *J*_HP_ = 3.5 Hz, *m*-C_6_H_3_), 2.12 (s, 12H, C*H*_3_), 1.19 (d, ^2^ *J*_HP_ = 10.4 Hz, P–C*H*_3_). ^13^C­{^1^H} NMR (125 MHz, CDCl_3_, 25 °C, δ): 145.2 (d, ^2^ *J*_CP_ = 10 Hz, *o*-*C*_6_H_3_), 139.4 (d, ^3^ *J*_CP_ = 5 Hz, *ipso*-Xyl), 135.1 (*o*-Xyl), 130.9 (d, ^4^ *J*_CP_ = 3 Hz, *p*-C_6_H_3_), 130.1 (d, ^3^ *J*_CP_ = 8 Hz, *m*-*C*_6_H_3_), 127.6 (*p*-Xyl), 127.1 (*m*-Xyl), 125.6 (d, ^1^ *J*_CP_ = 57 Hz, *ipso*-C_6_H_3_), 20.8 (*C*H_3_), 16.3 (d, ^1^ *J*_CP_ = 39, P–*C*H_3_). ^31^P­{^1^H} NMR (161.9 MHz, CDCl_3_, 25 °C, δ): −3.7.

ES-MS­(+) *m*/*z*: 601.11 ([M + Na^+^]^+^, Calcd 601.11), 543.15 ([M – Cl^–^]^+^, Calcd 543.15), 889.33 ([AuP_2_]^+^, Calcd 890.34), 617.08 ([M + K^+^]^+^, Calcd 617.08), 585.14 ([M + Li^+^]^+^, Calcd 585.14).

#### Synthesis of [AuCl­(PCyp_2_Ar^Xyl2^)], AuCl**P2**

The synthesis of AuCl**P2** was performed following the same procedure as for AuCl**P1** by substitution of **P1** for **P2** (336.2 mg, 0.74 mmol). Yield 80% (576.5 mg). ^1^H NMR (400 MHz, CDCl_3_, 25 °C, δ): 7.38 (td, 1H, ^3^ *J*_HH_ = 7.6 Hz, ^5^ *J*_HP_ = 1.0 Hz, *p*-C_6_ *H*_3_), 7.16 (dd, 2H, *J*_HH_ = 8.0, 7.1 Hz, *p*-Xyl), 7.07–7.06 (m, 4H, *m*-Xyl), 7.01 (dd, 2H, ^3^ *J*_HH_ = 7.6 Hz, ^4^ *J*_HP_ = 1.7 Hz, *m*-C_6_ *H*_3_), 2.06 (s, 12H, C*H*_3_), 1.87–0.94 (m, 18H, Cyp). ^13^C­{^1^H} NMR (125 MHz, CDCl_3_, 25 °C, δ): 147.0 (d, ^2^ *J*_CP_ = 9 Hz, *o*-*C*_6_H_3_), 140.3 (d, ^3^ *J*_CP_ = 5 Hz, *ipso*-Xyl), 135.3 (*o*-Xyl), 131.2 (d, ^3^ *J*_CP_ = 7 Hz, *m*-*C*_6_H_3_), 130.1 (d, ^4^ *J*_CP_ = 2 Hz, *p*-C_6_H_3_), 127.7 (d, ^1^ *J*_CP_ = 47 Hz, *ipso*-C_6_H_3_), 127.3 (*p*-Xyl), 126.8 (*m*-Xyl), 37.1 (d, ^1^ *J*_CP_ = 35 Hz, P–*C*H), 34.1 (d, *J*_CP_ = 8 Hz, *C*H_2_), 31.4 (d, *J*_CP_ = 7 Hz, *C*H_2_), 24.1 (d, *J*_CP_ = 12 Hz, *C*H_2_), 23.8 (d, *J*_CP_ = 14 Hz, *C*H_2_), 20.5 (*C*H_3_). ^31^P­{^1^H} NMR (161.9 MHz, CDCl_3_, 25 °C, δ): 51.5. ES-MS­(+) *m*/*z*: 709.20 ([M + Na^+^]^+^, Calcd 709.20), 651.24 ([M – Cl^–^]^+^, Calcd 651.25), 692.27 ([M – Cl + ACN]^+^, Calcd 692.27).

#### Synthesis of [Au­(carbazolyl)­(PMe_2_Ar^Xyl2^)], **1a**

Carbazole (25.1 mg, 0.15 mmol) and AuClP1 (89.2 mg, 0.15 mmol) were added to a previously prepared solution of NaO^*t*^Bu (14.4 mg, 0.15 mmol) in dry THF (10 mL). The suspension was maintained under stirring at room temperature overnight. Then, the solution is filtered through Celite and evaporated until dryness. After that, the solid obtained was recrystallized with dichloromethane/hexane to obtain the pure product. Yield 52% (56.9 mg). ^1^H NMR (400 MHz, CDCl_3_, 25 °C, δ): 8.11–8.08 (m, 2H, C*H*_Ar_), 7.66 (td, 1H, ^3^ *J*_HH_ = 7.5 Hz, ^5^ *J*_HP_ = 2 Hz, *p*-C_6_ *H*_3_), 7.46–7.44 (m, 2H, C*H*_Ar_), 7.32–7.28, (m, 2H, *p*-Xyl), 7.17–7.03 (m, 10H, C*H*_Ar_, *m*-Xyl, m-C_6_ *H*_3_), 2.22 (s,12H, C*H*_3_), 1.27 (d, ^2^ *J*_HP_ = Hz, P–C*H*_3_). ^13^C­{^1^H} NMR (75 MHz, CDCl_3_, 25 °C, δ): 149.3 (C_Ar_), 146.1 (d, ^2^ *J*_CP_ = 10 Hz, *o*-*C*_6_H_3_), 140.5 (d, ^3^ *J*_CP_ = 5 Hz, *ipso*-Xyl), 136.3 (*o*-Xyl), 131.4 (*p*-*C*_6_H_3_), 131.1 (d, ^3^ *J*_CP_ = 8 Hz, *m*-*C*_6_H_3_), 128.7 (*p*-Xyl), 128.1 (*m*-Xyl), 127.1 (d, ^1^ *J*_CP_ = 55 Hz, *ipso*-*C*_6_H_3_), 124.1 (d, ^3^ *J*_CP_ = 3 Hz, C_Ar_), 123.6, 119.6, 119.2, 116.1, 113.7 (*C*_Ar_), 21.9 (*C*H_3_), 16.9 (d, ^1^ *J*_CP_ = 39, P–*C*H_3_). ^31^P­{^1^H} NMR (161.9 MHz, CDCl_3_, 25 °C, δ): −0.9. ES-MS­(+) *m*/*z*: 710.2236 ([M + H^+^]^+^, Calcd 710.2200), 732.2052 ([M + Na^+^]^+^, Calcd 732.2100).

#### Synthesis of [Au­(carbazolyl)­(PCyp_2_Ar^Xyl2^)], **2a**

The synthesis of **2a** was performed following the same procedure as for **1a** by substitution of AuClP1 for AuCl**P2** (145.9 mg, 0.2 mmol). Yield 46% (79.9 mg). ^1^H NMR (400 MHz, CDCl_3_, 25 °C, δ): 8.08 (dd, 2H, *J*_HH_ = 7.7, 0.7 Hz, C*H*_Ar_), 7.57 (td, 1H, ^3^ *J*_HH_ = 7.6 Hz, ^5^ *J*_HP_ = 1.7 Hz, *p*-C_6_ *H*_3_), 7.30–7.25 (m, 4H, C*H*_Ar_, *p*-Xyl), 7.16 (dd, 2H, *J* = 7.6, 3.0 Hz, C*H*_Ar_), 7.02 (m, 2H, C*H*_Ar_), 6.87–6.85 (m, 4H, *m*-Xyl), 6.58 (br, 2H, *m*-C_6_ *H*_3_), 2.34–2.25 (m, 2H, Cyp), 2.14 (s, 12H, C*H*_3_), 1.95–1.84 (m, 6H, Cyp), 1.76–1.40 (m, 10H, Cyp). ^13^C­{^1^H} NMR (75 MHz, CDCl_3_, 25 °C, δ): 149.5 (C_Ar_), 148.3 (d, ^2^ *J*_CP_ = 10 Hz, *o*-*C*_6_H_3_), 140.3 (d, ^3^ *J*_CP_ = 5 Hz, *ipso*-Xyl), 135.9 (*o*-Xyl), 132.2 (d, ^3^ *J*_CP_ = 7 Hz, *m*-*C*_6_H_3_), 131.2 (*p*-*C*_6_H_3_), 128.5 (d, ^1^ *J*_CP_ = 52 Hz, *ipso*-*C*_6_H_3_),128.2 (*p*-Xyl), 127.8 (*m*-Xyl), 124 (C_Ar_), 123.2, 119.4, 115.4, 113.9 (*C*_Ar_), 38.0 (d, ^1^ *J*_CP_ = 41 Hz, P–*C*H), 35.3 (d, *J*_CP_ = 8 Hz, *C*H_2_), 32.4 (d, *J*_CP_ = 7 Hz, *C*H_2_), 25.6 (d, *J*_CP_ = 11 Hz, *C*H_2_), 25.4 (d, *J*_CP_ = 13 Hz, *C*H_2_), 21.6 (*C*H_3_). ^31^P­{^1^H} NMR (161.9 MHz, CDCl_3_, 25 °C, δ): 49.4. ES-MS­(+)*m*/*z*: 820.3272 ([M + H^+^]^+^, Calcd 820.3200), 843.3477 ([M + Na^+^]^+^, Calcd 841.3000).

#### Synthesis of [Au­(phenanthrenyl)­(PMe_2_Ar^Xyl2^)], **1b**

9-Phenanthreneboronic acid (55.3 mg, 0.25 mmol) and AuClP1 (144 mg, 0.25 mmol) were added to a previously prepared solution of Cs_2_CO_3_ (122.2 mg, 0.38 mmol) in dry 2-propanol (10 mL). The suspension was stirred overnight at room temperature. Then, the solution was filtered with Celite and evaporated until dryness. The solid obtained was then recrystallized with dichloromethane/hexane to obtain the product in a pure form. Yield 57% (102.2 mg). ^1^H NMR (400 MHz, CDCl_3_, 25 °C, δ): 8.68–8.65 (m, 1H, C*H*_Ar_), 8.62–8.60 (m, 1H, C*H*_Ar_), 8.32 (dd, 1H, *J*_HH_ = 7.5, 2 Hz, C*H*_Ar_), 7.78 (dd, 1H, *J* = 6.6, 2.0 Hz, CH_Ar_), 7.71–7.70 (m, 1H, *p-C*_6_H_3_), 7.57–7.46 (m, 5H, C*H*_Ar_), 7.23–7.21 (m, 2H, *p*-Xyl), 7.16–7.14 (m, 4H, *m*-Xyl), 7.13 (dd, ^3^ *J*_HH_ = 7.6 Hz, ^4^ *J*_HP_ = 3.0 Hz, *m*-*C*_6_H_3_), 2.22 (s, 12H, C*H*_3_), 1.14 (d, 6H, *J* = 8.1 Hz, P–C*H*_3_). ^13^C­{^1^H} NMR (75 MHz, CDCl_3_, 25 °C, δ): 173.0 (C_Ar_), 171.4 (C_Ar_), 146.0 (d, ^2^ *J*_CP_ = 10 Hz, *o*-*C*_6_H_3_), 141.6 (*C*_Ar_), 141.1 (d, ^3^ *J*_CP_ = 5 Hz, *ipso*-*C*_6_H_3_), 136.6 (*C*_Ar_), 136.5 (*o*-Xyl), 135.0 (*C*_Ar_), 132,6 (d, *J*_CP_ = 7 Hz, *C*_Ar_), 131.0 (*p*-*C*_6_H_3_), 130,8 (d, *J*_CP_ = 8 Hz, *m-C*_6_H_3_), 130.4 (d, *J*_CP_ = 6 Hz, *C*_Ar_), 130.2, 129.2 (*C*_Ar_), 128.3 (*p*-Xyl), 128.0 (*m*-Xyl), 127.8 (*C*_Ar_), 125.4 (d, ^1^ *J*_CP_ = 58 Hz, *ipso*-C_3_H_6_), 124.8, 124.6, 122.4, 22.3 (*C*_Ar_), 22.0 (*C*H_3_), 16.5 (d, ^1^ *J*_CP_ = 32 Hz, P–*C*H_3_). ^31^P­{^1^H} NMR (161.9 MHz, CDCl_3_, 25 °C, δ): 17.8. ES-MS­(+) *m*/*z*: 721.2299 ([M + H^+^]^+^, Calcd 721.2220).

#### Synthesis of [Au­(phenanthrenyl)­(PCyp_2_Ar^Xyl2^)], **2b**

The synthesis of **2b** was performed following the same procedure as for **1b** by substitution of AuClP1 for AuCl**P2** (101.9 mg, 0.14 mmol). Yield 43% (52.9 mg). ^1^H NMR (400 MHz, CDCl_3_, 25 °C, δ): 8.70 (d, 1H, *J*_HH_ = 7.9 Hz, C*H*_Ar_), 8.63 (d, 1H, *J*_HH_ = 8.4 Hz, C*H*_Ar_), 8.59 (d, 1H, *J*_HH_ = 8.0 Hz, C*H*_Ar_), 8.21 (dd, 1H, *J*_HH_ = 7.8, 1.3 Hz, C*H*_Ar_), 7.91–7.89 (m, 1H, C*H*_Ar_), 7.70–7.40 (m, 5H, C*H*_Ar,_ *p*-C_6_H_3_, *p*-Xyl), 7.10 (dd, 2H, *J* = 1.6, 2.6 Hz, C*H*_Ar_), 7.00–6.93 (m, 6H, *m*-*C*_6_H_3_, *m*-Xyl), 2.44–2.34 (m, 2H, Cyp), 2.14 (s, 12H, C*H*_3_), 2.0–1.40 (m, 16H, Cyp). ^13^C­{^1^H} NMR (75 MHz, CDCl_3_, 25 °C, δ): 177.2 (C_Ar_), 175.7 (C_Ar_), 148.7 (d, ^2^ *J*_CP_ = 10 Hz, *o*-*C*_6_H_3_), 142.0 (*ipso*-Xyl), 141. Eight (*C*_Ar_), 136.2 (*o*-Xyl), 135.6, 134.8, 132.6 (*C*_Ar_), 132.5 (d, ^1^ *J*_CP_ = 38 Hz, *ipso*-*C*_6_H_3_), 132.0 (d, ^3^ *J*_CP_ = 7 Hz, *m*-*C*_6_H_3_), 130.6 (*p*-C_6_H_3_), 130.4 (*C*_Ar_), 128.9 (*C*_Ar_), 127.9 (*p*-Xyl), 127.8 (*m*-Xyl), 127.4, 125.6, 124.5, 124.2, 124.1, 122.2 (*C*_Ar_), 38.1 (d, ^1^ *J*_CP_ = 28 Hz, P–*C*H), 34.7 (d, *J*_CP_ = 10 Hz, *C*H_2_), 32.0 (d, *J*_CP_ = 8 Hz, *C*H_2_), 25.7 (d, *J*_CP_ = 11 Hz, *C*H_2_), 25.4 (d, *J*_CP_ = 13 Hz, *C*H_2_), 21.7 (*C*H_3_). ^31^P­{^1^H} NMR (161.9 MHz, CDCl_3_, δ): 57.5. ES-MS­(+) *m*/*z*: 829.3247 ([M + H^+^]^+^, Calcd 829.3159).

#### Synthesis of [Au­(dibenzofuranyl)­(PMe_2_Ar^Xyl2^)], **1c**

The synthesis of **1c** was performed following the same procedure as for **1b** by substitution of 9-phenanthreneboronic acid for dibenzo-4-boronic acid (8.5 mg, 0.04 mmol). Yield 59% (16.8 mg). ^1^H NMR (400 MHz, CDCl_3_, 25 °C, δ): 8.06–8.02 (m, 1H, CH_Ar_), 7.98 (dd, 1H, *J*_HH_ = 7.6, 1.3 Hz, C*H*_Ar_), 7.91–7.89 (m, 1H, *p*-*C*_6_H_3_), 7.70–7.68 (m, 1H, *CH*_Ar_), 7.57–7.23 (m, 6H, C*H*_Ar_), 7.24–7.17 (m, 2H, C*H*_Ar_), 7.15–7.11 (m, 6H, *m*-C_6_ *H*_3_, *p*-Xyl), 2.25 (s, 12H, C*H*_3_), 1.14 (d, 6H, ^2^ *J*_HP_ = 8.4 Hz, P–C*H*_3_). ^13^C­{^1^H} NMR (75 MHz, CDCl_3_, 25 °C, δ): 163.9, 155.8, 152.0, 150.4 (*C*_Ar_), 146.1 (d, ^2^ *J*_CP_ = 10 Hz, *o*-*C*_6_H_3_), 141.1 (d, ^3^ *J*_CP_ = 4 Hz, *ipso*-Xyl), 137.8 (*C*_Ar_), 136.5 (*o*-Xyl), 130.9 (*p*-*C*_6_H_3_), 130.8 (d, ^3^ *J*_CP_ = 7 Hz, *m*-*C*_6_H_3_), 130.1 (d, ^1^ *J*_CP_ = 41 Hz, *ipso*-*C*_6_H_3_), 128.2 (*p*-Xyl), 127.9 (*m*-Xyl), 126.0, 125.7 (*C*_Ar_), 122.2 (d, *J*_CP_ = 5 Hz, *C*_Ar_), 121.5, 120.4, 117.1, 111.3 (C_Ar_), 22.0 (*C*H_3_), 16.5 (d, ^1^ *J*_CP_ = 32 Hz, P–*C*H_3_). ^31^P­{^1^H} NMR (161.9 MHz, CDCl_3_, 25 °C, δ): 16.4. ES-MS­(+) *m*/*z*: 711.2077 ([M + H^+^]^+^, Calcd 711.2013).

#### Synthesis of [Au­(dibenzofuranyl)­(PCyp_2_Ar^Xyl2^)], **2c**

The synthesis of **2c** was performed following the same procedure as for **1c** by substitution of AuCl**P1** for AuCl**P2** (165.5 mg, 0.23 mmol). Yield 41% (82.5 mg). ^1^H NMR (400 MHz, CDCl_3_, 25 °C, δ): 7.88–7.85 (m, 1H, C*H*_Ar_), 7.63–7.60 (m, 1H, C*H*_Ar_), 7.52 (td, 1H, ^3^ *J*_HH_ = 7.6 Hz, ^5^ *J*_HP_ = 1.7 Hz, *p*-C_6_ *H*_3_), 7.37–7.29 (m, 2H, C*H*_Ar_), 7.19–7.18 (dd, 1H, *J*_HH_ = 7.6, 1.2 Hz, C*H*_Ar_), 7.21–7.17 (m, 2H, *p*-Xyl), 7.11 (dd, 2H, *J* = 7.6, 2.7 Hz, C*H*_Ar_), 7.05 (s, 6H, *m*-Xyl, *m*-C_6_ *H*_3_), 2.39–2.29 (m, 2H, Cyp), 2.13 (s, 12H, C*H*_3_), 2.01–1.70 (m, 10H, Cyp), 1.61–1.40 (m, 6H, Cyp). ^13^C­{^1^H} NMR (75 MHz, CDCl_3_, 25 °C, δ): 164.6, 156.0, 155.7, 154.6 (*C*_Ar_), 148.7 (d, ^2^ *J*_CP_ = 10 Hz, *o*-*C*_6_H_3_), 141.9 (d, ^3^ *J*_CP_ = 5 Hz, *ipso*-Xyl), 136.9 (*C*_Ar_), 136.4 (*o*-Xyl), 132.0, 131.9 (d, ^3^ *J*_CP_ = 6 Hz, *m*-*C*_6_H_3_), 130.6 (*p*-*C*_6_H_3_), 127.8 (*p*-Xyl), 127.7 (*m*-Xyl), 127.1 (d, ^1^ *J*_CP_ = 40 Hz, *ipso*-*C*_6_H_3_), 126.1, 125.3 (*C*_Ar_), 121.8 (d, *J*_CP_ = 5 Hz, *C*_Ar_), 121.2, 120.5, 116.3, 110.9 (*C*_Ar_), 38.1 (d, ^1^ *J*_CP_ = 28 Hz, P–*C*H), 34.7 (d, *J*_CP_ = 10 Hz, *C*H_2_), 32.0 (d, *J*_CP_ = 8 Hz, *C*H_2_), 25.7 (d, *J*_CP_ = 11 Hz, *C*H_2_), 25.3 (d, *J*_CP_ = 13 Hz, *C*H_2_), 21.6 (*C*H_3_). ^31^P­{^1^H} NMR (161.9 MHz, CDCl_3_, 25 °C, δ): 56.7. ES-MS­(+) *m*/*z*: 819.3023 ([M + H^+^]^+^, Calcd 819.2952).

## Supplementary Material



## Notes

Additional details on the characterization data including NMR, X-ray crystallography and photophysical data; computational data; catalysis screening and information on the obtained compounds (PDF)
