# Supplementary material for: Gold(I) Complexes with Bulky Phosphanes: A Dual Approach to Triplet Harvesting and Hydroamination Catalysis
Source: Inorg Chem. 2025 Feb 11;64(7):3392–402. doi: 10.1021/acs.inorgchem.4c04964 (PMC12164261; doi:10.1021/acs.inorgchem.4c04964)
Supplement: Supplementary file 1 [file ic4c04964_si_001.pdf]

Supporting Information

Gold(I) Complexes with Bulky Phosphanes: A Dual Approach to Triplet Harvesting and Hydroamination Catalysis

Araceli de Aquino,[a] Nazaret Santamaría,[b] Artur J. Moro,[c] David Aguilà,[a] Auxiliadora Prieto,[b] M. Carmen Nicasio,[b,*] João Carlos Lima,[c,*] Laura Rodríguez[a,*]

[a] *Departament de Química Inorgànica i Orgànica. Secció de Química Inorgànica. Universitat de Barcelona, and Institut de Nanociència i Nanotecnologia (IN2UB). Martí i Franquès 1-11, 08028 Barcelona (Spain). e-mail: laurarodriguezr@ub.edu*

[b] *Departamento de Química Inorgánica, Universidad de Sevilla, Calle Profesor García González 1, 41012 Sevilla (Spain). e-mail: mnicasio@us.es*

[c] *LAQV-REQUIMTE, Departamento de Química, Faculdade de Ciências e Tecnologia, Universidade Nova de Lisboa, 2829-516 Caparica, (Portugal). e-mail: lima@fct.unl.pt*

Table of contents

1. NMR spectra of gold and organic compounds S2
2. X-ray structural data of gold(I) complexes S21
3. Photophysical characterization S23
4. Computational study S51
5. Tables S54
6. Catalyst screening S58
7. General catalytic procedure for hydroamination reactions S58
8. Characterization data of reaction products S59

1. NMR spectra of gold compounds.

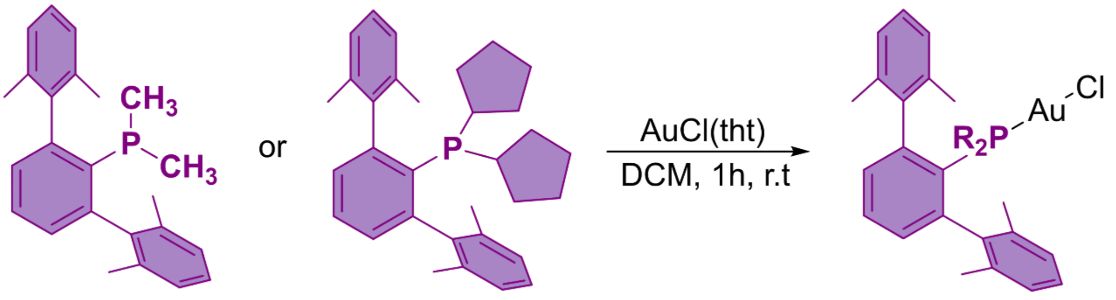

P1 **P2** **AuClPx**

R = Me; for **AuClP1**
R = Cyp; for **AuClP2**

Scheme S1. Synthesis of Phosphane-Au-Cl derivatives used in this work.

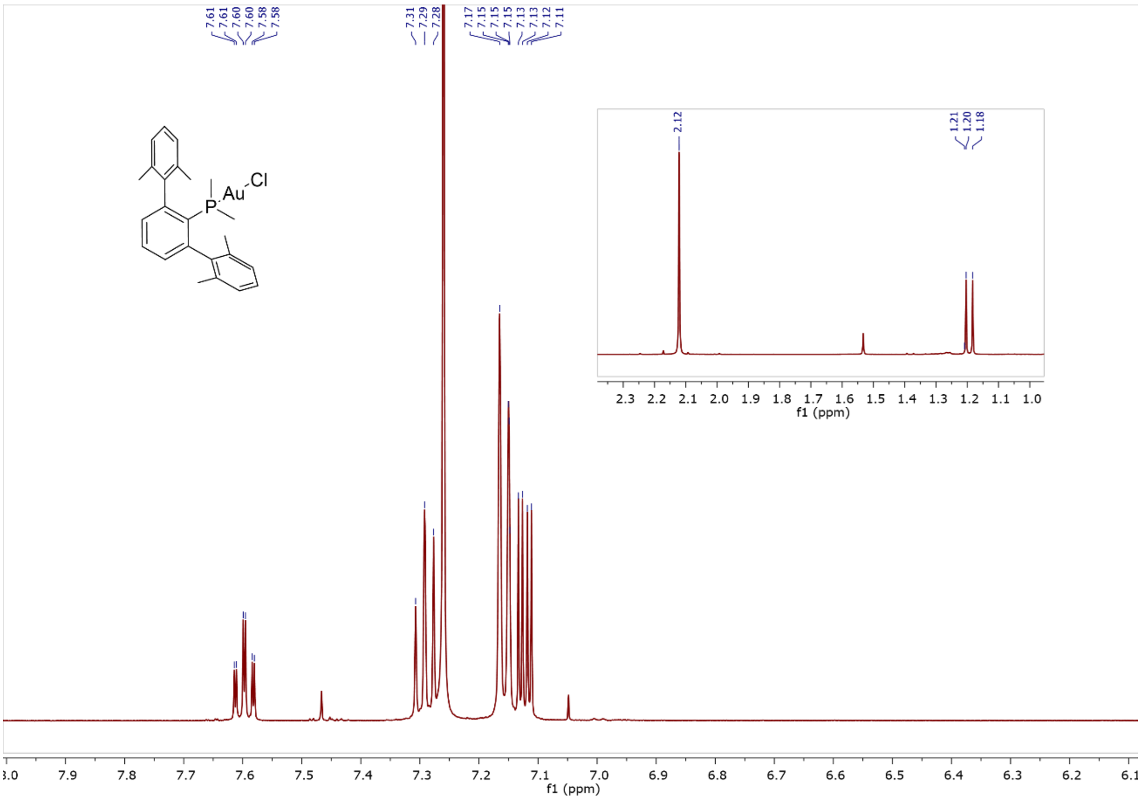

Figure S1. ^{1}H NMR spectrum of **AuClP1** (400 MHz, $CDCl_3$, 25 ºC).

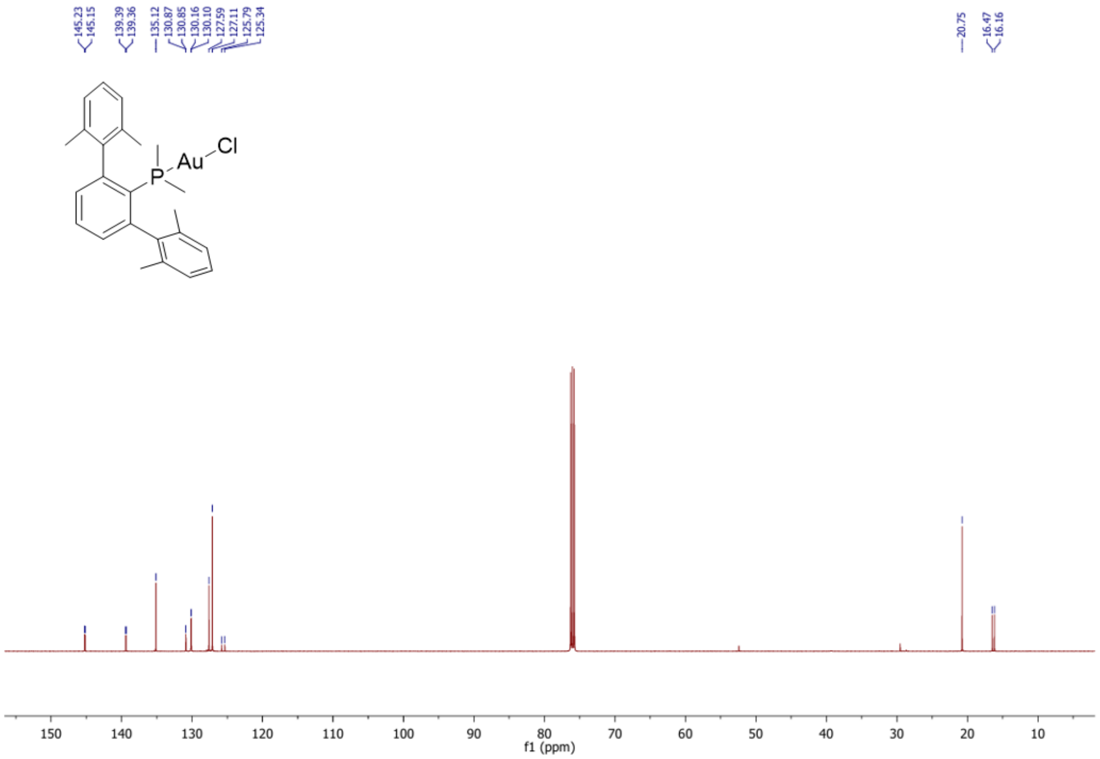

Figure S2. $^{13}C\{^{1}H\}$ NMR spectrum of **1** (125 MHz, $CDCl_3$, 25 ºC).

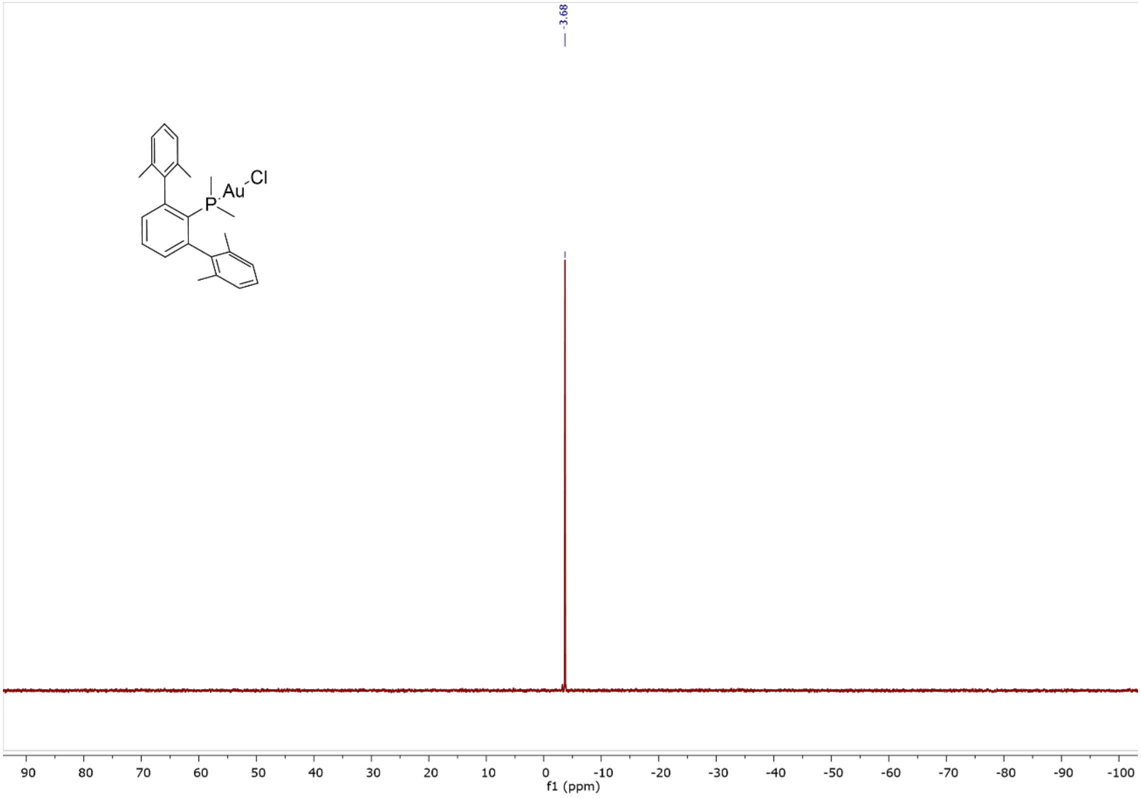

Figure S3. $^{31}P\{^{1}H\}$ NMR spectrum of **1** (161.9 MHz, $CDCl_3$, 25 ºC).

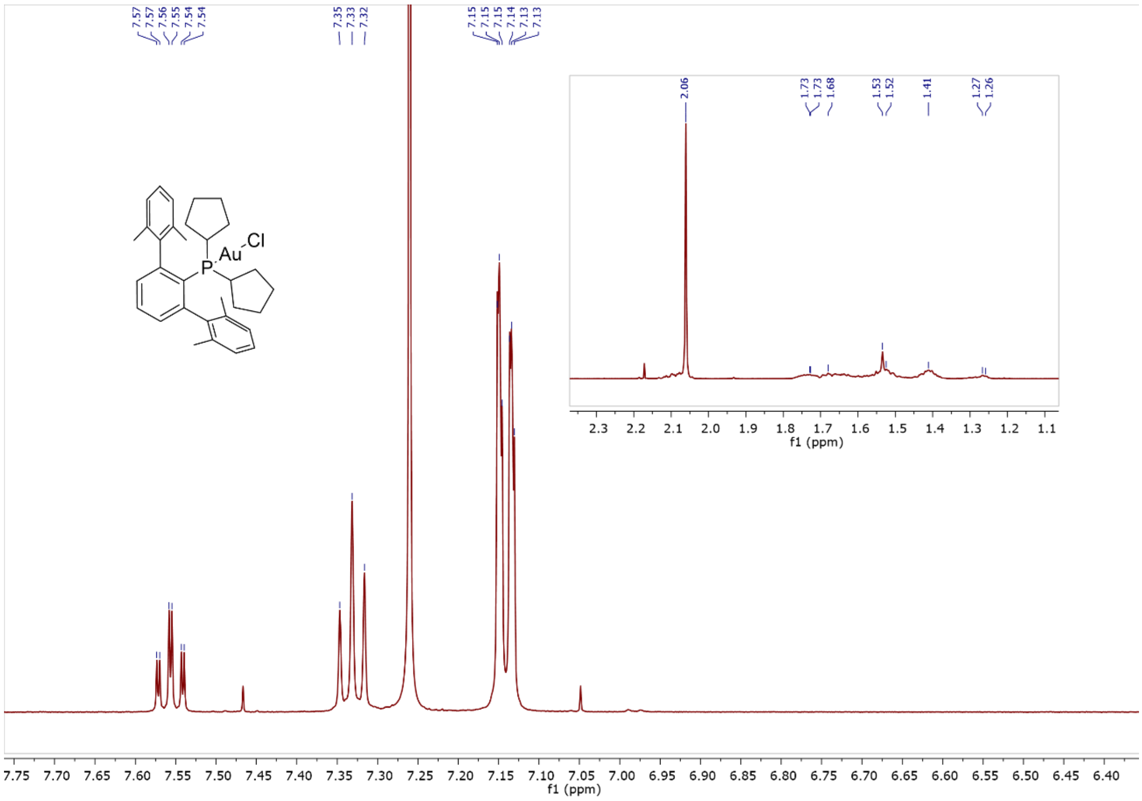

Figure S4. ^{1}H NMR spectrum of **2 (**400 MHz, $CDCl_3$, 25 ºC).

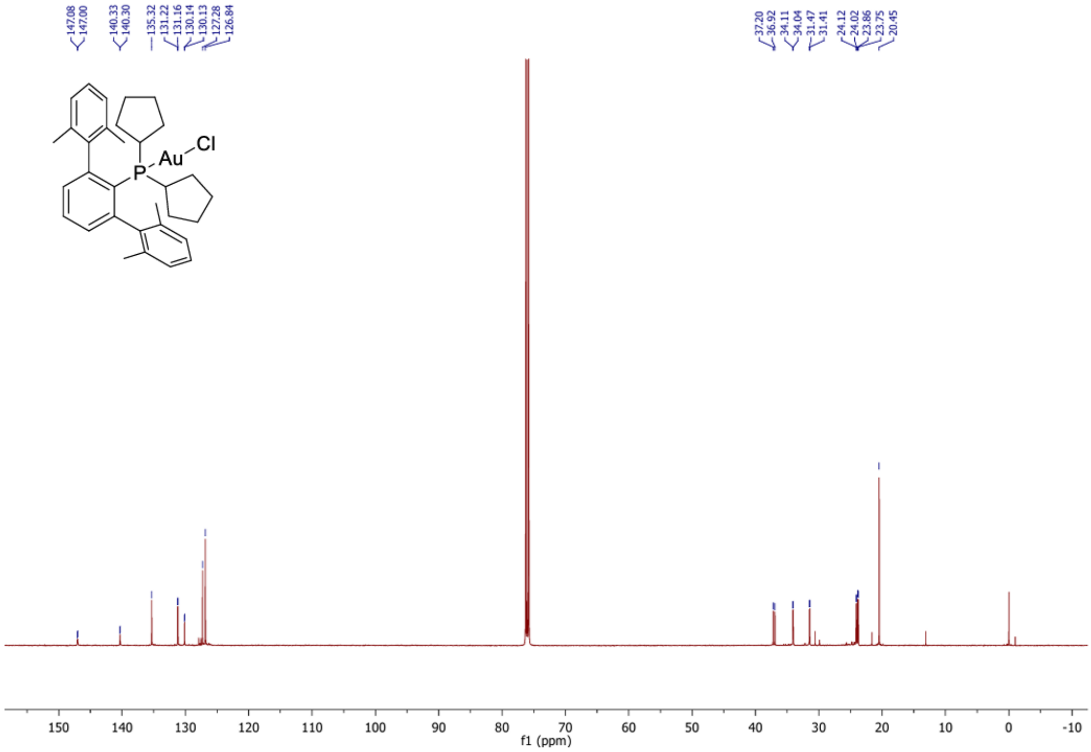

Figure S5. $^{13}C\{^{1}H\}$ NMR spectrum of **2** (125 MHz, $CDCl_3$, 25 ºC).

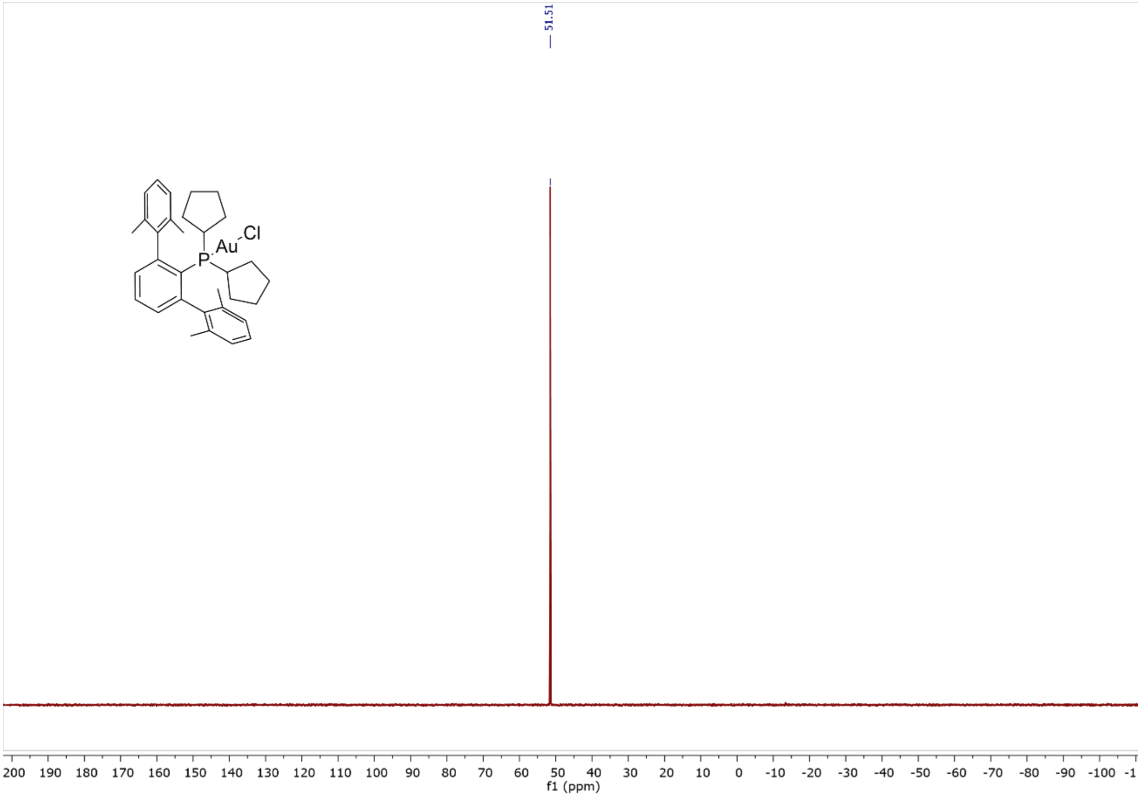

Figure S6. $^{31}P\{^1H\}$ NMR spectrum of **2** (161.9 MHz, $CDCl_3$, 25 ºC).

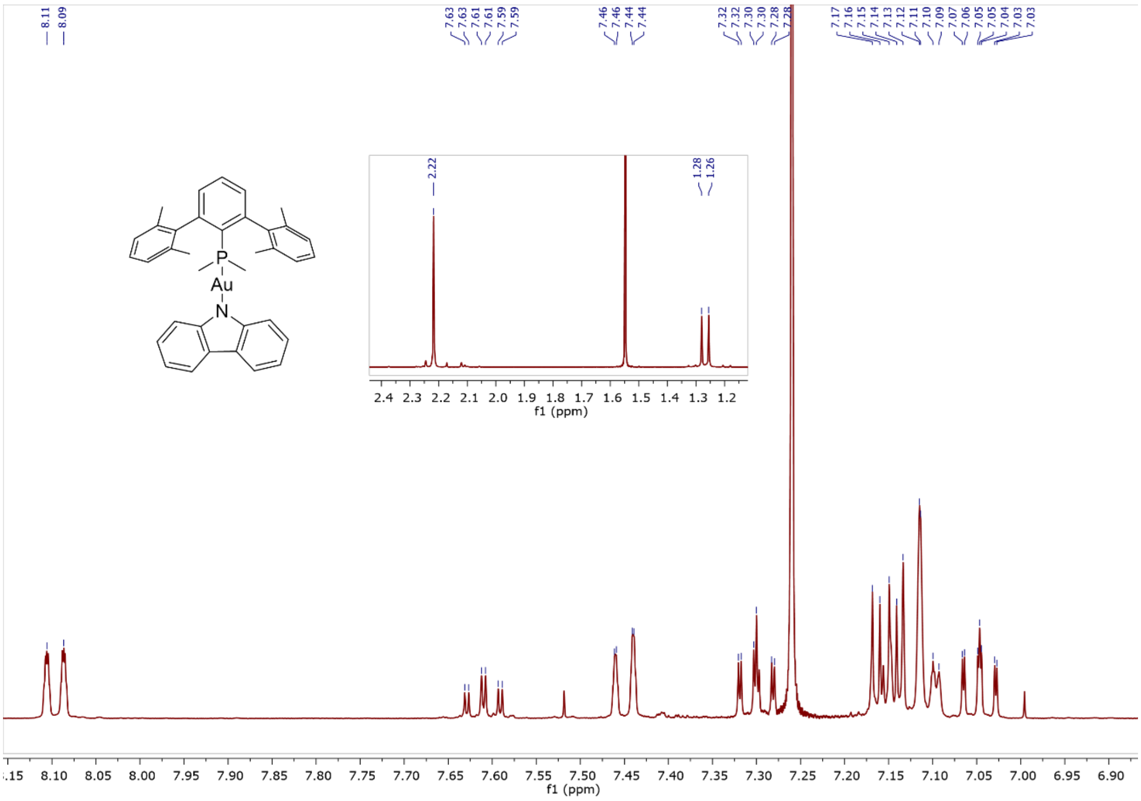

Figure S7. ^1H NMR spectrum of **1a** (400 MHz, $CDCl_3$, 25 ºC).

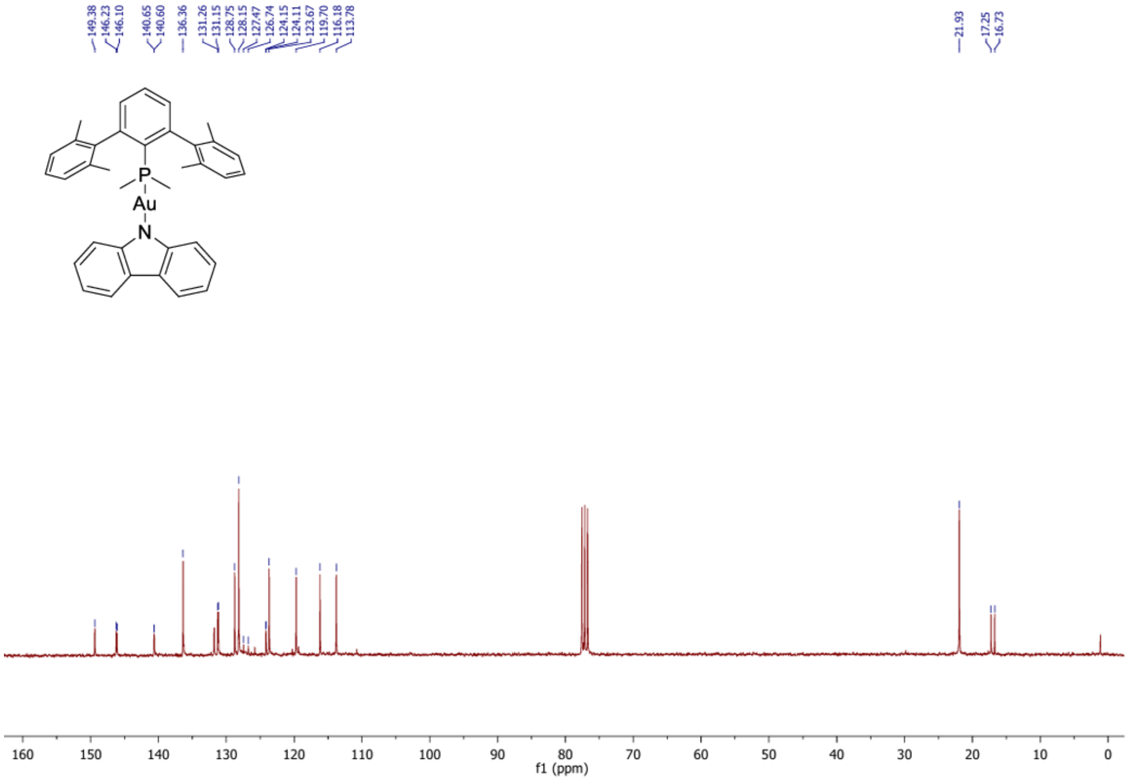

Figure S8. $^{13}C\{^{1}H\}$ NMR spectrum of **1a** (75 MHz, $CDCl_3$, 25 ºC).

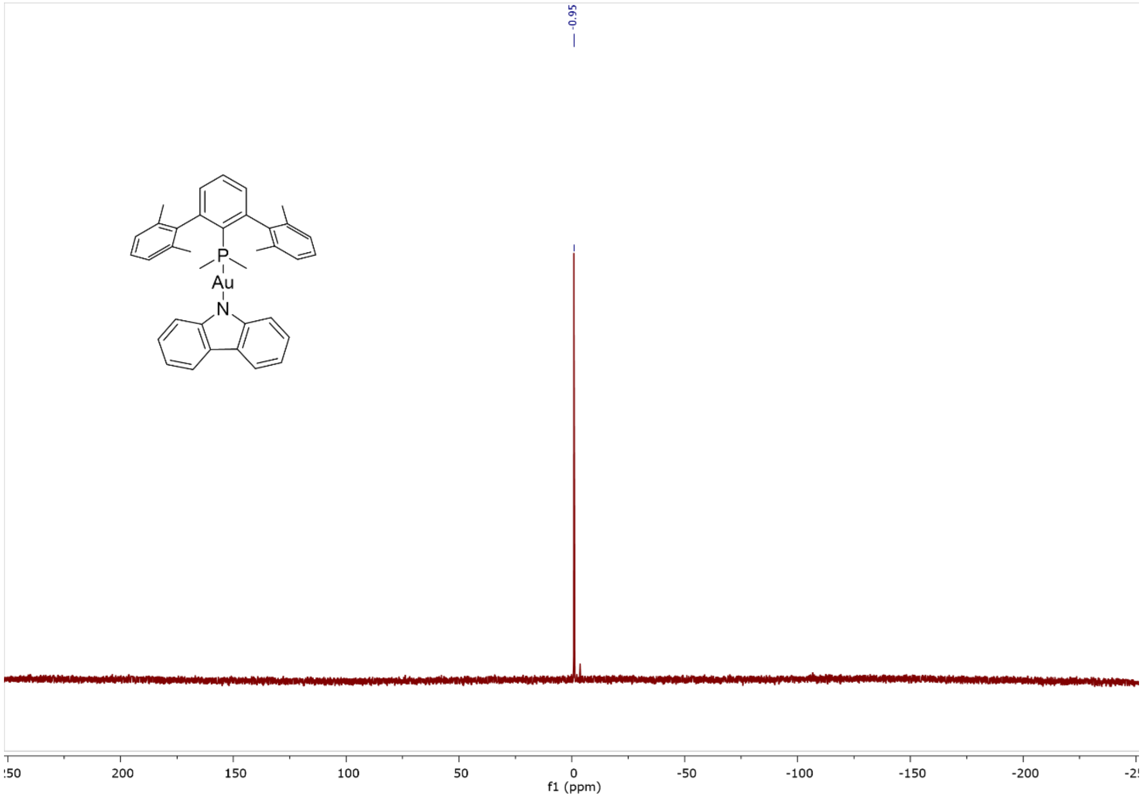

Figure S9. $^{31}P\{^{1}H\}$ NMR spectrum of **1a** (161.9 MHz, $CDCl_3$, 25 ºC).

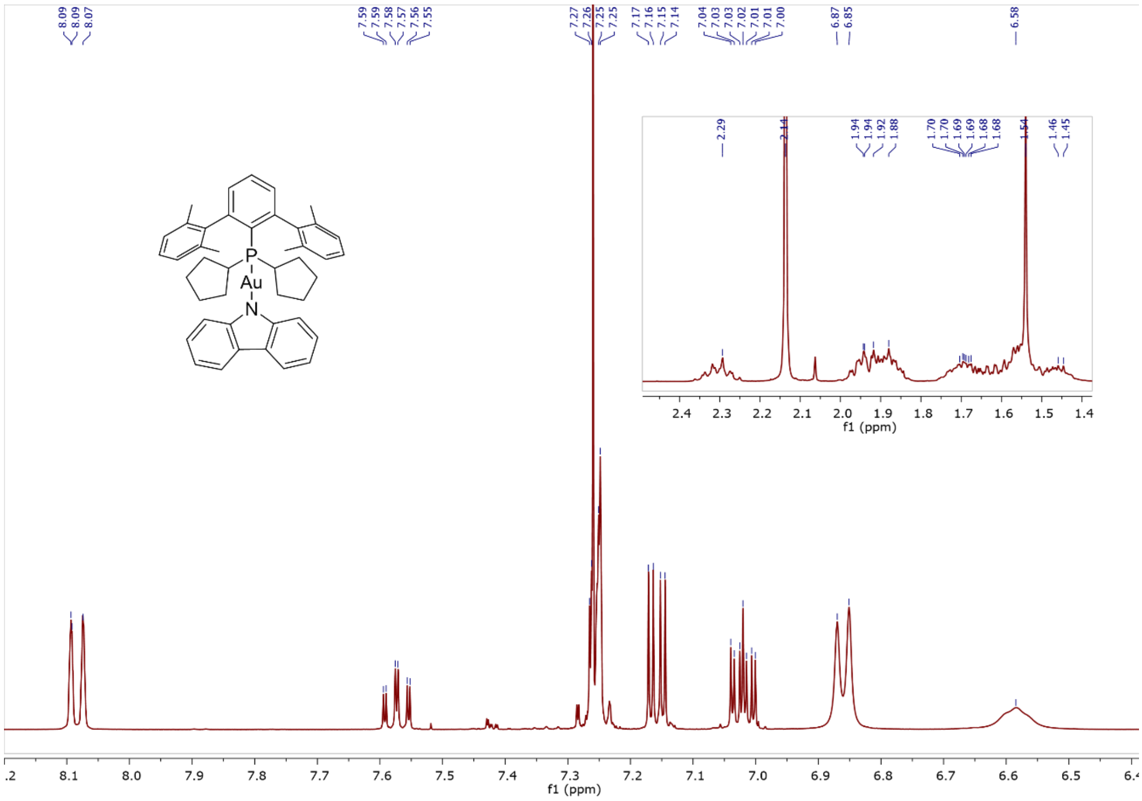

Figure S10. ^{1}H NMR spectrum of **2a** (400 MHz, $CDCl_3$, 25 ºC).

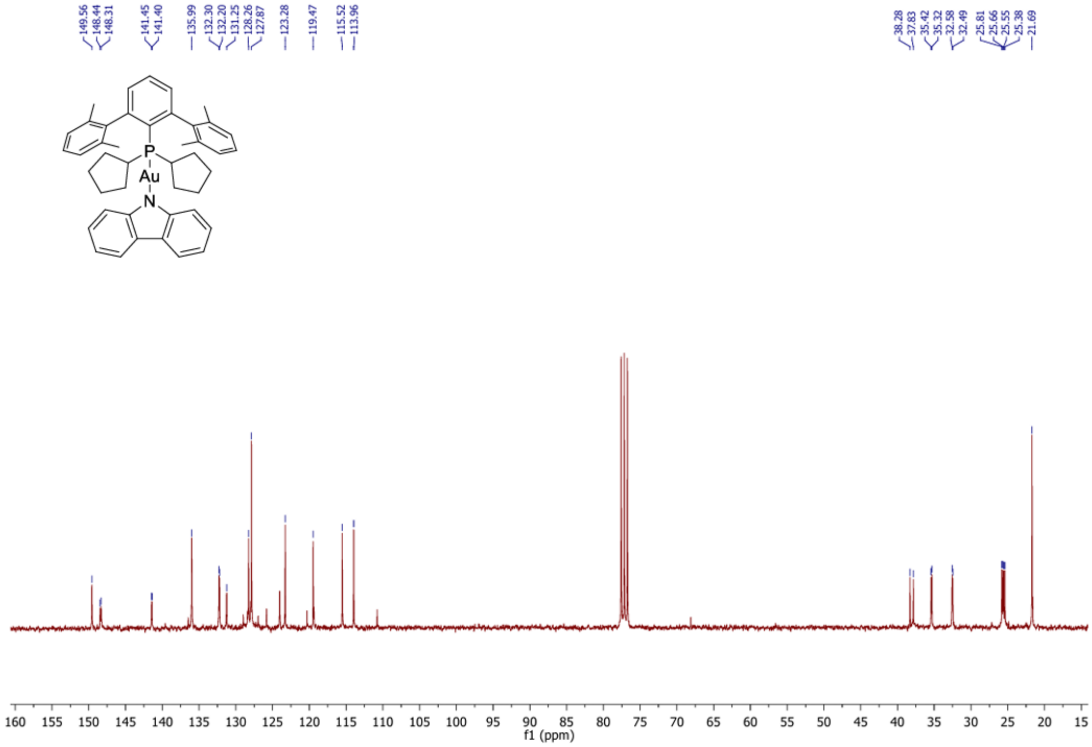

Figure S11. $^{13}C\{^{1}H\}$ NMR spectrum of **2a** (75 MHz, $CDCl_3$, 25 ºC).

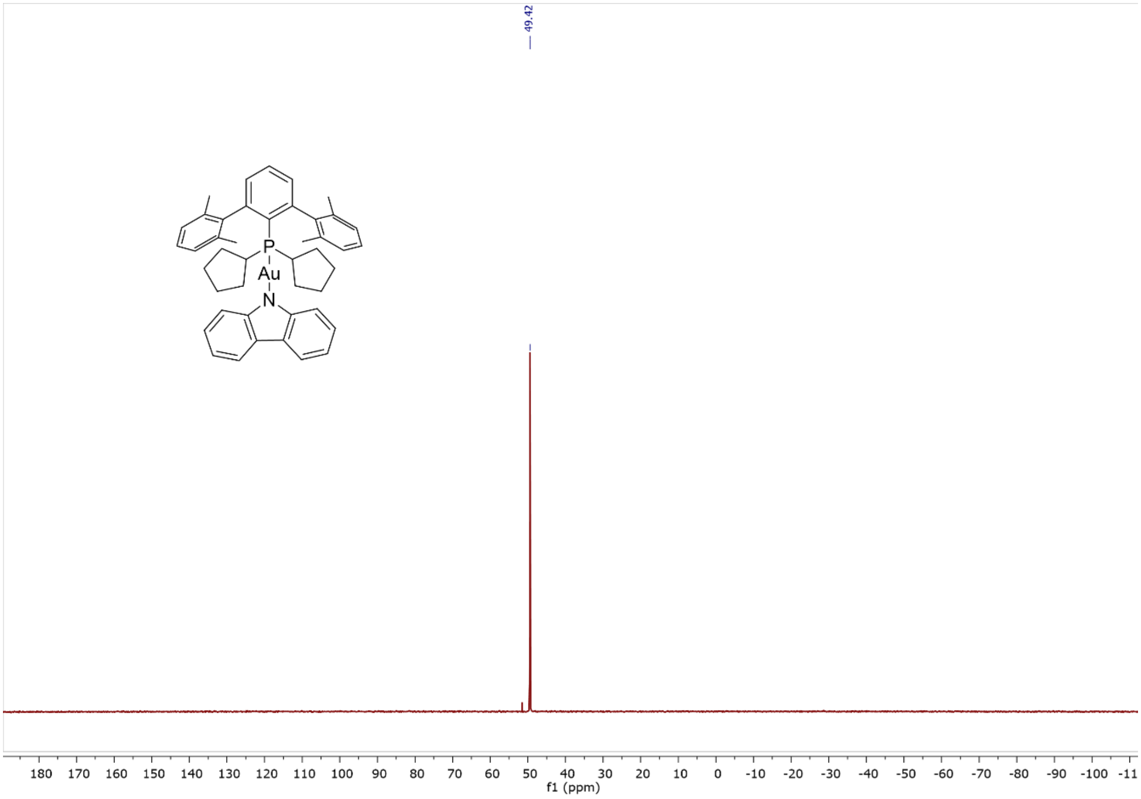

Figure S12. $^{31}P\{^{1}H\}$ NMR spectrum of **2a** (161.9 MHz, $CDCl_3$, 25 ºC).

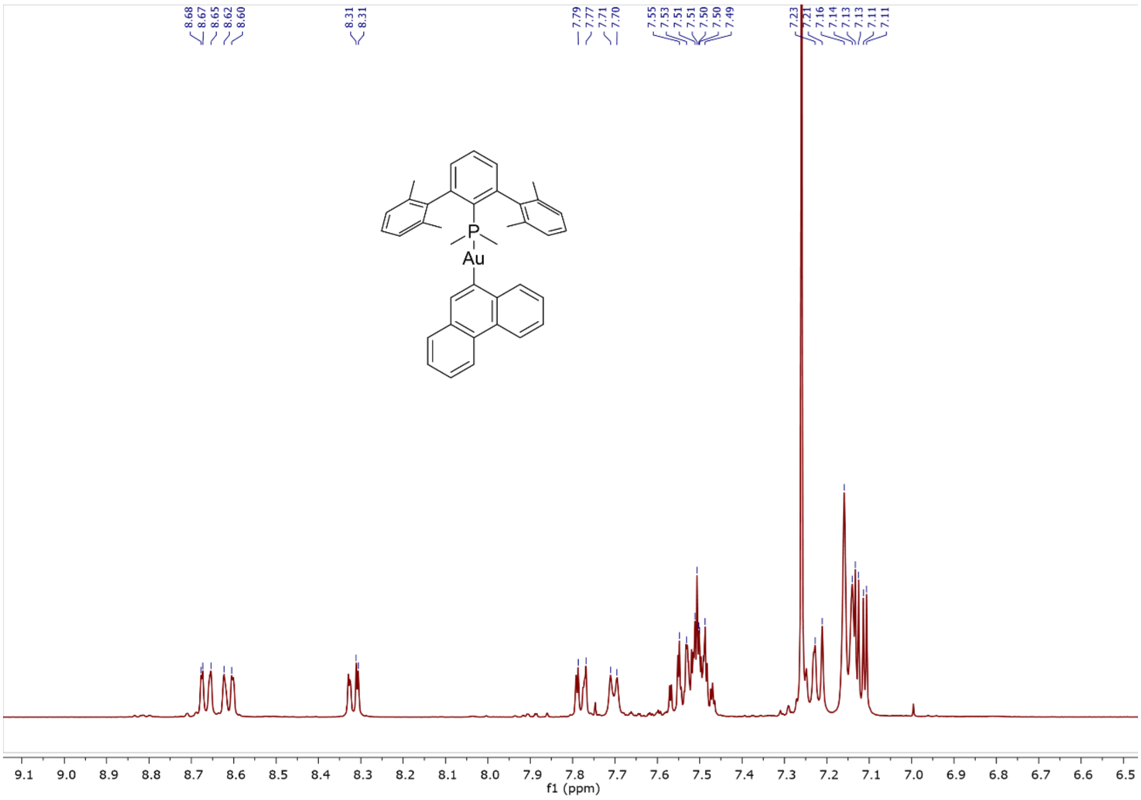

Figure S13. ^{1}H NMR spectrum of **1b** (400 MHz, $CDCl_3$, 25 ºC).

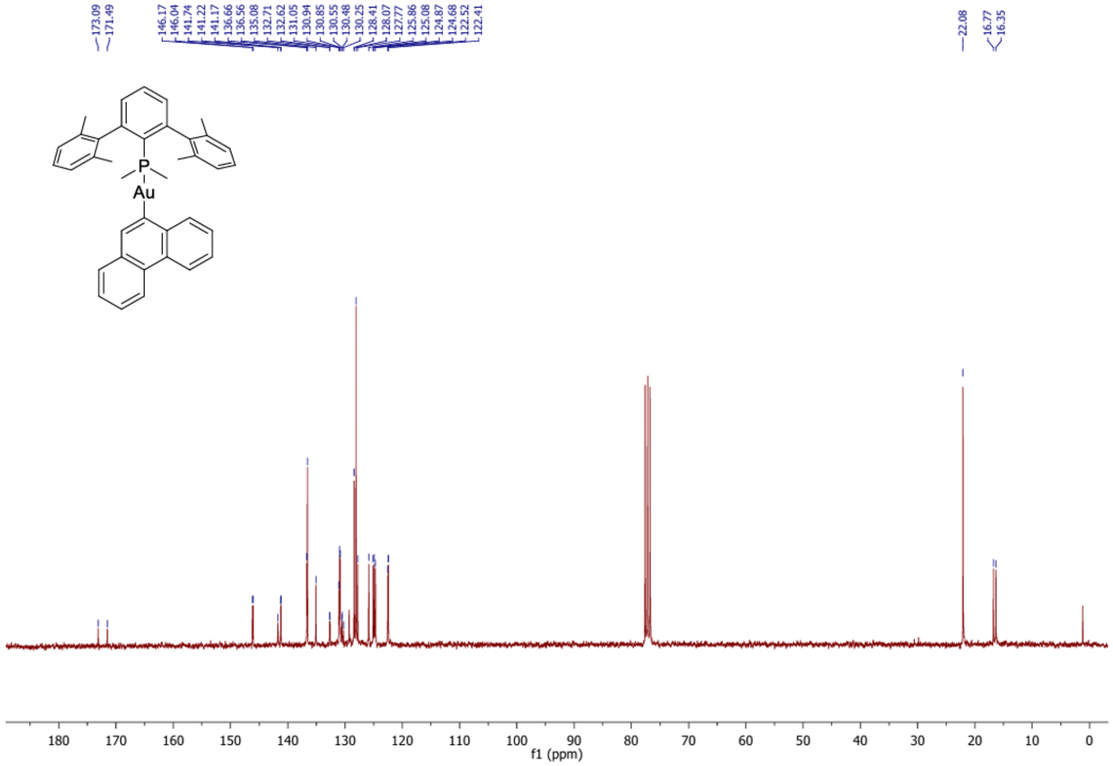

Figure S14. $^{13}C\{^{1}H\}$ NMR spectrum of **1b** (75 MHz, $CDCl_3$, 25 ºC).

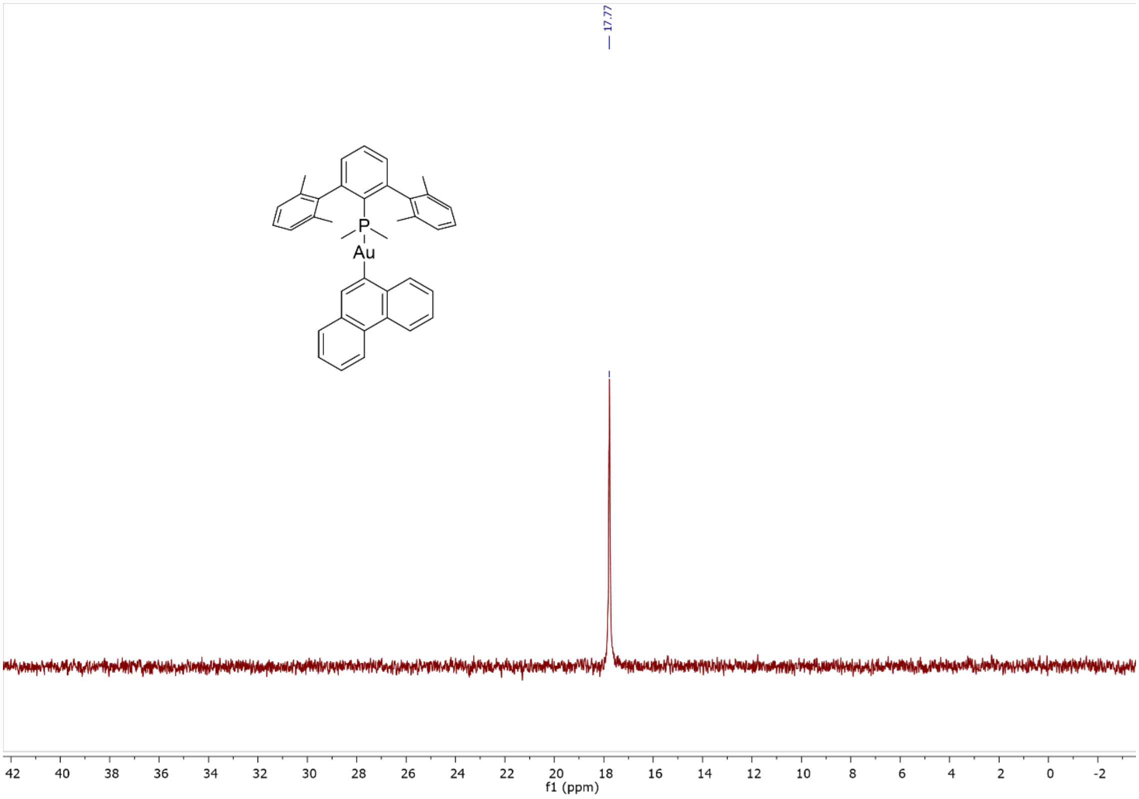

Figure S15. $^{31}P\{^{1}H\}$ NMR spectrum of **1b** (161.9 MHz, $CDCl_3$, 25 ºC).

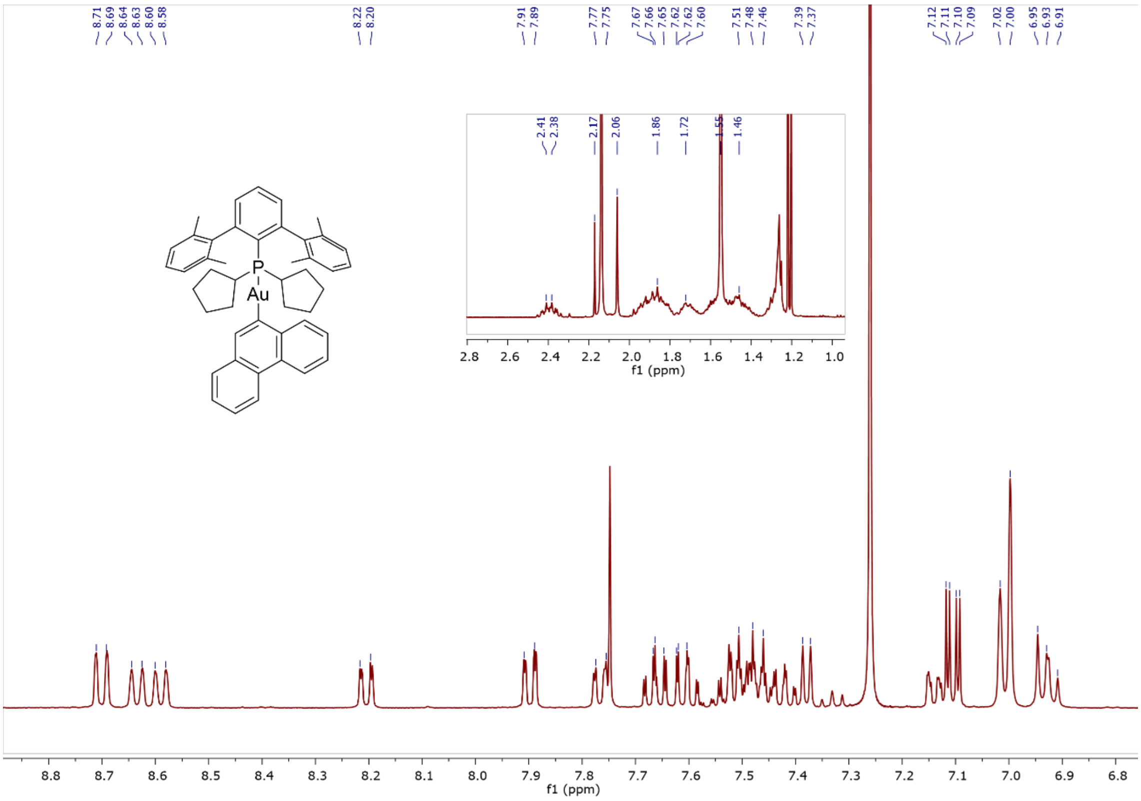

Figure S16. ^{1}H NMR spectrum of **2b** (400 MHz, $CDCl_3$, 25 ºC).

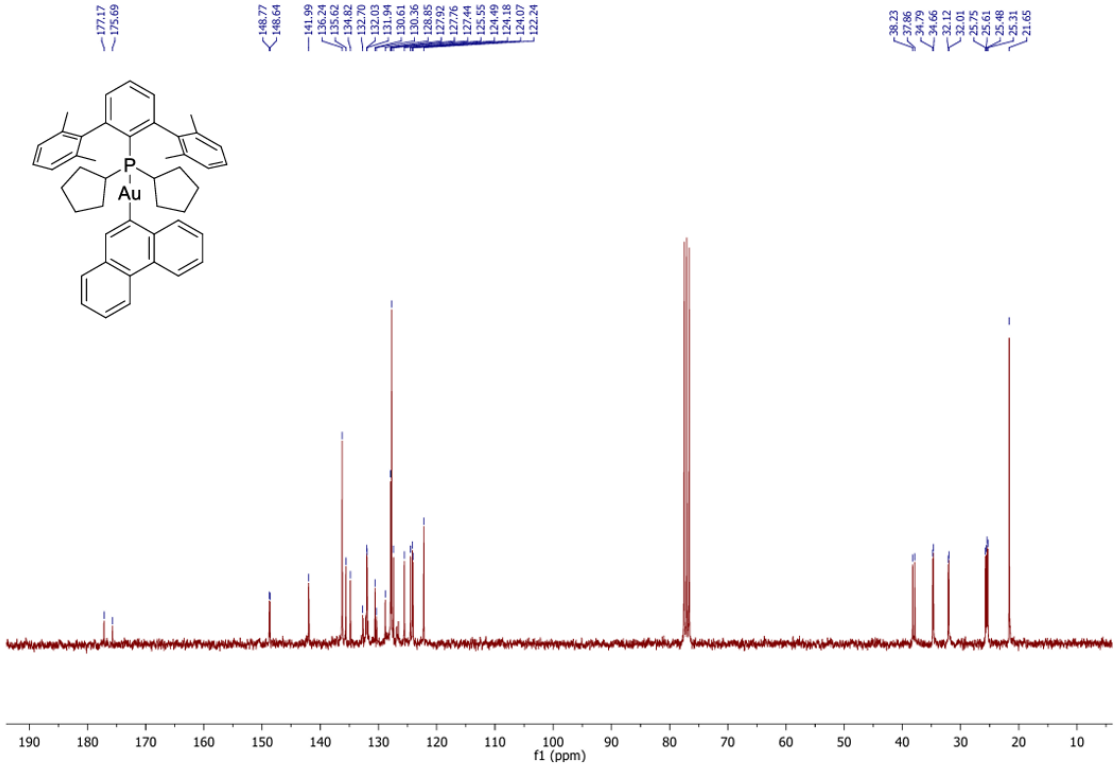

Figure S17. $^{13}C\{^{1}H\}$ NMR spectrum of **2b** (75 MHz, $CDCl_3$, 25 ºC).

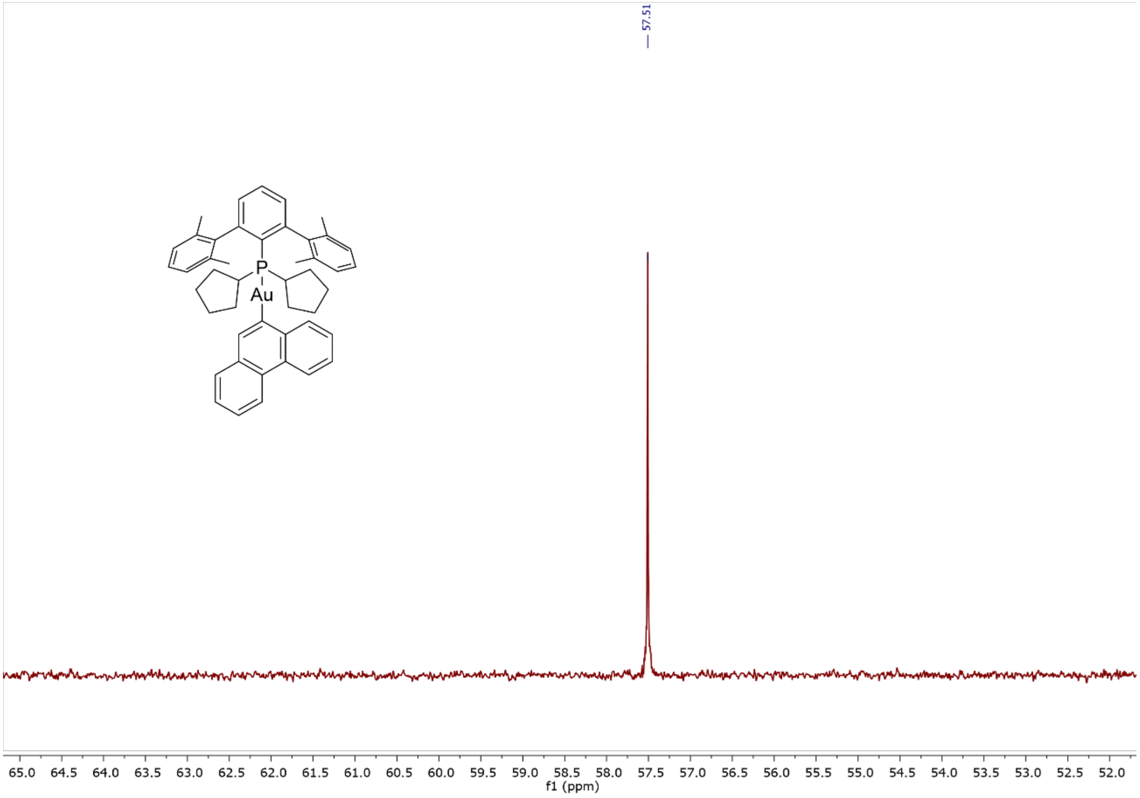

Figure S18. $^{31}P\{^{1}H\}$ NMR spectrum of **2b** (161.9 MHz, $CDCl_3$, 25 ºC).

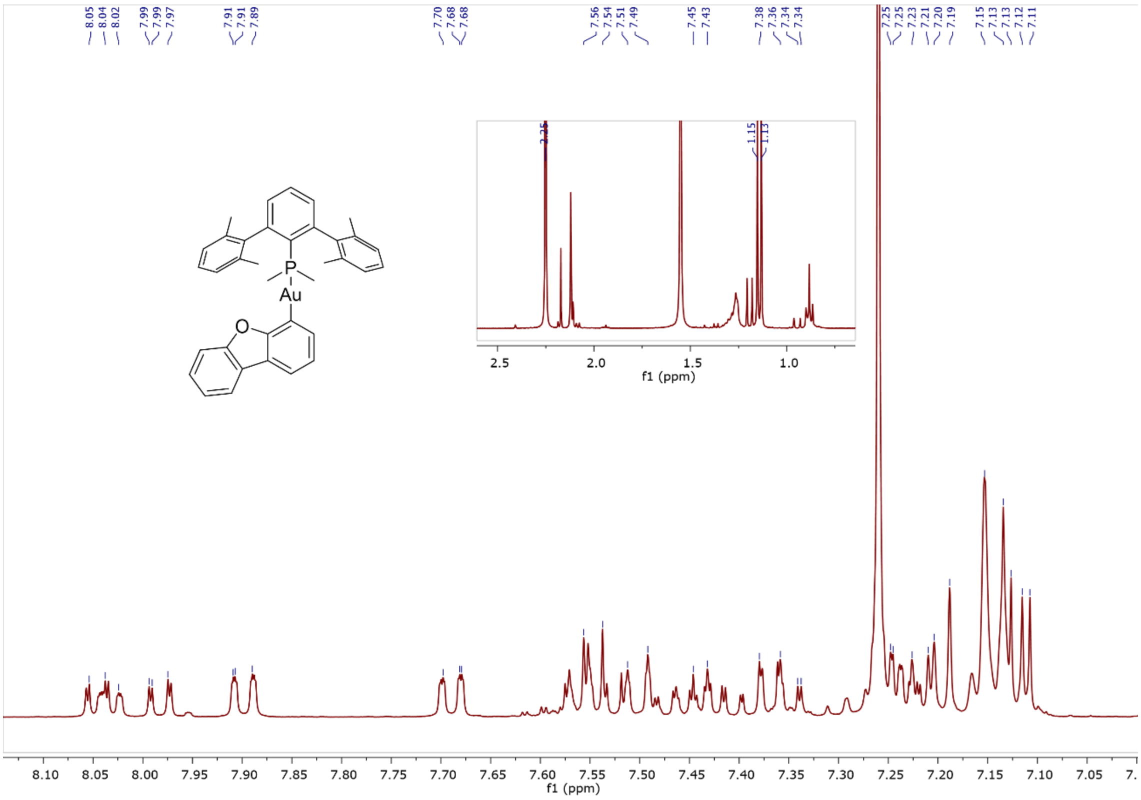

Figure S19. ^{1}H NMR spectrum of **1c** (400 MHz, $CDCl_3$, 25 ºC).

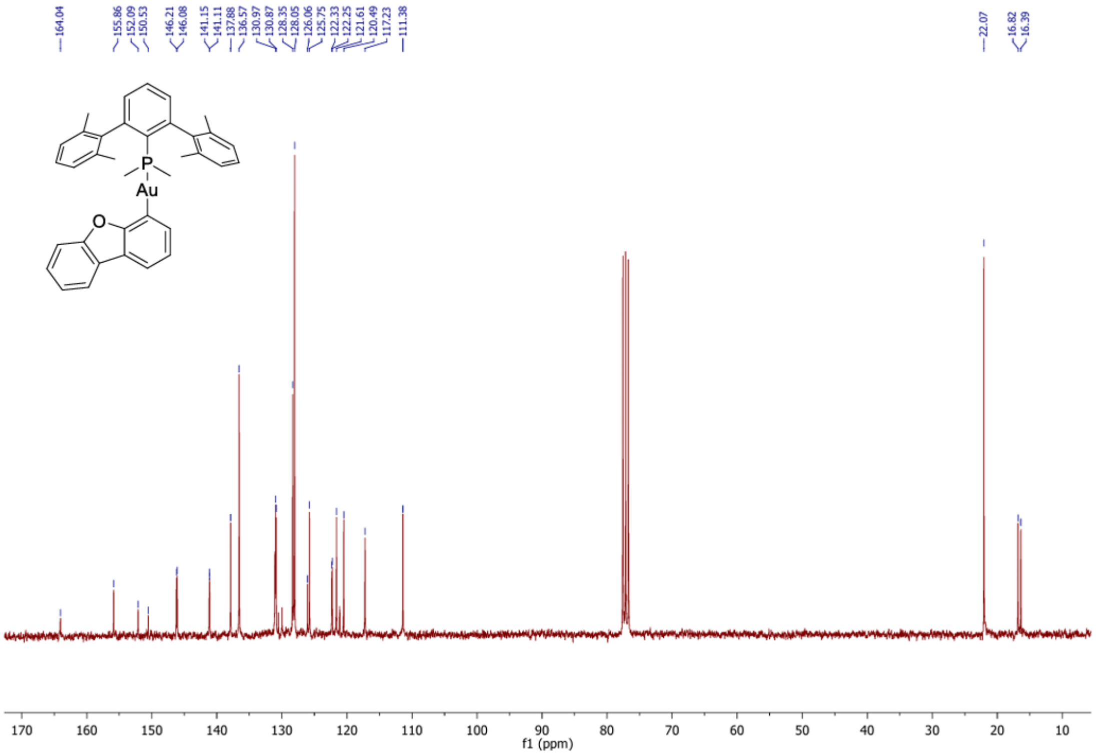

Figure S20. $^{13}C\{^{1}H\}$ NMR spectrum of **1c** (75 MHz, $CDCl_3$, 25 ºC).

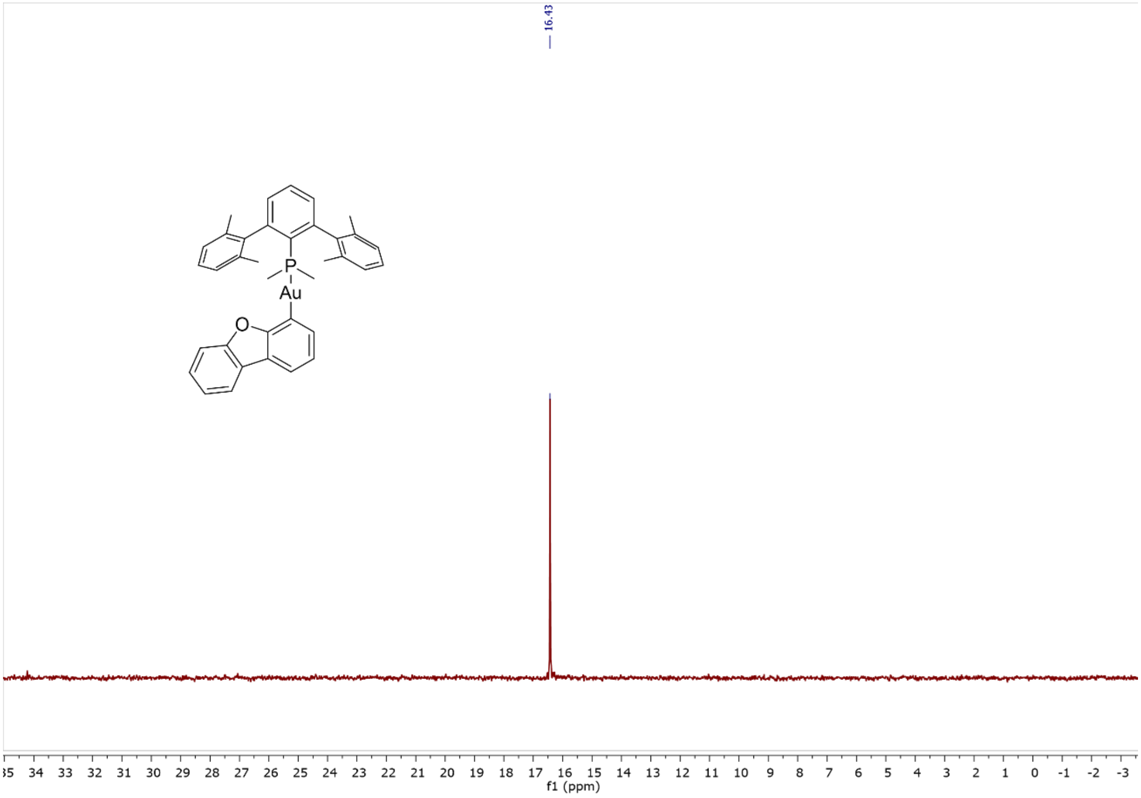

Figure S21. $^{31}P\{^{1}H\}$ NMR spectrum of **1c** (161.9 MHz, $CDCl_3$, 25 ºC).

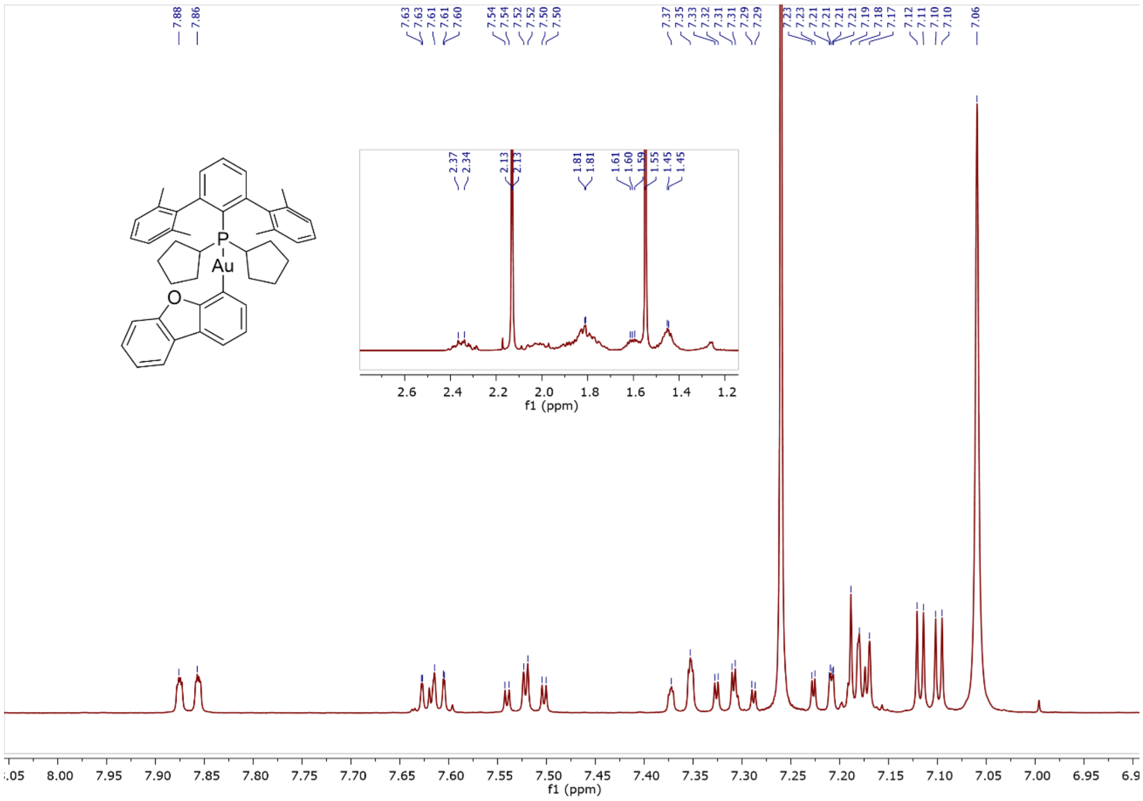

Figure S22. ^{1}H NMR spectrum of **2c** (400 MHz, $CDCl_3$, 25 ºC).

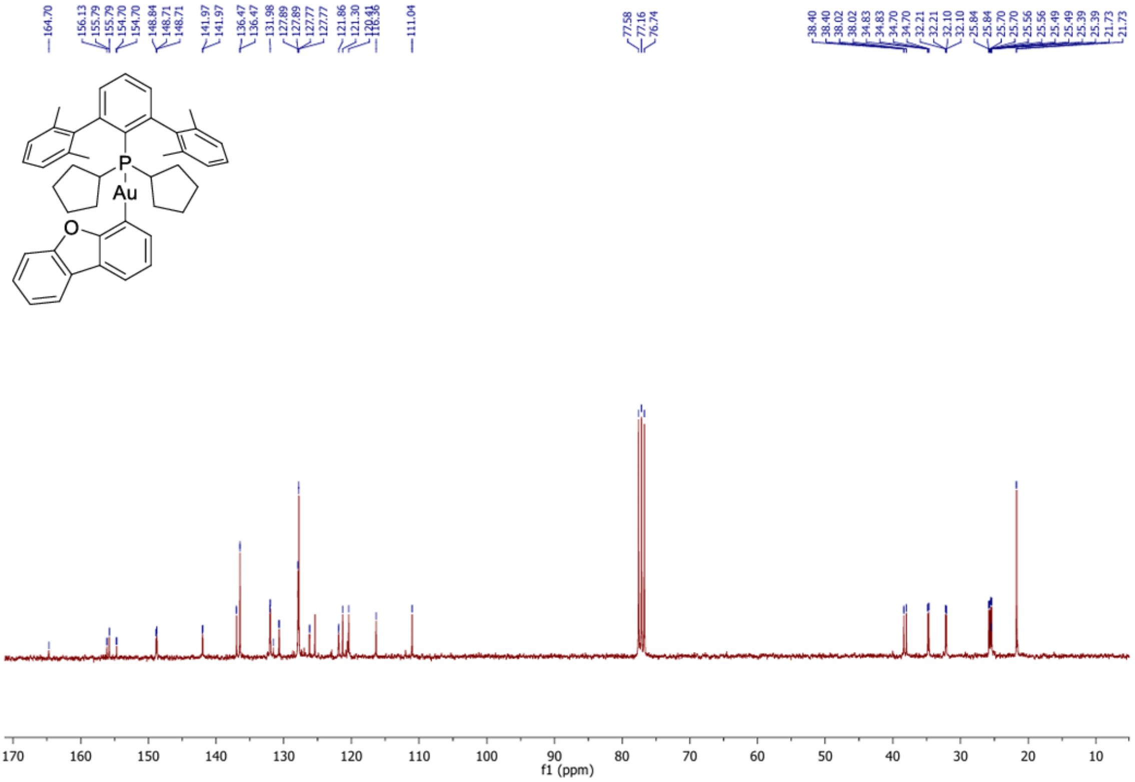

Figure S23. $^{13}C\{^{1}H\}$ NMR spectrum of **2c** (75 MHz, $CDCl_3$, 25 ºC).

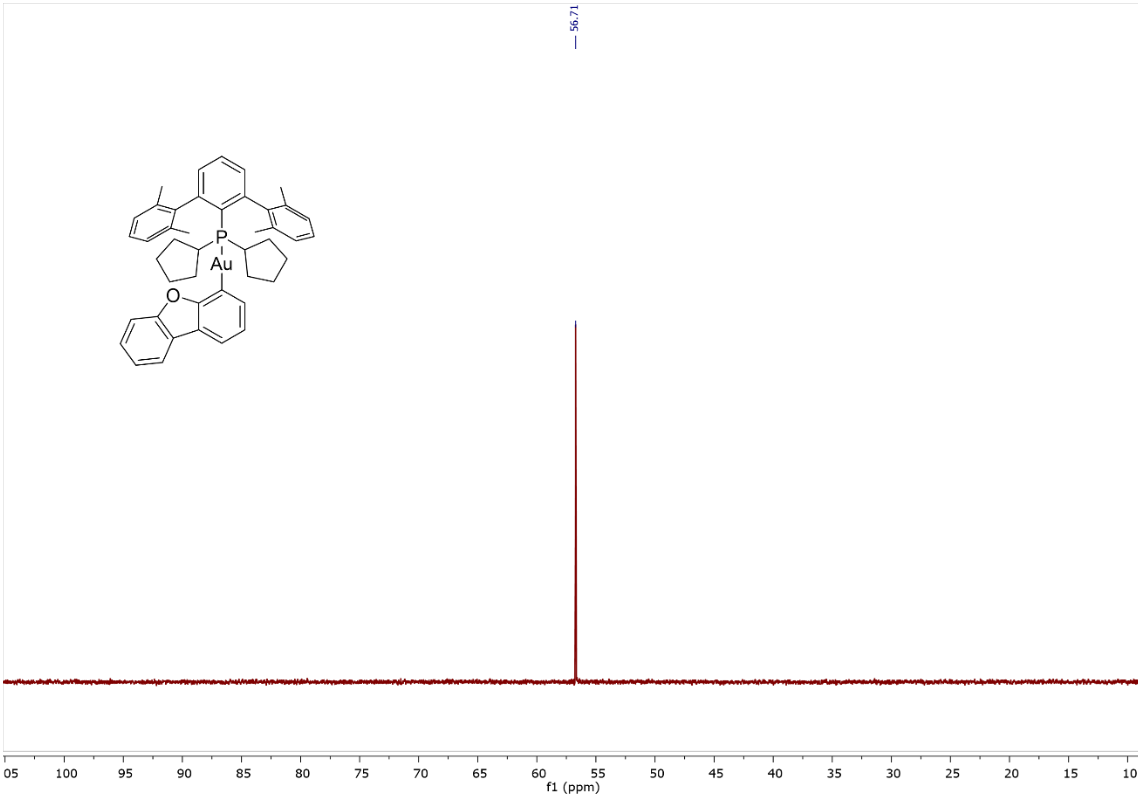

Figure S24. $^{31}P\{^1H\}$ NMR spectrum of **2c** (161.9 MHz, $CDCl_3$, 25 ºC).

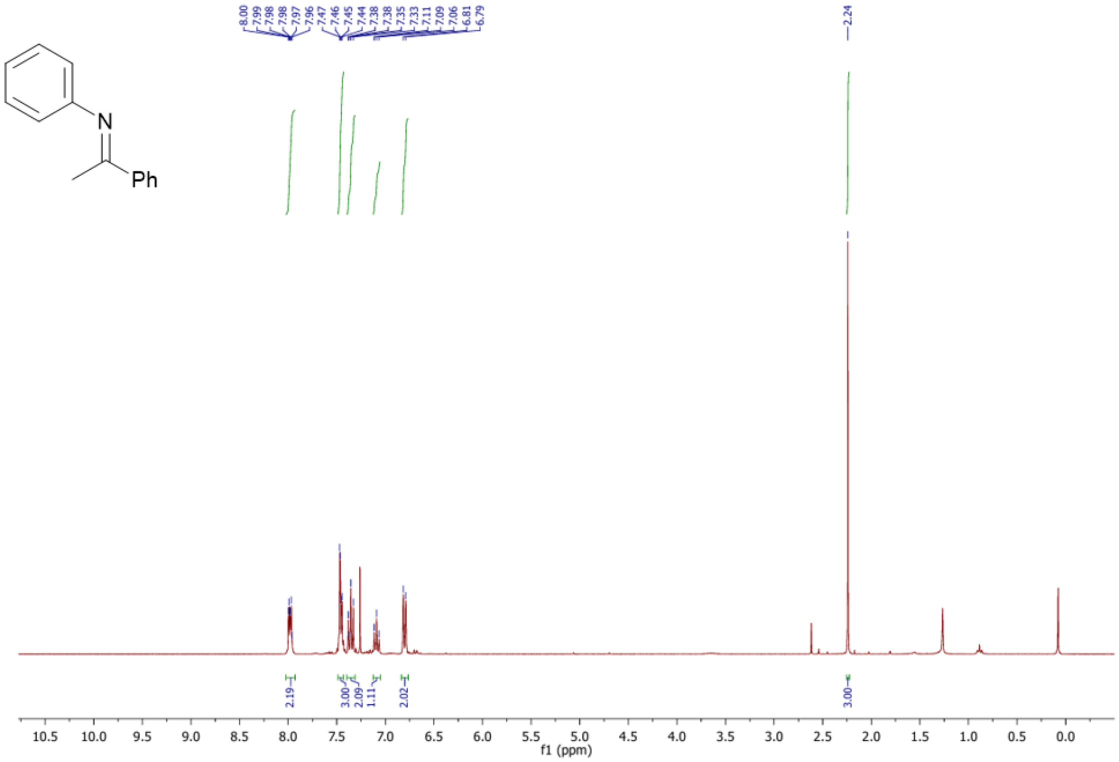

Figure S25. ^{1}H NMR spectrum of (*E*)-*N*-diphenylethan-1-imine (Scheme 3, **aa**).

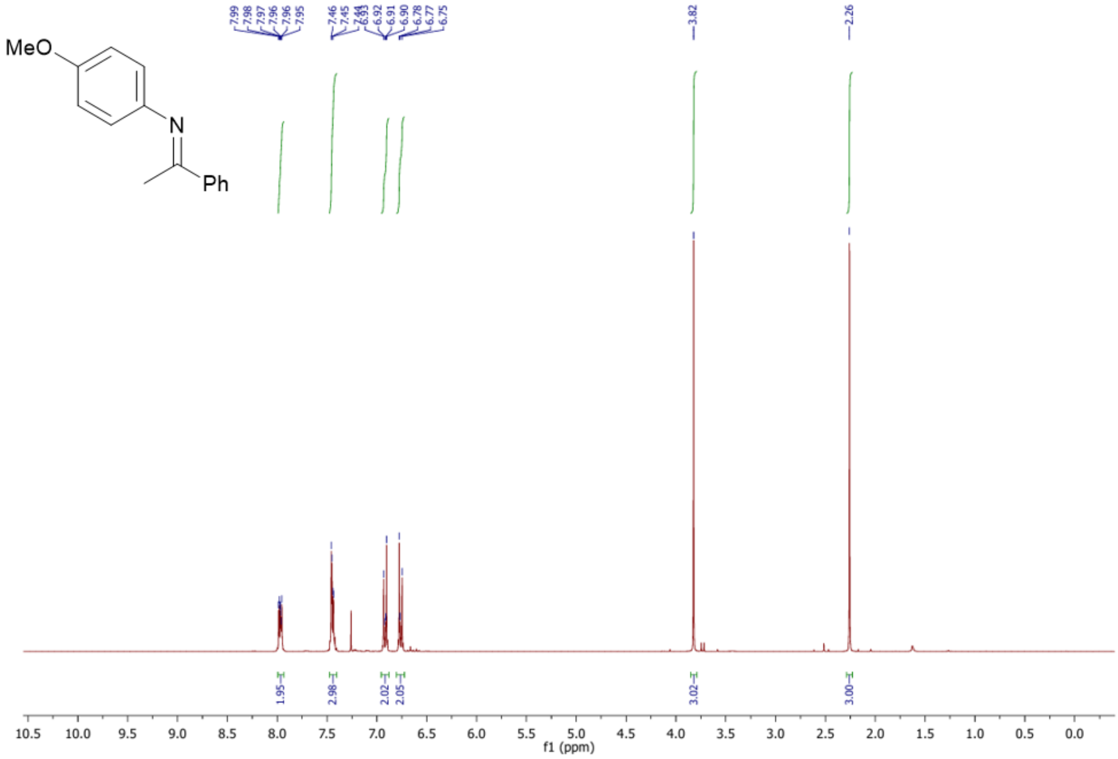

Figure S26. ¹H NMR spectrum of (*E*)-*N*-(4-Methoxyphenyl)-1-phenylethan-1-imine (Scheme 3, **ab**).

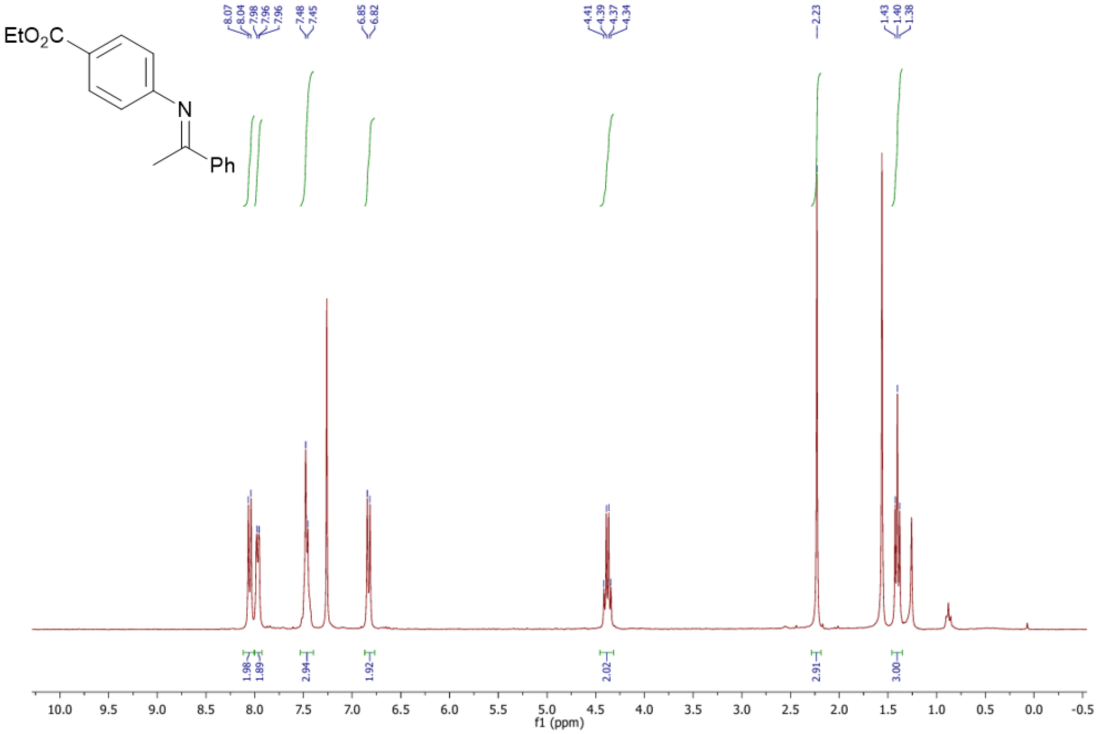

Figure S27. ¹H NMR spectrum of Ethyl (*E*)-4-((1-phenylethylidene)amino)benzoate (Scheme 3, **ac**).

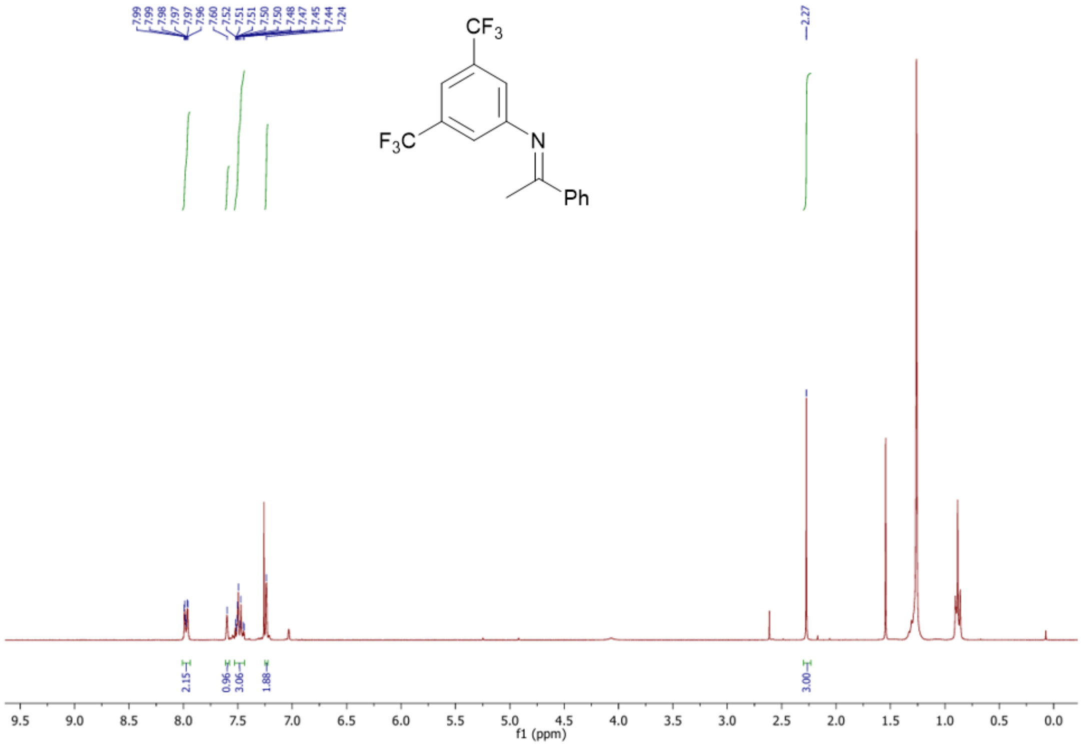

Figure S28. ^{1}H NMR spectrum of (*E*)-*N*-(3,5-bis(trifluoromethyl)phenyl)-1-phenylethan-1-imine (Scheme 3, **ad**).

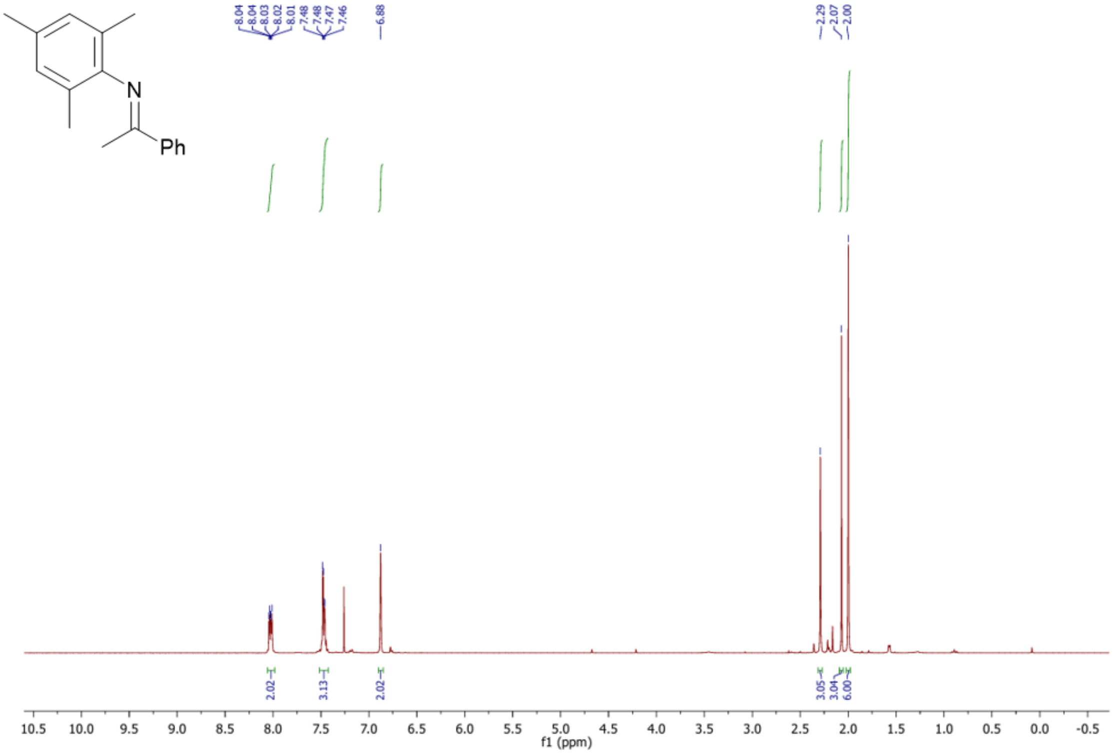

Figure S29. ^{1}H NMR spectrum of (*E*)-*N*-mesityl-1-phenylethan-1-imine (Scheme 3, **ae**).

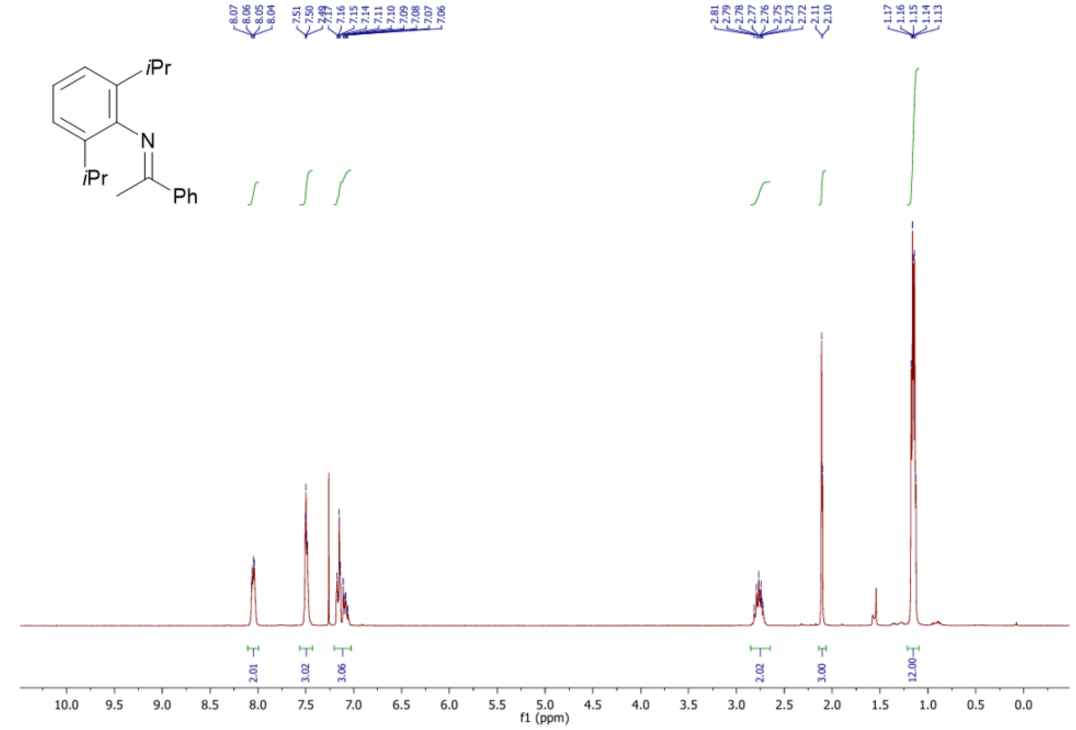

Figure S30. ^{1}H NMR spectrum of (*E*)-*N*-(2,6-diisopropylphenyl)-1-phenylethan-1-imine (Scheme 3, **af**).

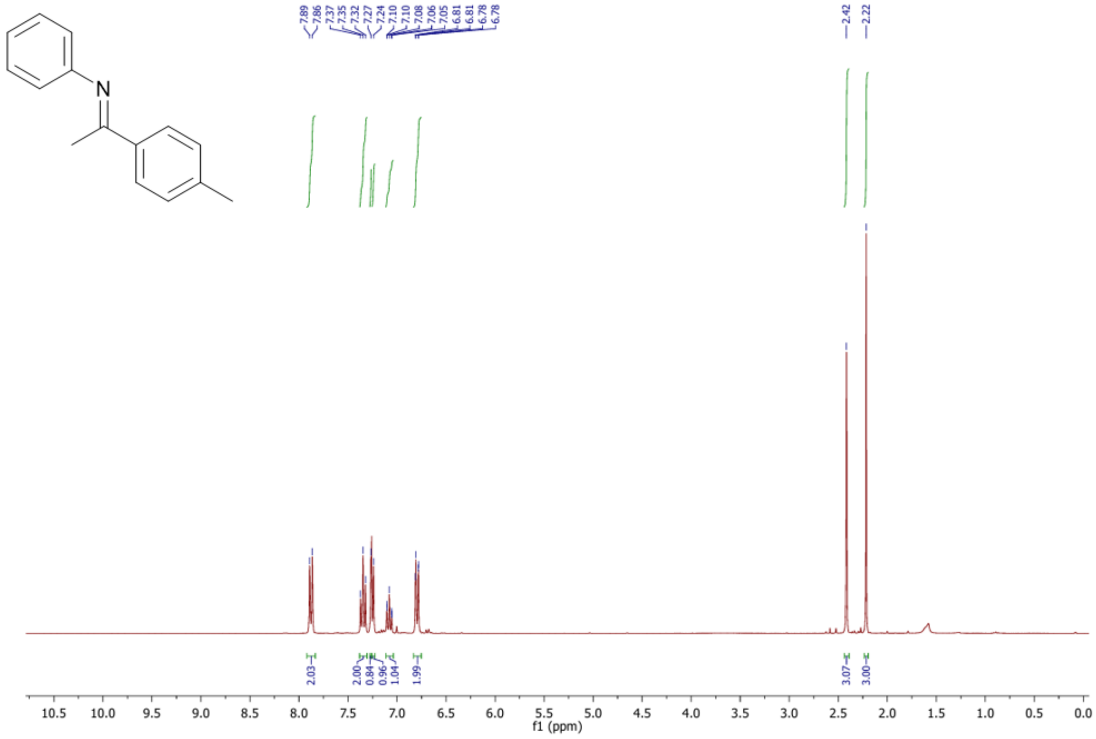

Figure S31. ^{1}H NMR spectrum of (*E*)-*N*-phenyl-1-(p-tolyl)ethan-1-imine (Scheme 3, **ag**).

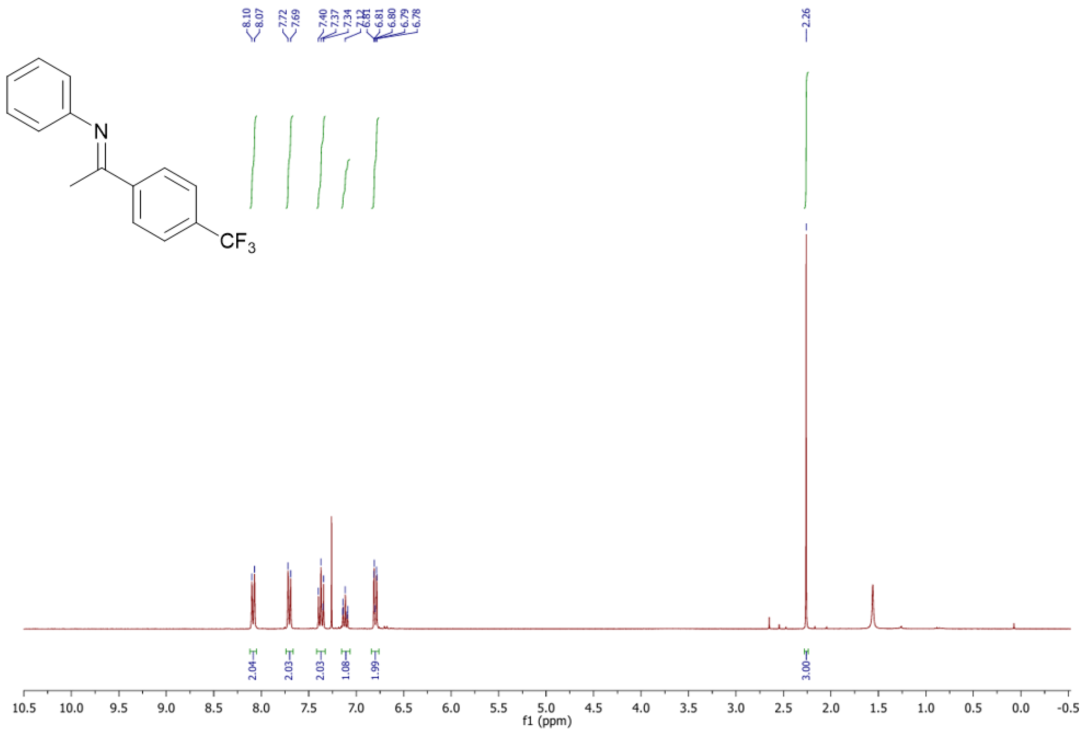

Figure S32. ^{1}H NMR spectrum of (*E*)-*N*-phenyl-1-(4-(trifluoromethyl)phenyl)ethan-1-imine (Scheme 3, **ah**).

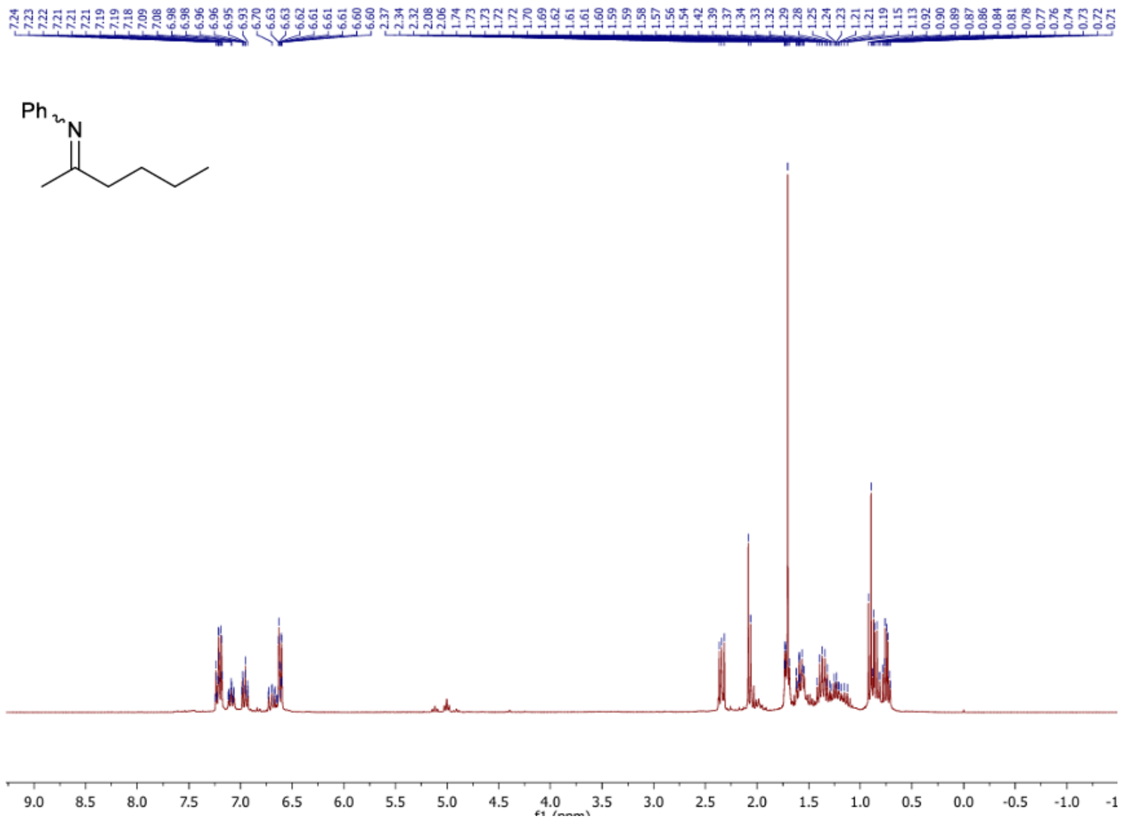

Figure S33. ^{1}H NMR spectrum of *N*-phenylhexan-2-imine (Scheme 3, **ai**).

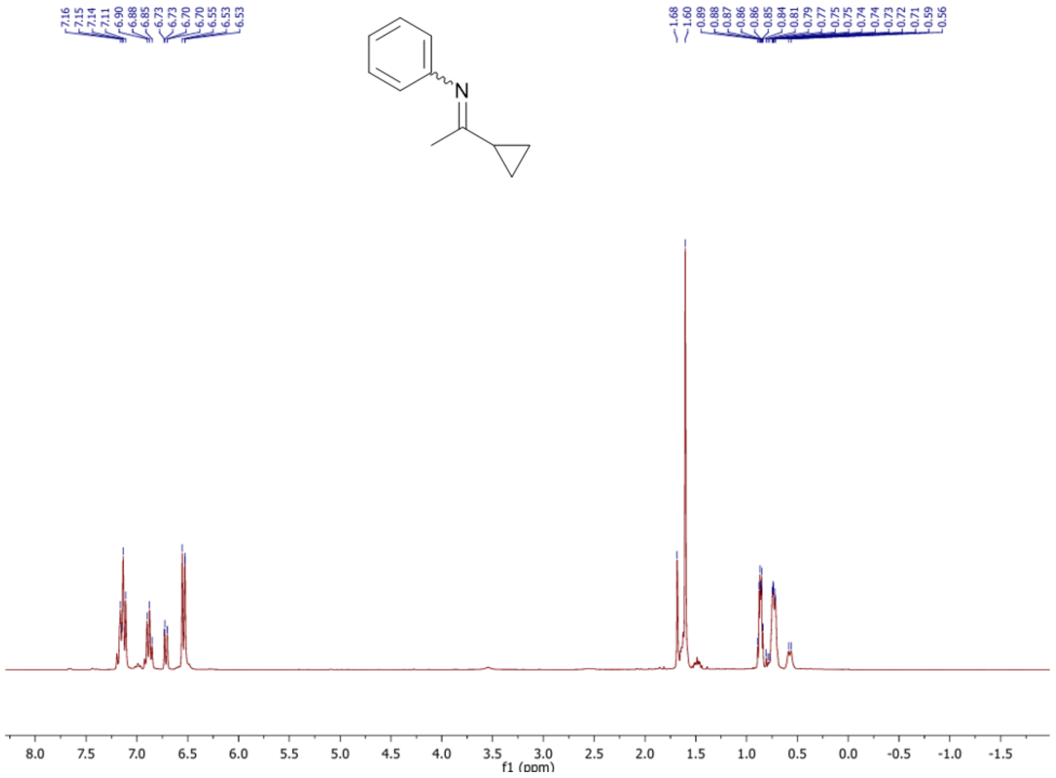

Figure S34. ^{1}H NMR spectrum of 1-cyclopropyl-*N*-phenylethan-1-imine (Scheme 3, **aj**).

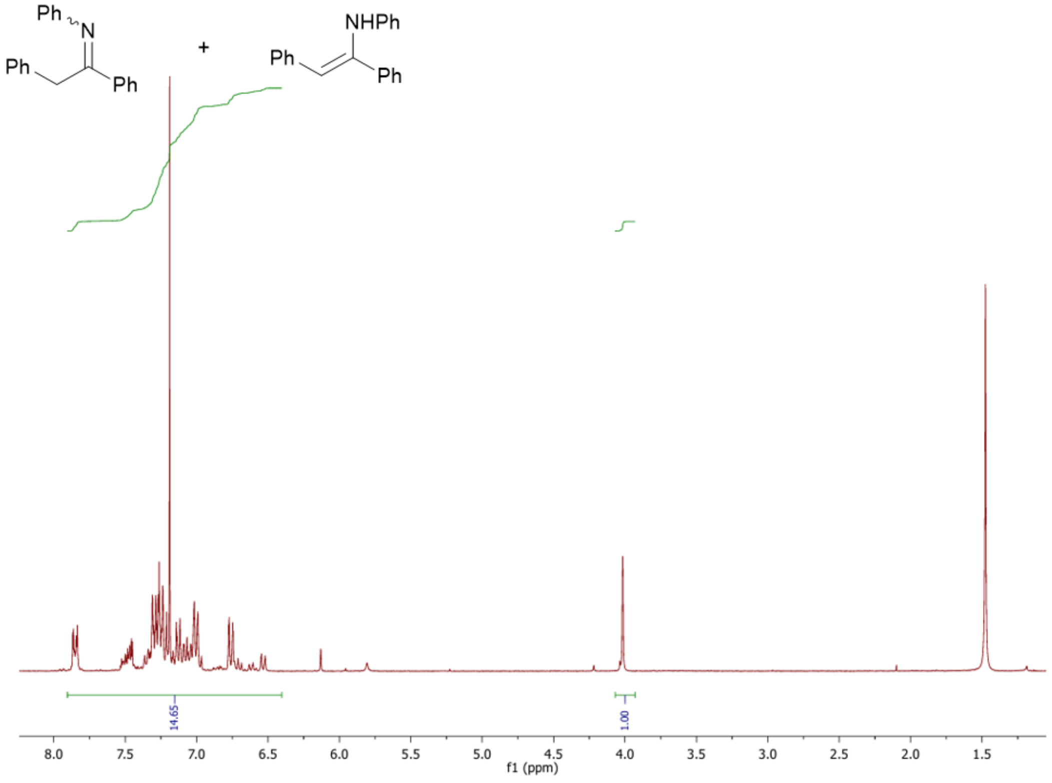

Figure S35. ^{1}H NMR spectrum of *N*,1,2-triphenylethan-1-imine (Scheme 3, **ak**).

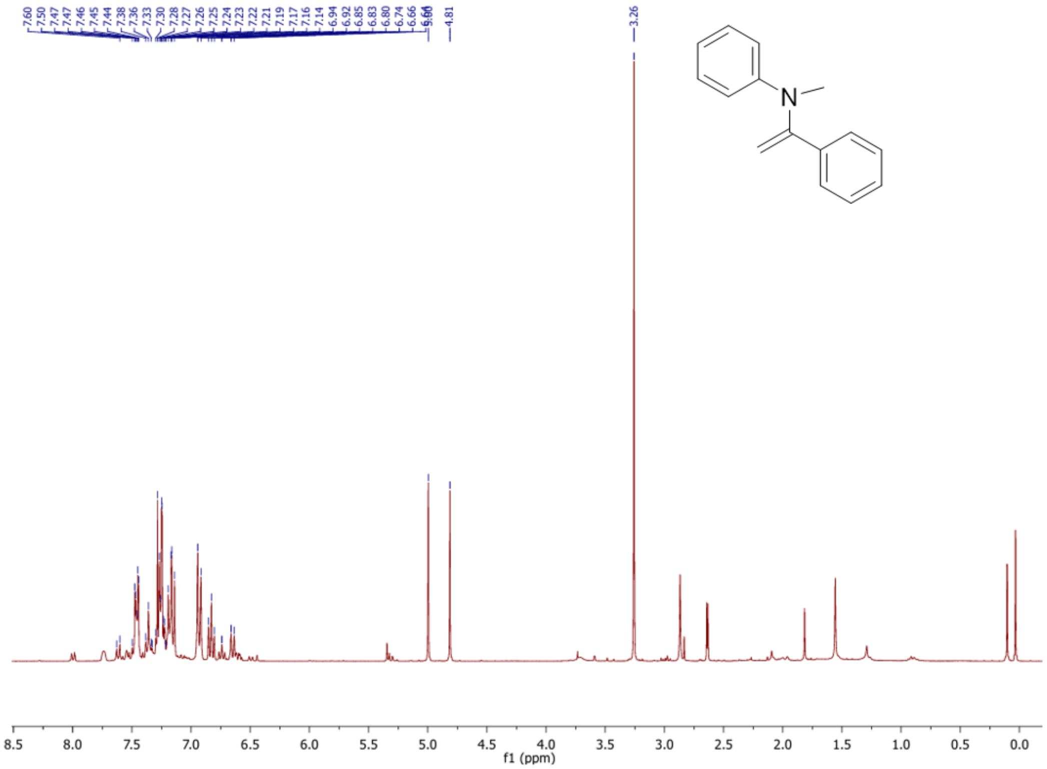

Figure S36. ^{1}H NMR spectrum of methylphenyl(1-phenylvinyl)amine (Scheme 3, **ak**).

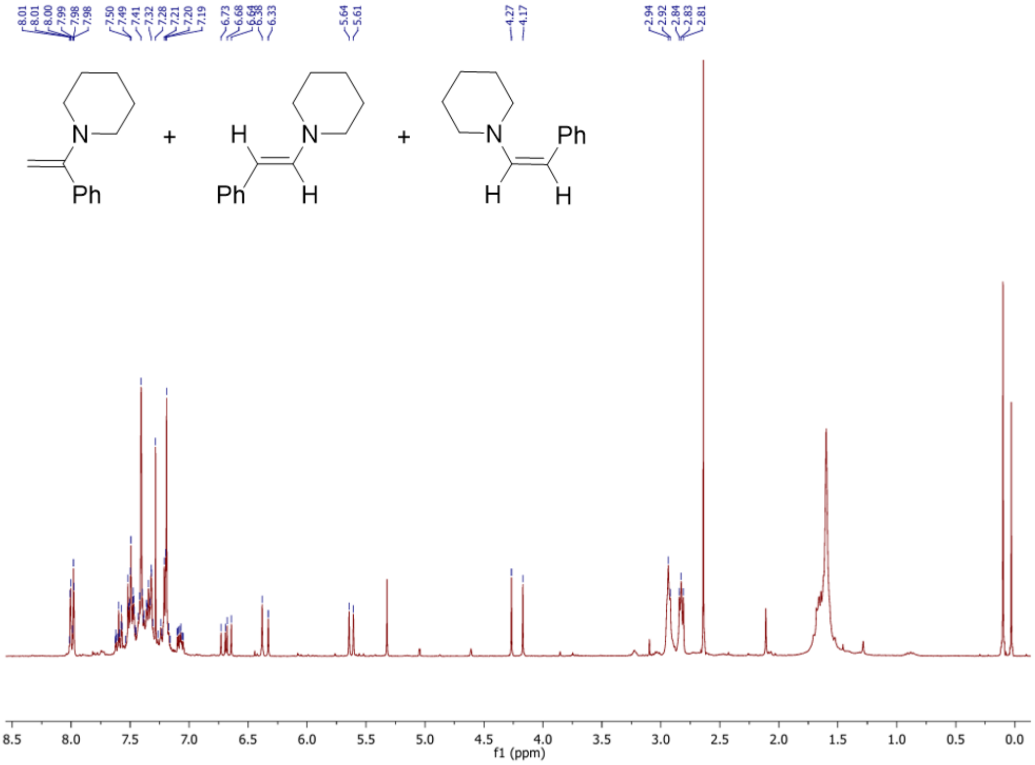

Figure S37. ^{1}H NMR spectrum of 1-(1-phenylvinyl)piperidine and 1-styrylpiperidine (Scheme 3, **am**).

2. X-ray structural data of gold complexes

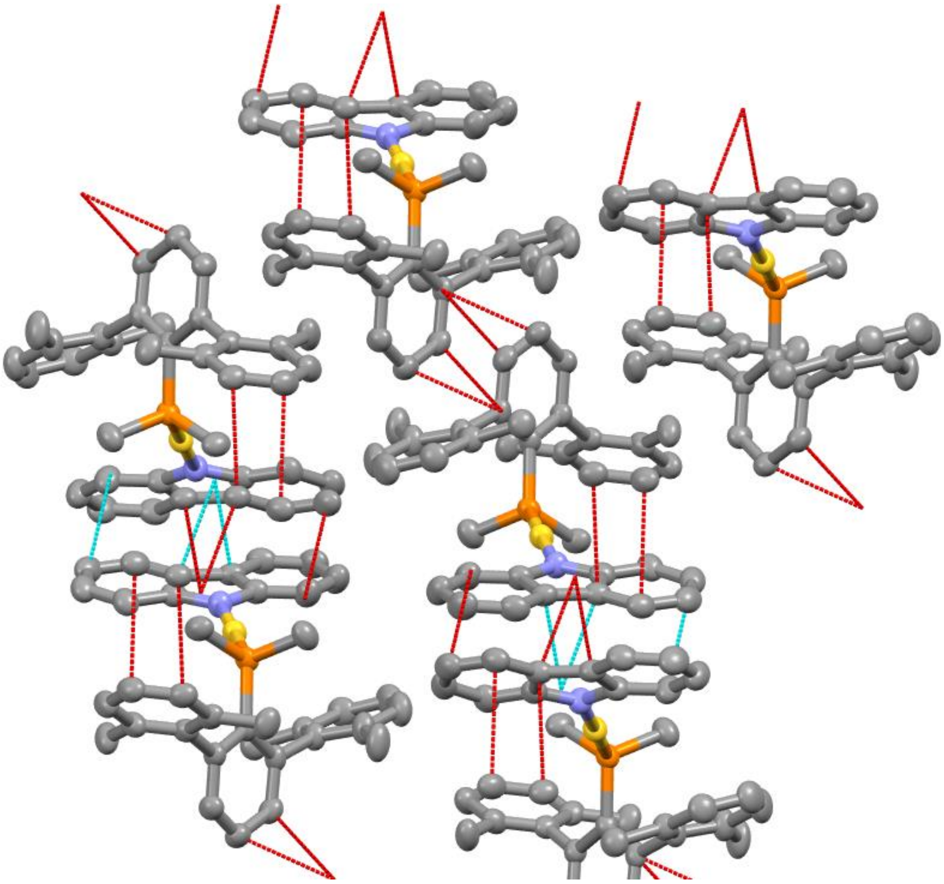

Figure S38. 3D crystal packing of complex **1a** viewed down the *b*-axis. Yellow: gold; orange: phosphorus, blue: nitrogen, red: oxygen; gray: carbon. Thermal ellipsoids at 50% probability and hydrogens atoms were omitted for clarity. The contacts present different colour to show the difference between the intermolecular contacts present.

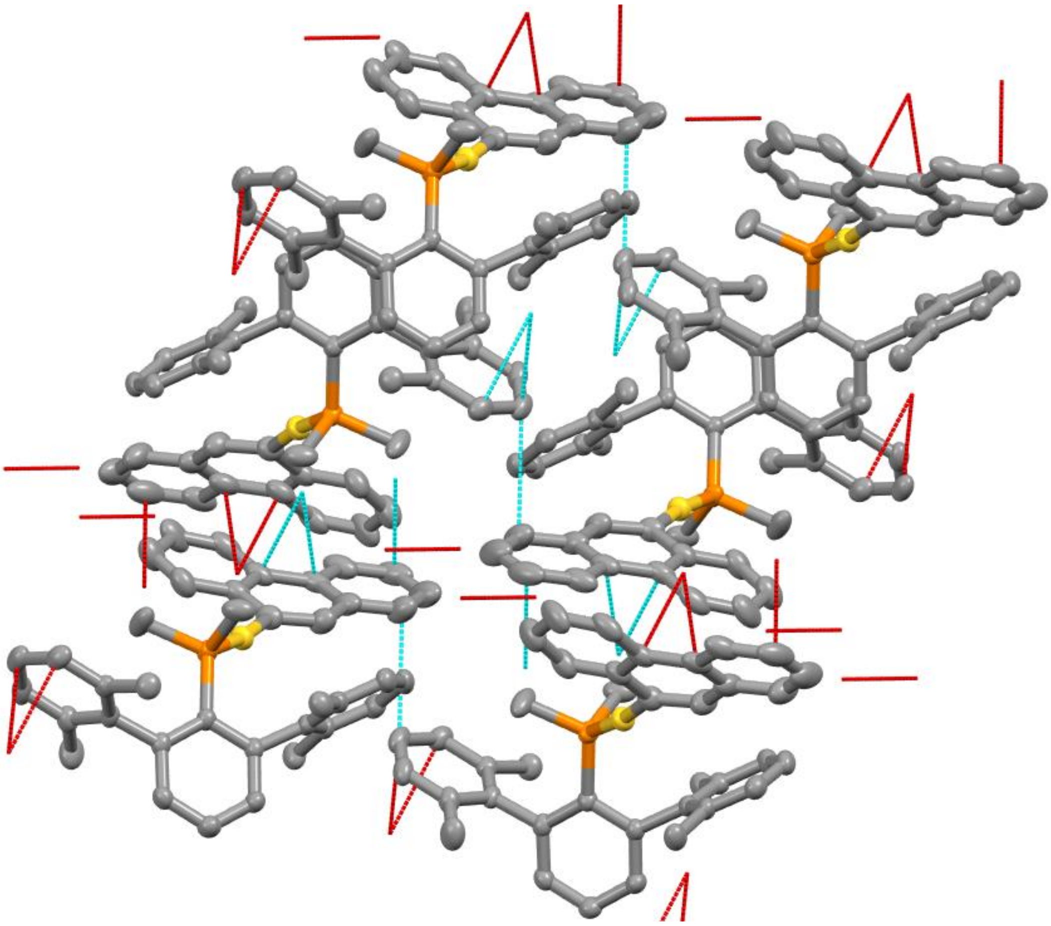

Figure S39. 3D crystal packing of complex **1b** viewed down the *b*-axis. Yellow: gold; orange: phosphorus, blue: nitrogen, red: oxygen; gray: carbon. Thermal ellipsoids at 50% probability and hydrogens atoms were omitted for clarity. The contacts present different colour to show the difference between the intermolecular contacts present.

3. Photophysical characterization

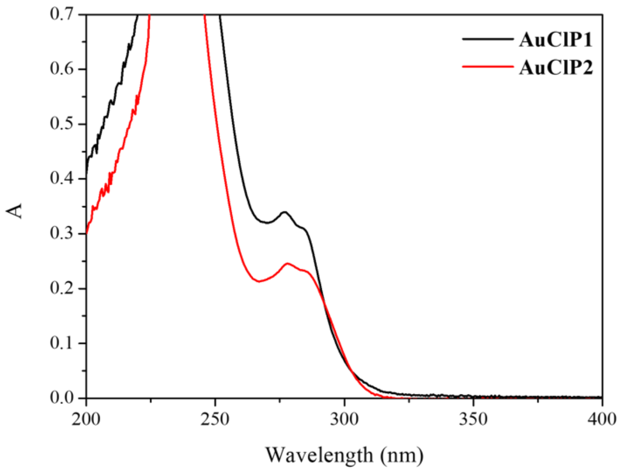

Figure S40. Absorption spectra of compounds AuClPx in dichloromethane under air-equilibrated conditions.

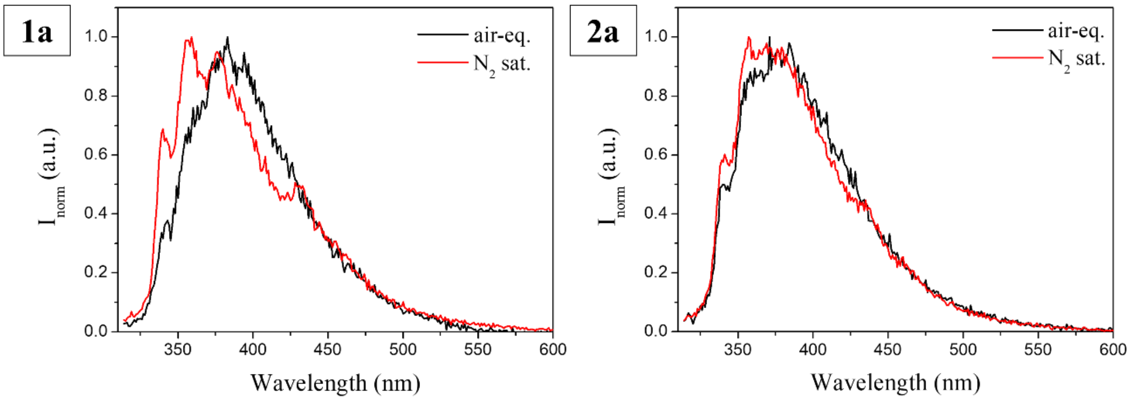

Figure S41. Emission spectra with N_2 saturated atmosphere of dichloromethane solutions of complexes **1a** (left) and **2a** (right) at room temperature. λ_{exc} = 307 nm.

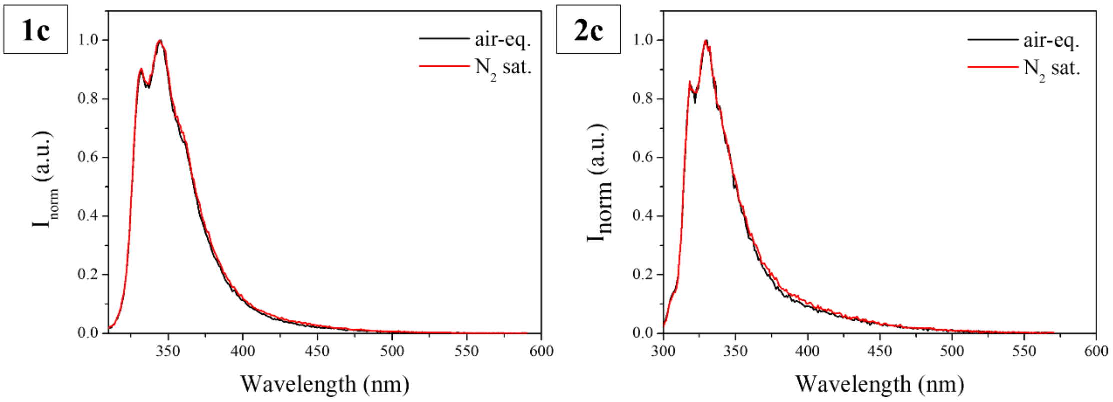

Figure S42. Emission spectra with N_2 saturated atmosphere of dichloromethane solutions of complexes **1c** (left) and **2c** (right) at room temperature. λ_{exc} = 300 nm.

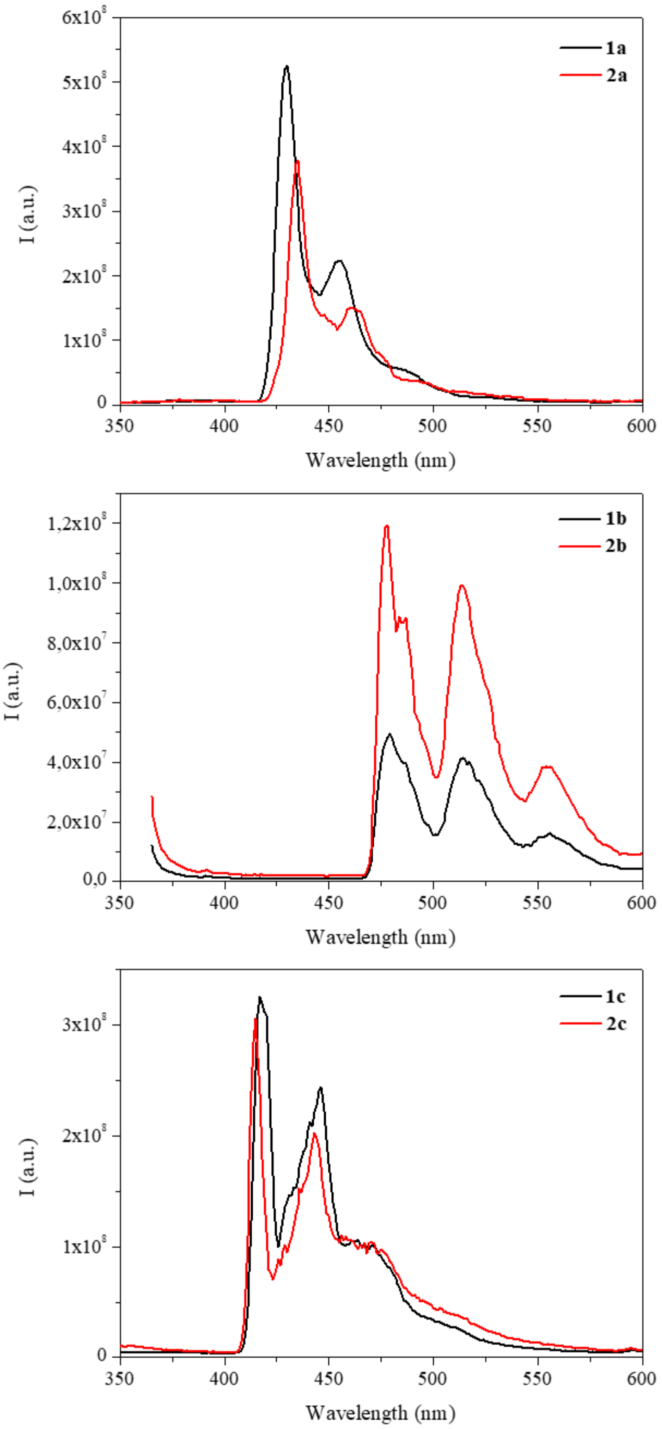

Figure S43. Emission spectra at 77K of dichloromethane solutions of gold(I) derivatives. λ_{exc} = 307 (**1a, 2a**), 355 (**1b, 2b**), 311 (**1c, 2c**) nm.

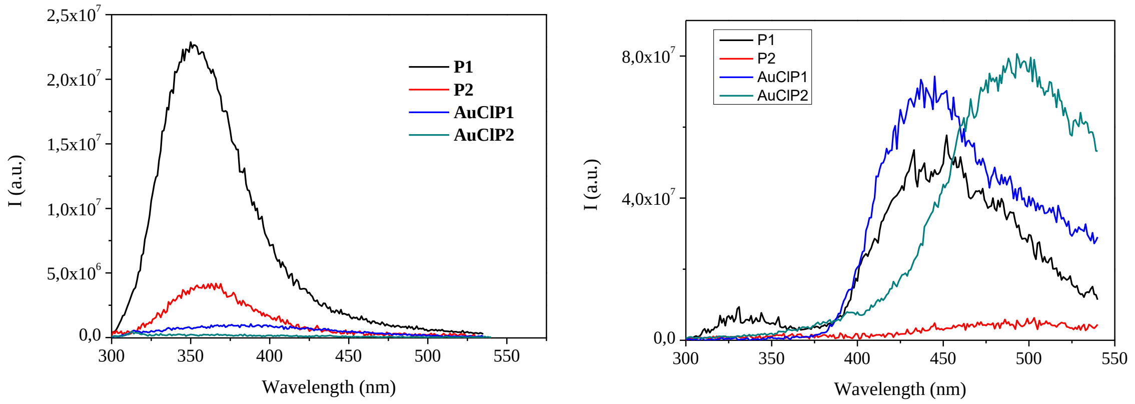

Figure S44. Emission spectra of **Px** and AuCl**Px** precursors in dichloromethane at RT (left) and 77 K (right). λ_{exc} = 290 nm.

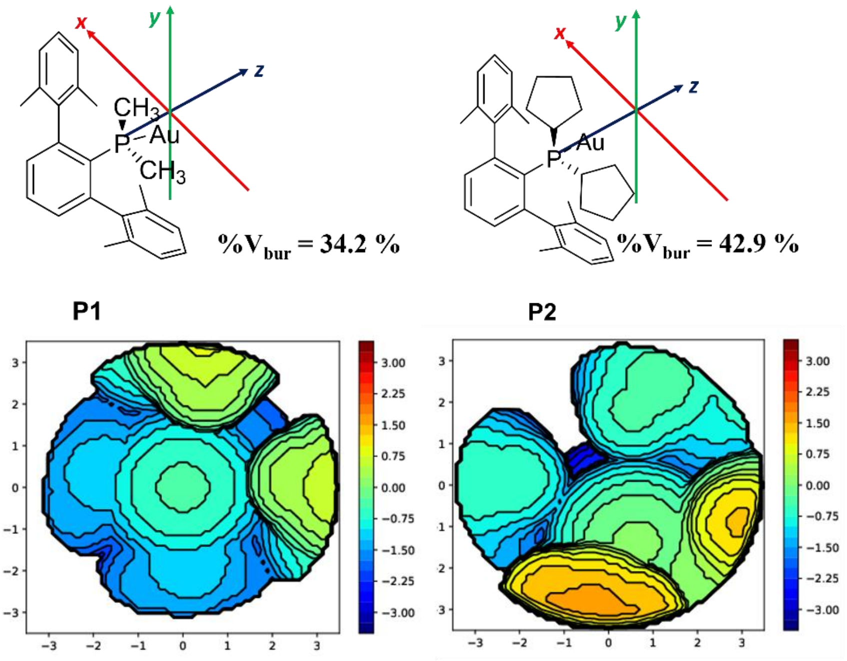

Figure S45. Topographic steric maps and $\%V_{bur}$ values of both phosphanes-gold(I) units (**P1** and **P2**) using their optimized geometry.

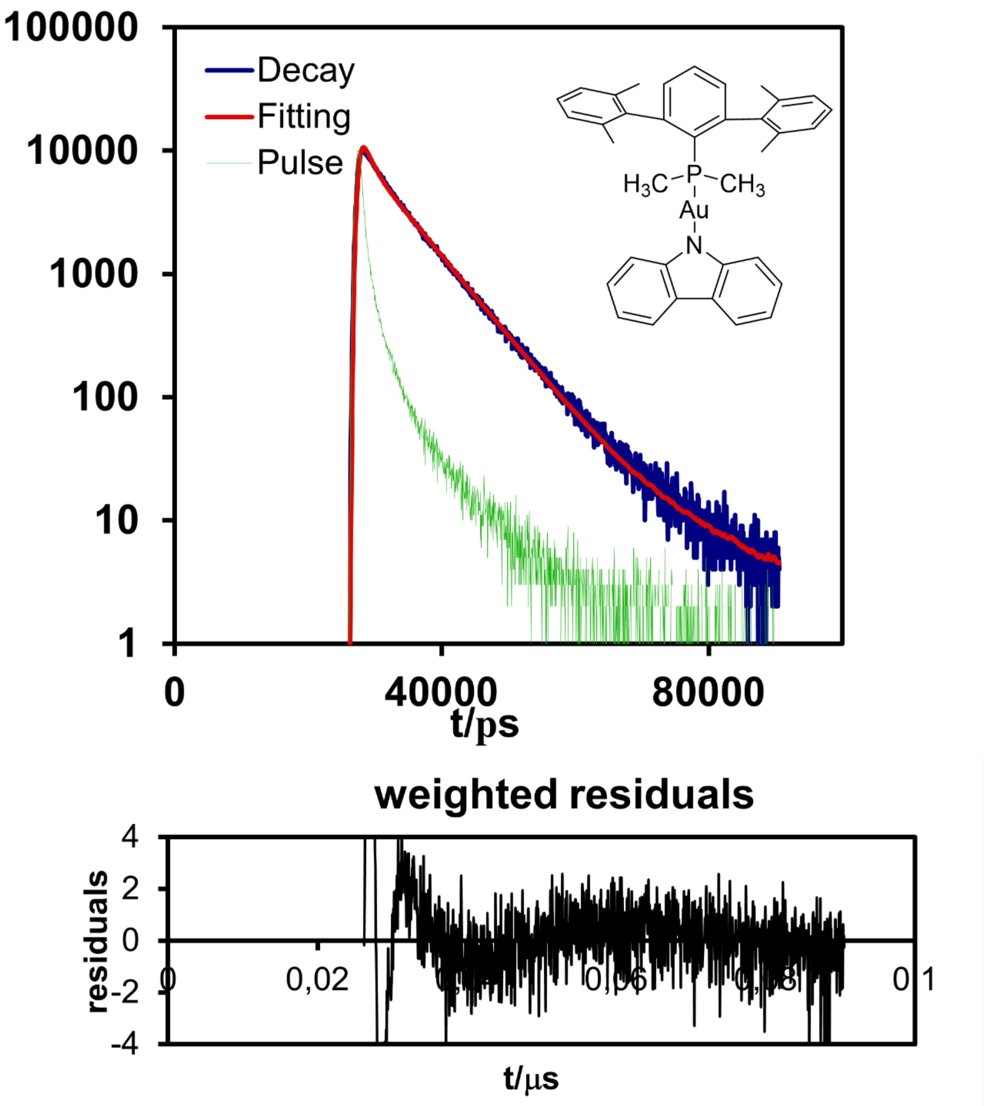

Figure S46. Fluorescence lifetime and residuals of **1a** in dichloromethane solutions in the presence of oxygen.

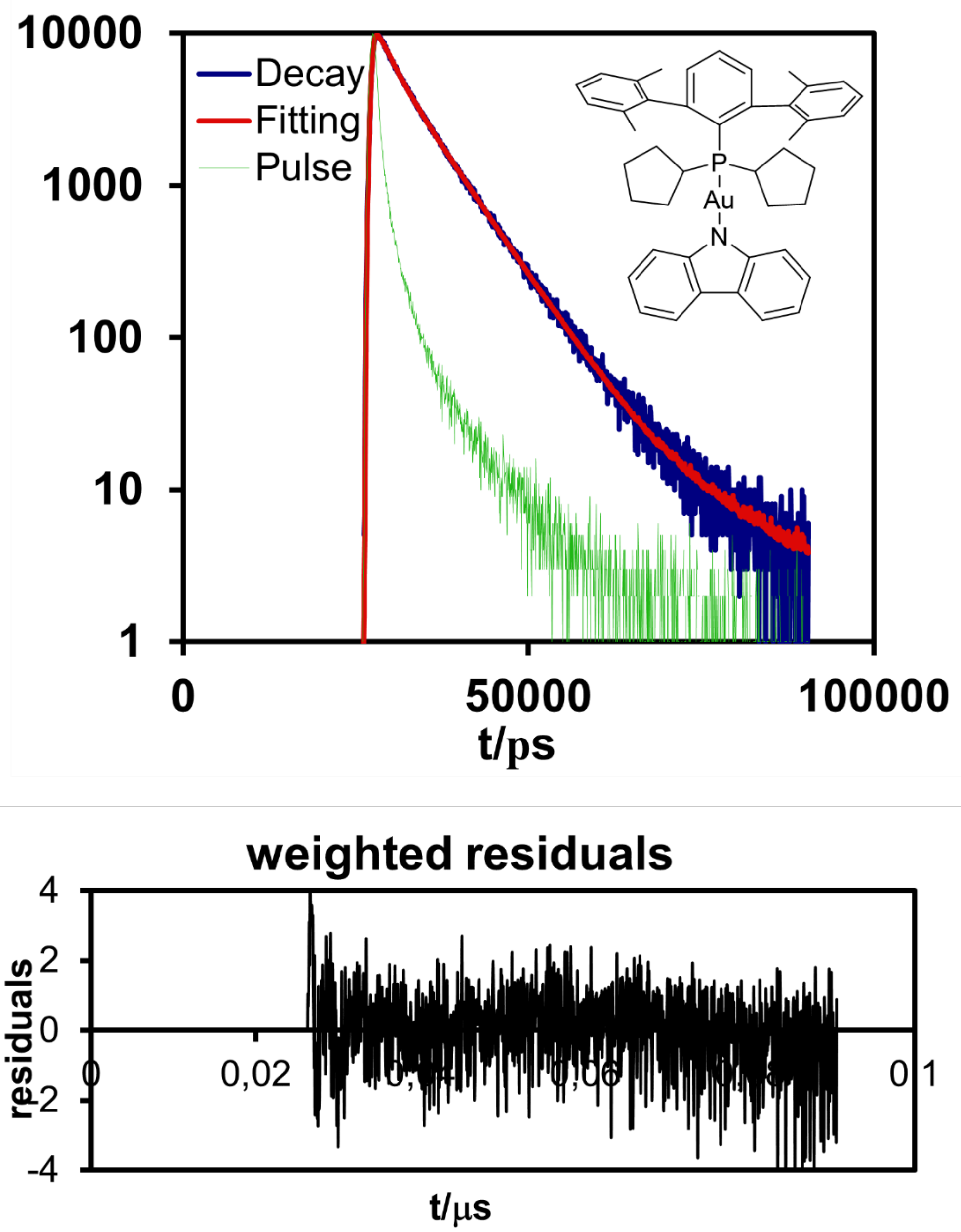

Figure S47. Fluorescence lifetime and residuals of **2a** in dichloromethane solutions in the presence of oxygen.

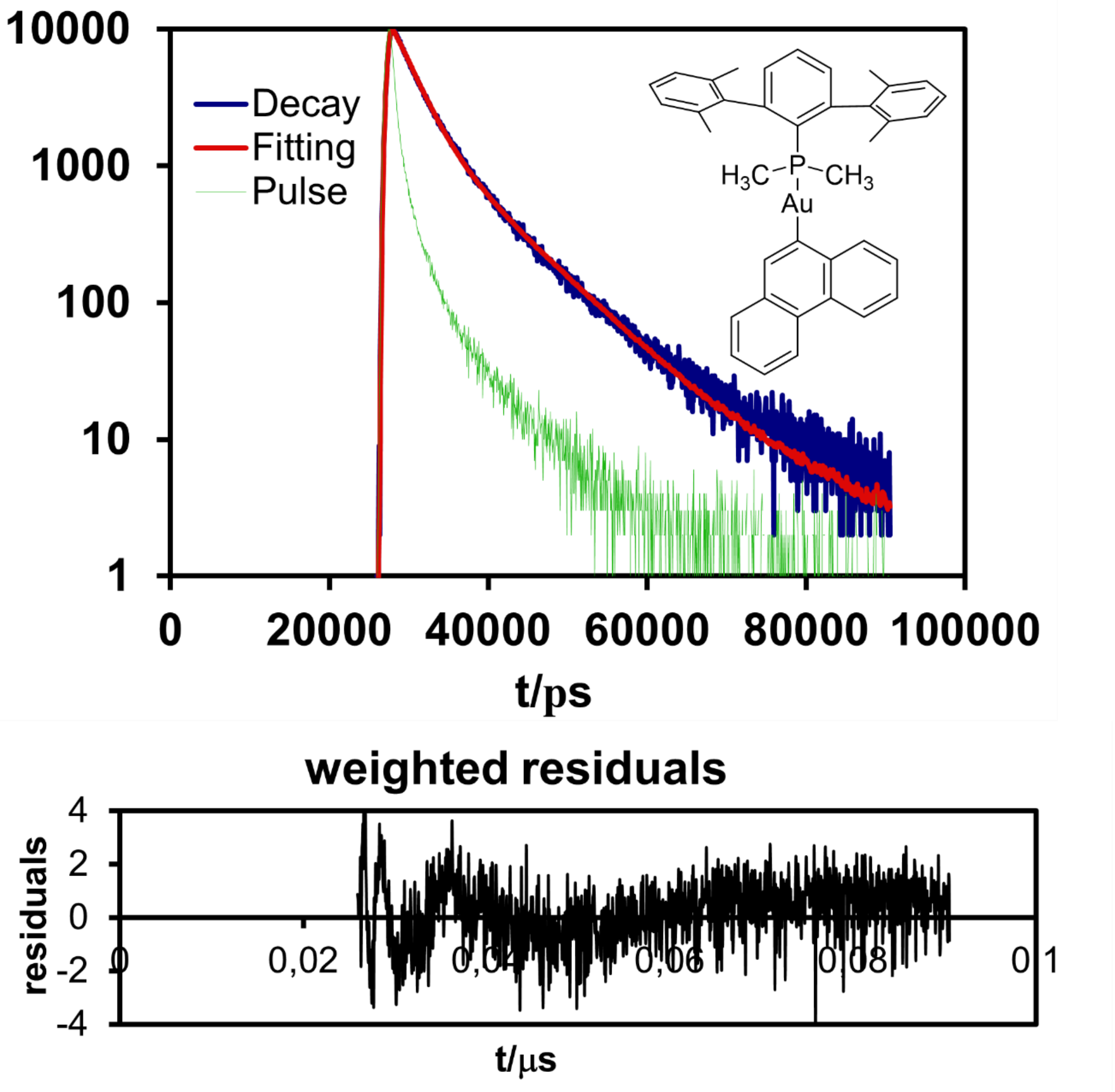

Figure S48. Fluorescence lifetime and residuals of **1b** in dichloromethane solutions in the presence of oxygen.

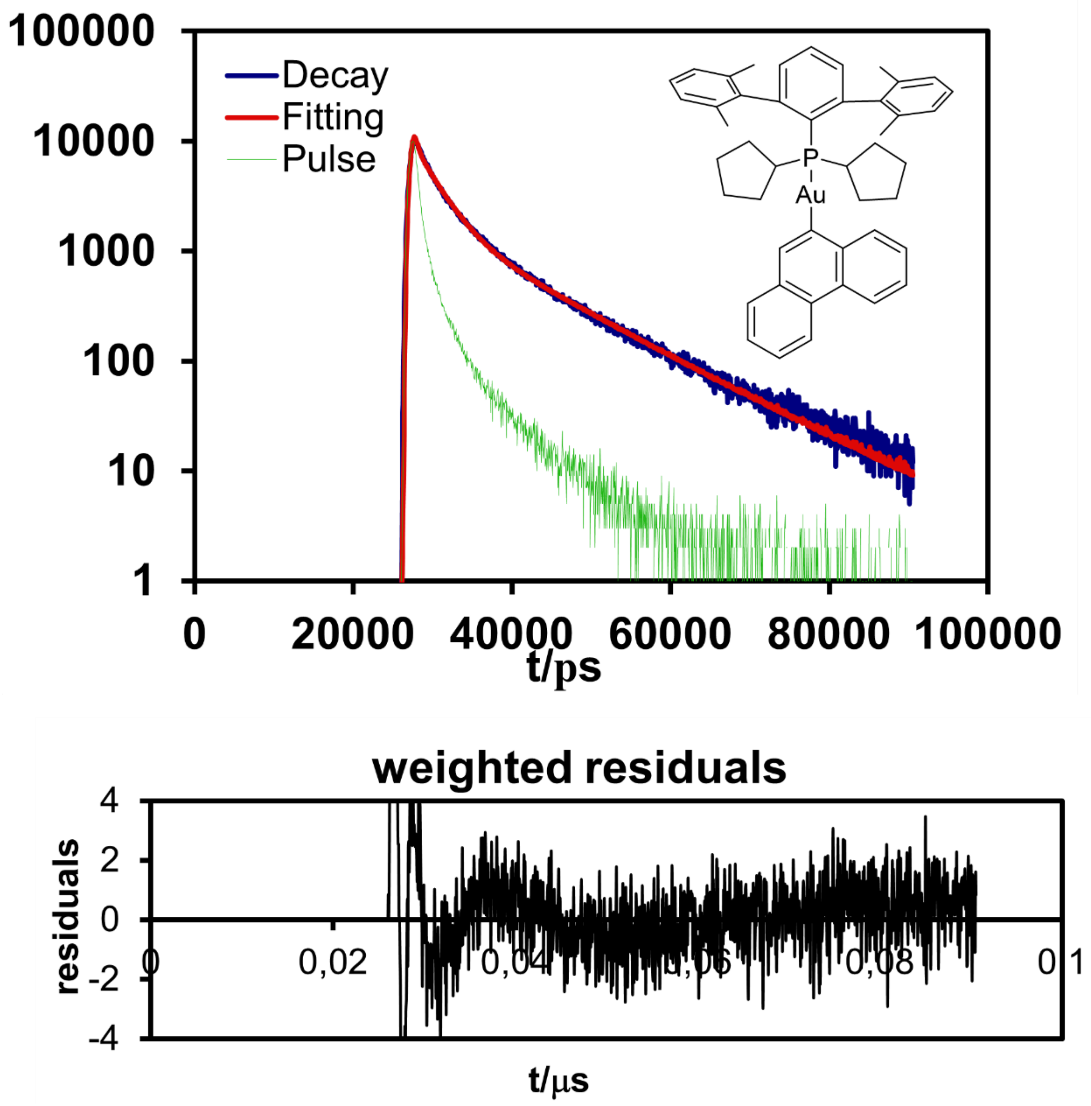

Figure S49. Fluorescence lifetime and residuals of **2b** in dichloromethane solutions in the presence of oxygen.

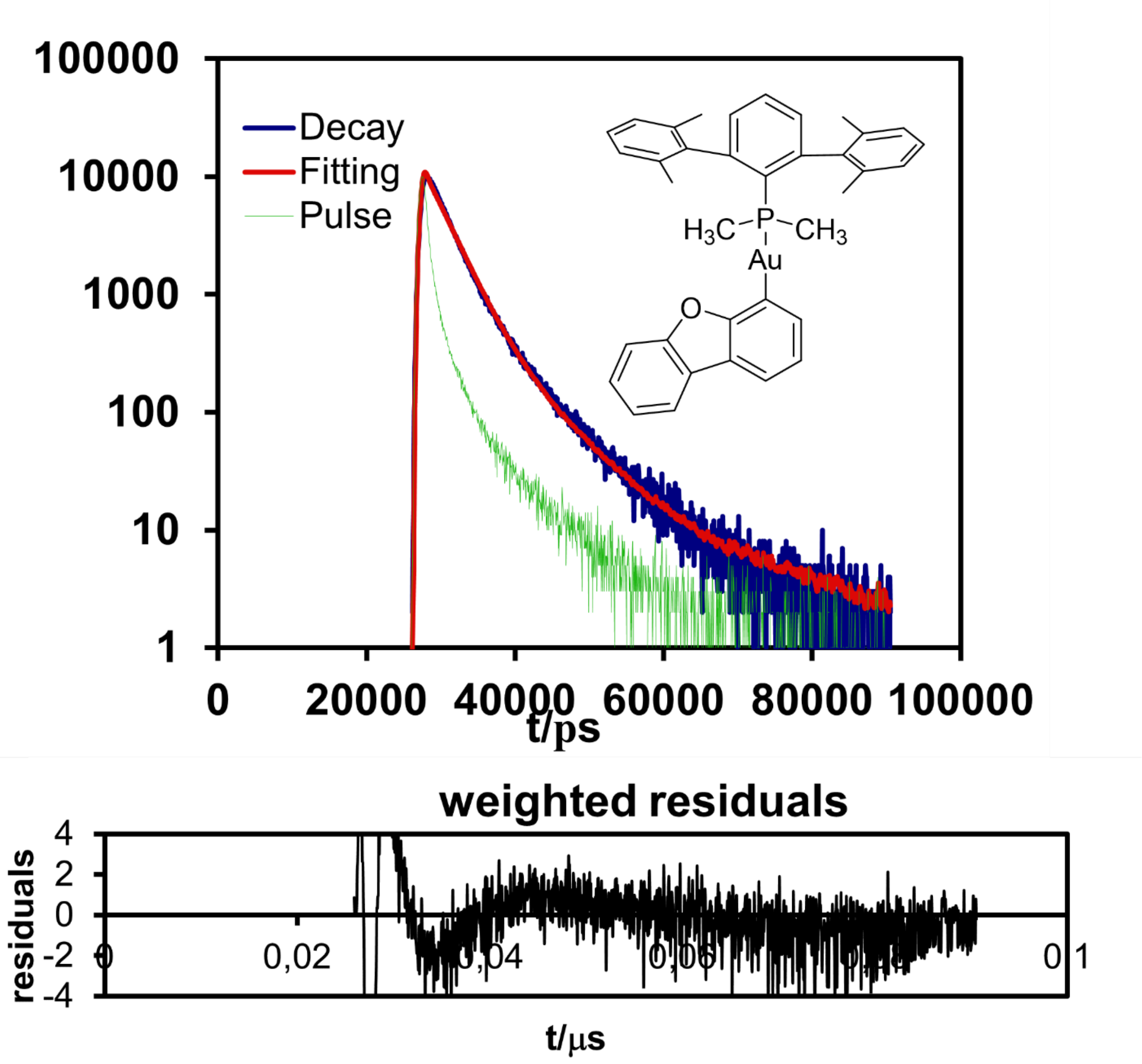

Figure S50. Fluorescence lifetime and residuals of **1c** in dichloromethane solutions in the presence of oxygen.

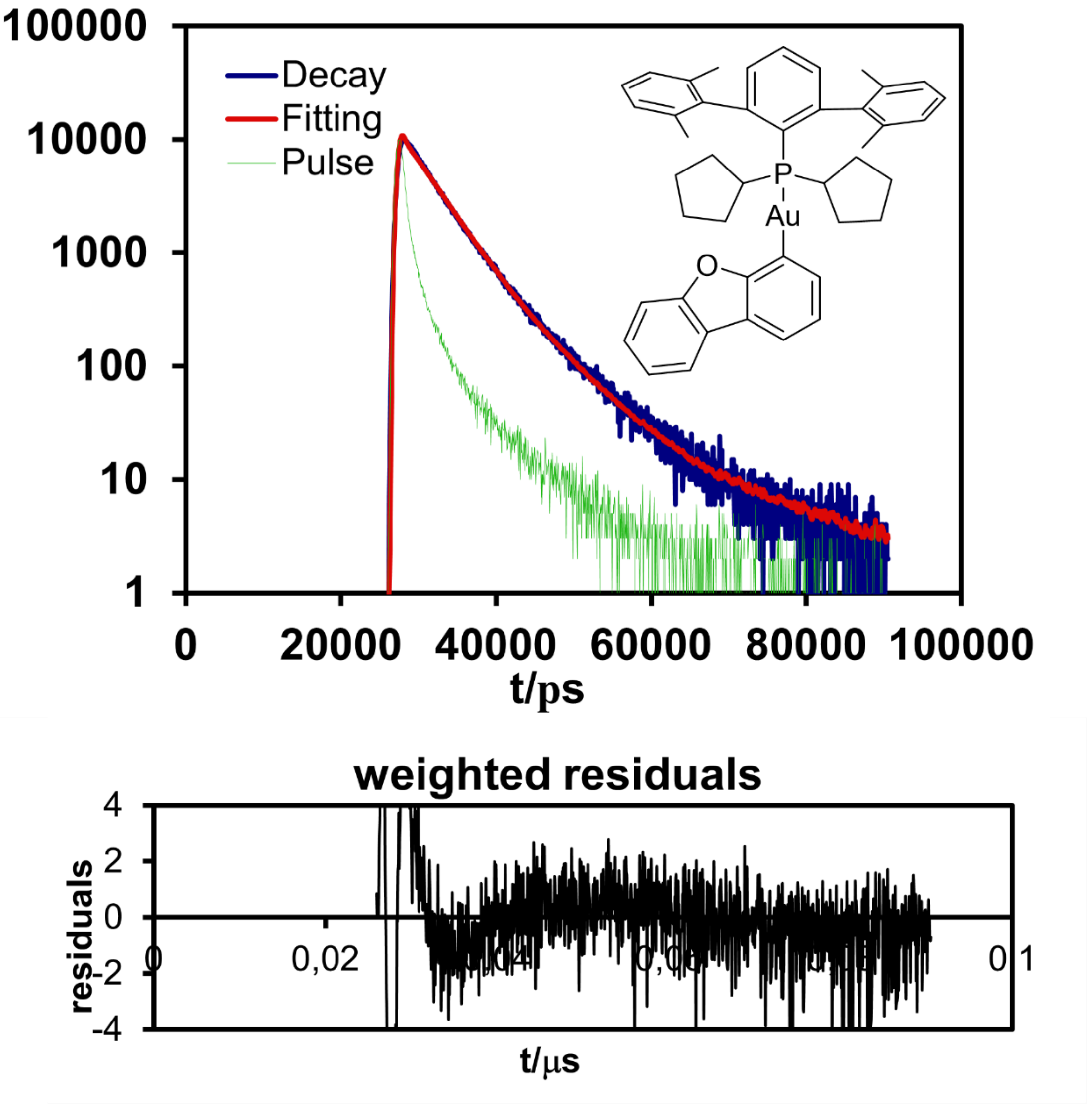

Figure S51. Fluorescence lifetime and residuals of **2c** in dichloromethane solutions in the presence of oxygen.

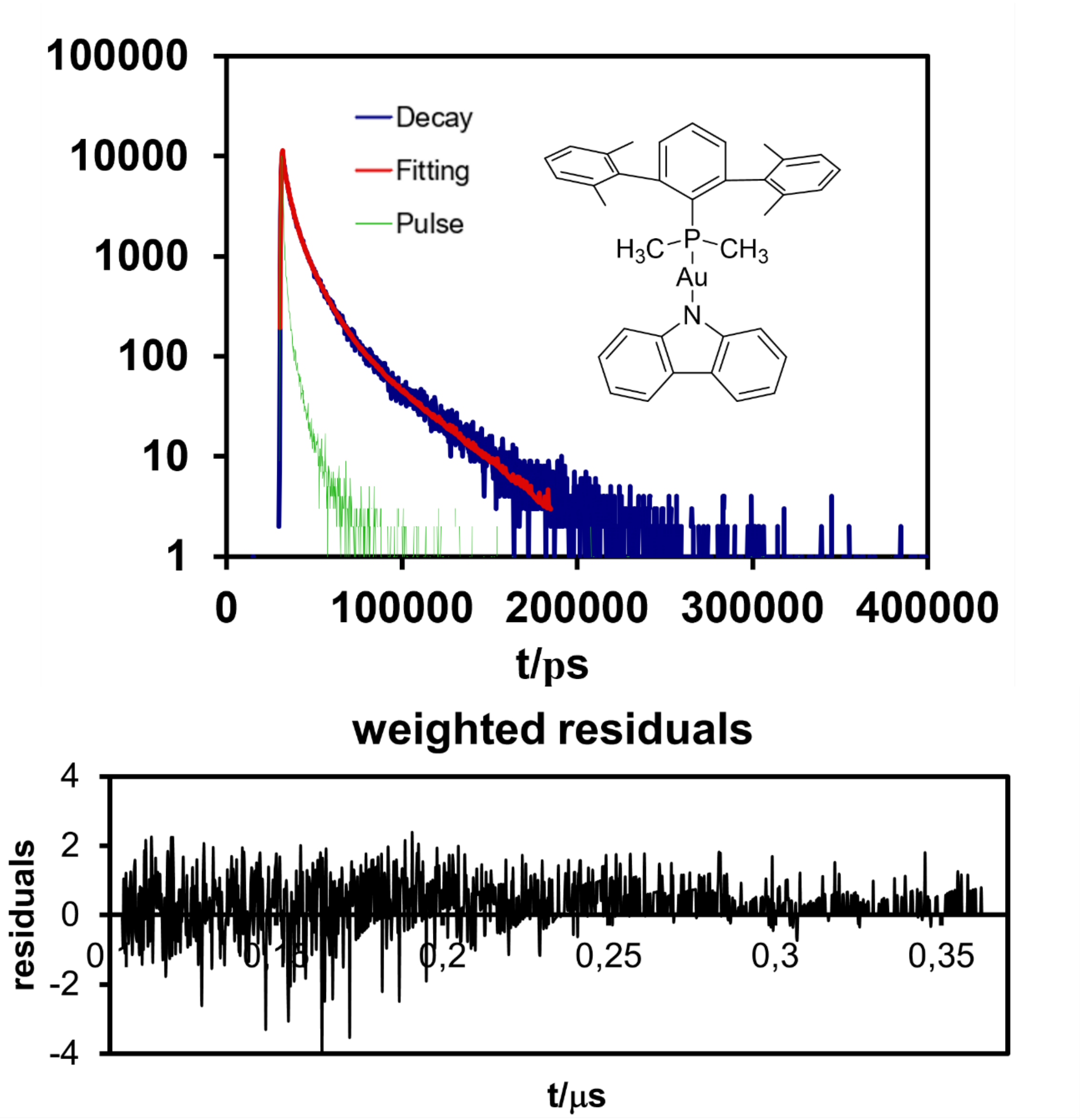

Figure S52. Fluorescence lifetime and residuals of **1a** in dichloromethane solutions in the exclusion of oxygen.

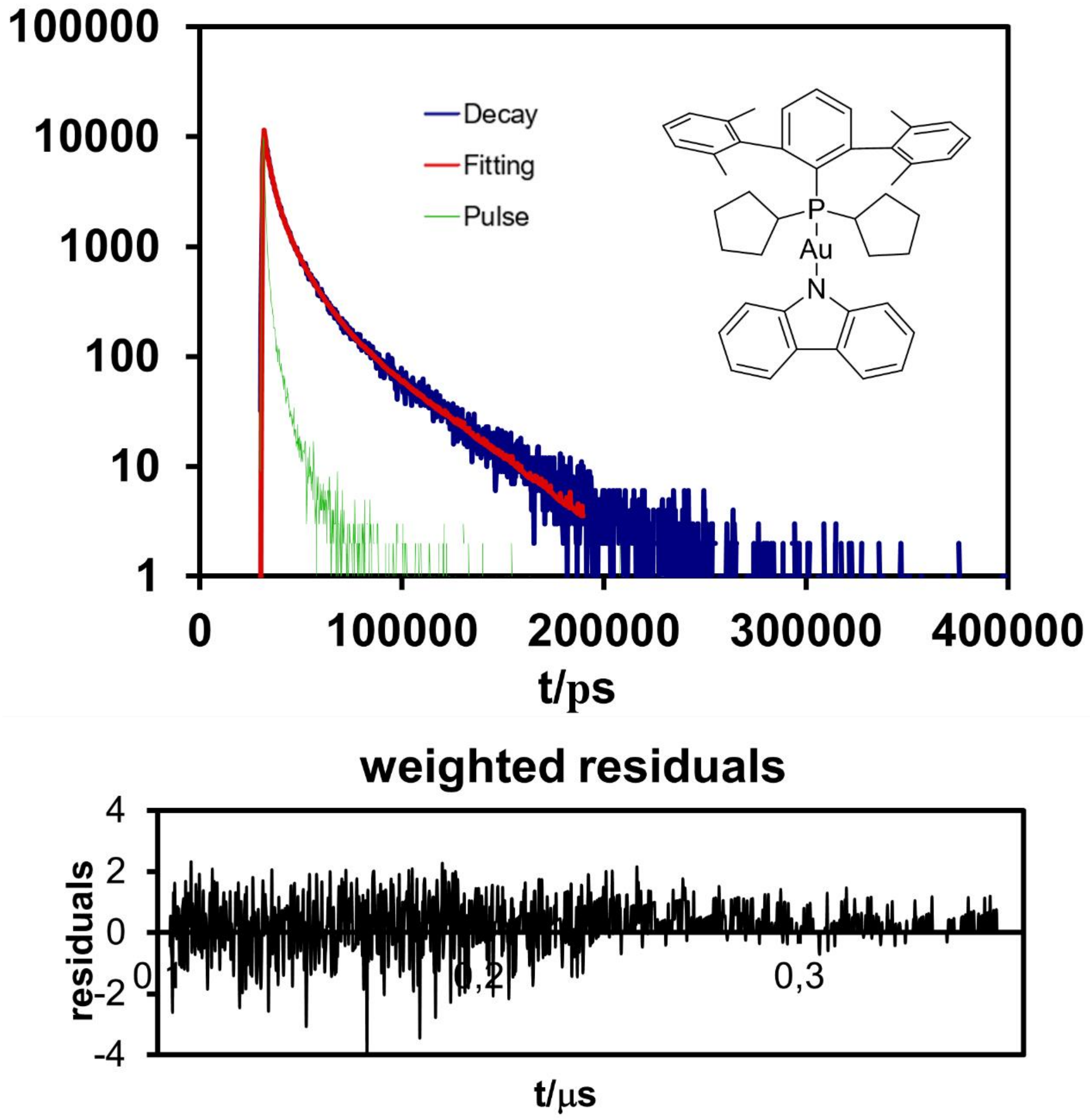

Figure S53. Fluorescence lifetime and residuals of **2a** in dichloromethane solutions in the exclusion of oxygen.

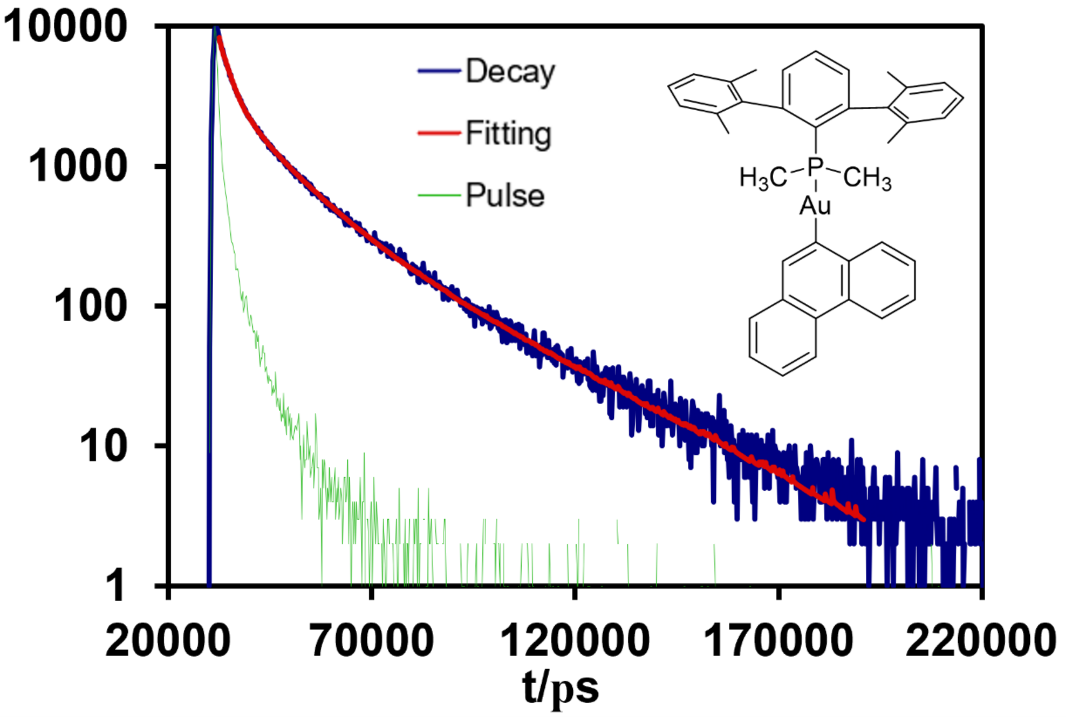

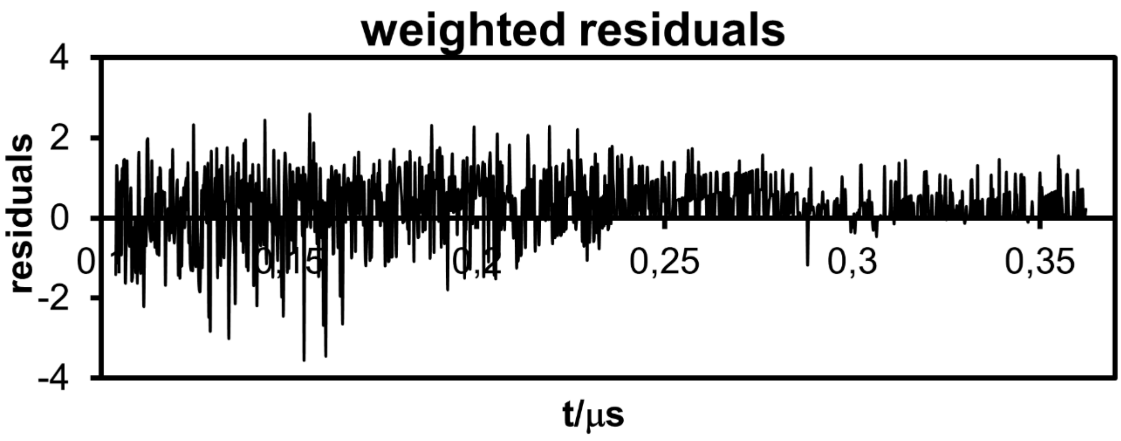

Figure S54. Fluorescence lifetime and residuals of **1b** in dichloromethane solutions in the exclusion of oxygen.

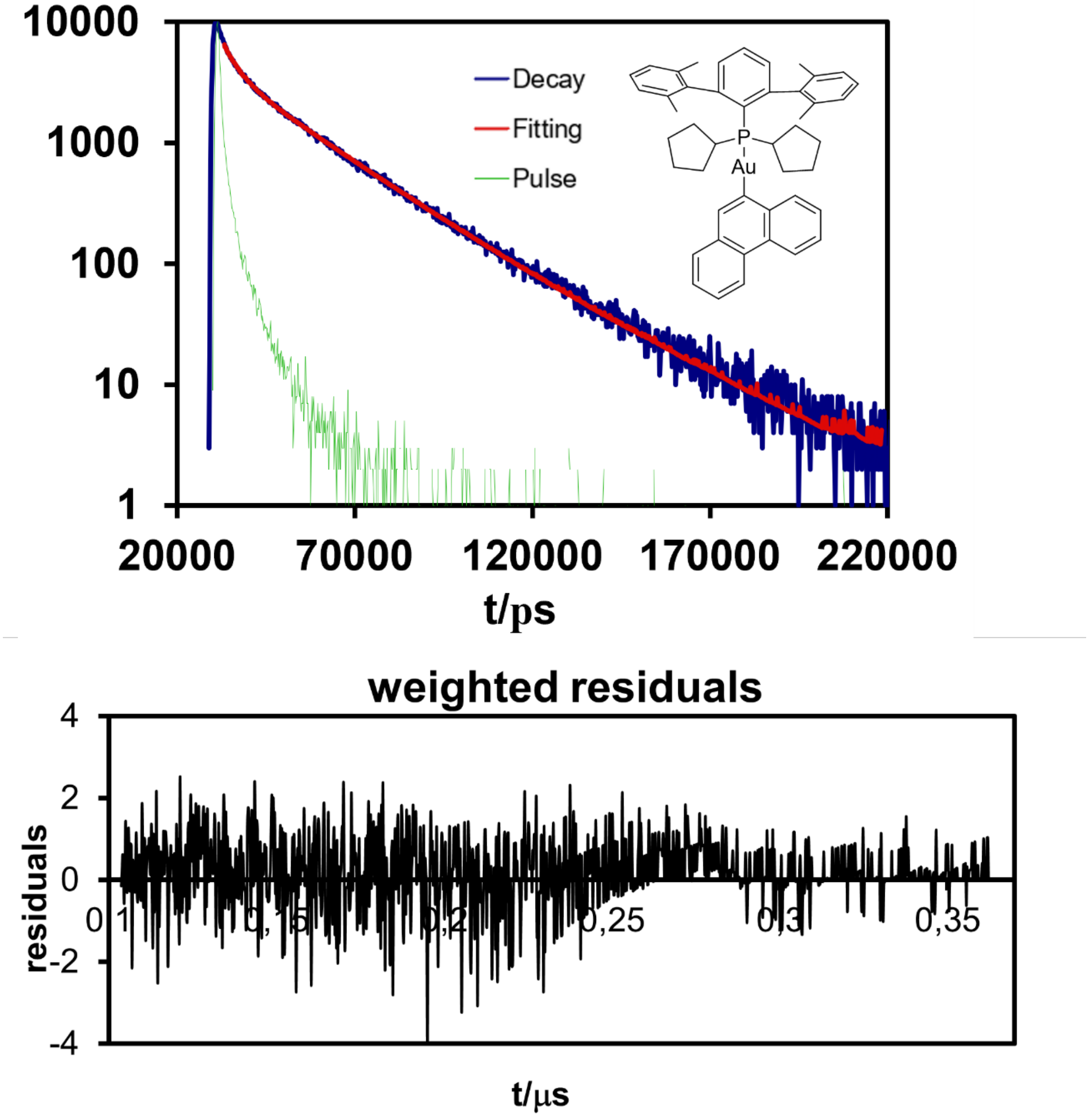

Figure S55. Fluorescence lifetime and residuals of **2b** in dichloromethane solutions in the exclusion of oxygen.

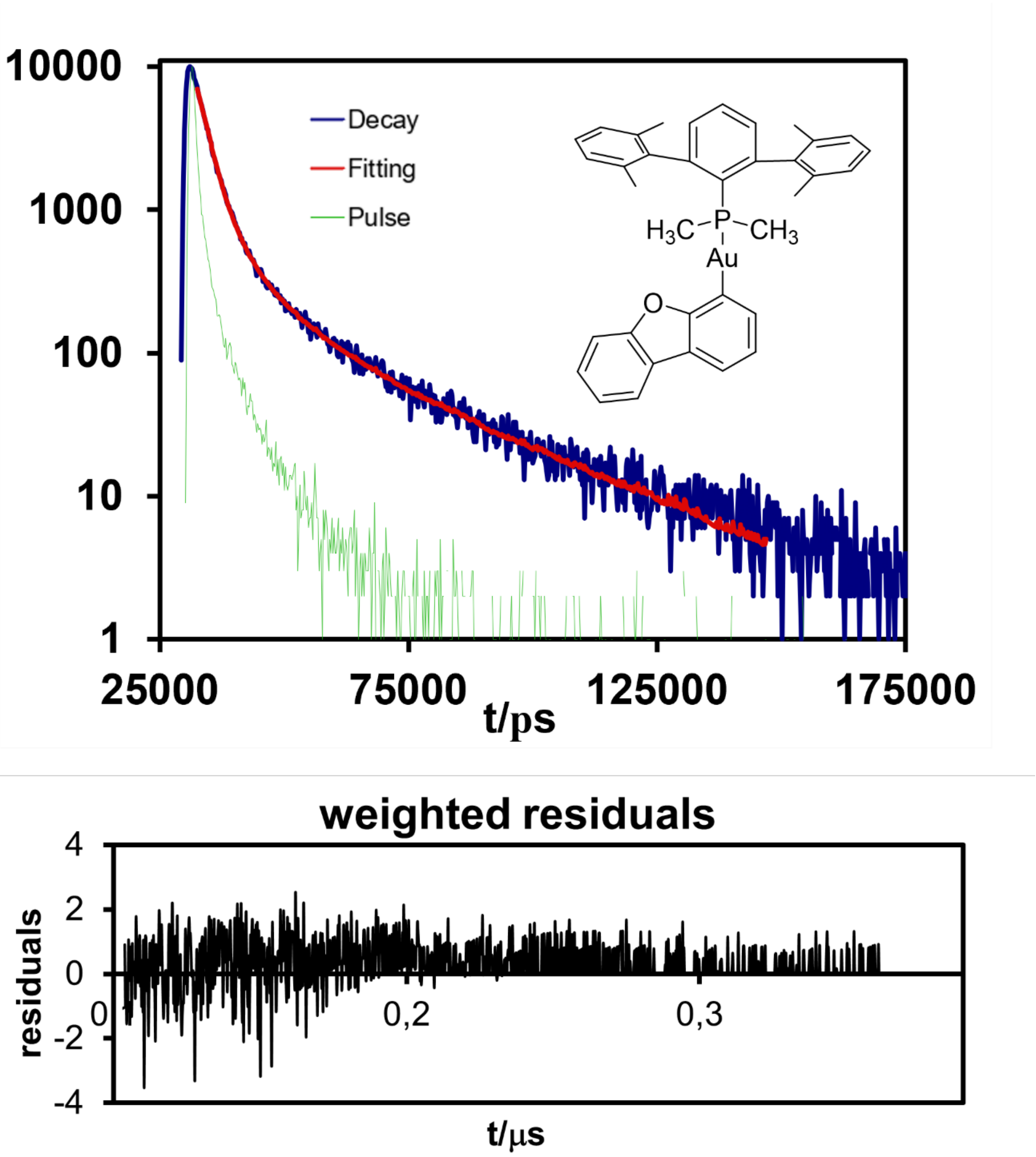

Figure S56. Fluorescence lifetime and residuals of **1c** in dichloromethane solutions in the exclusion of oxygen.

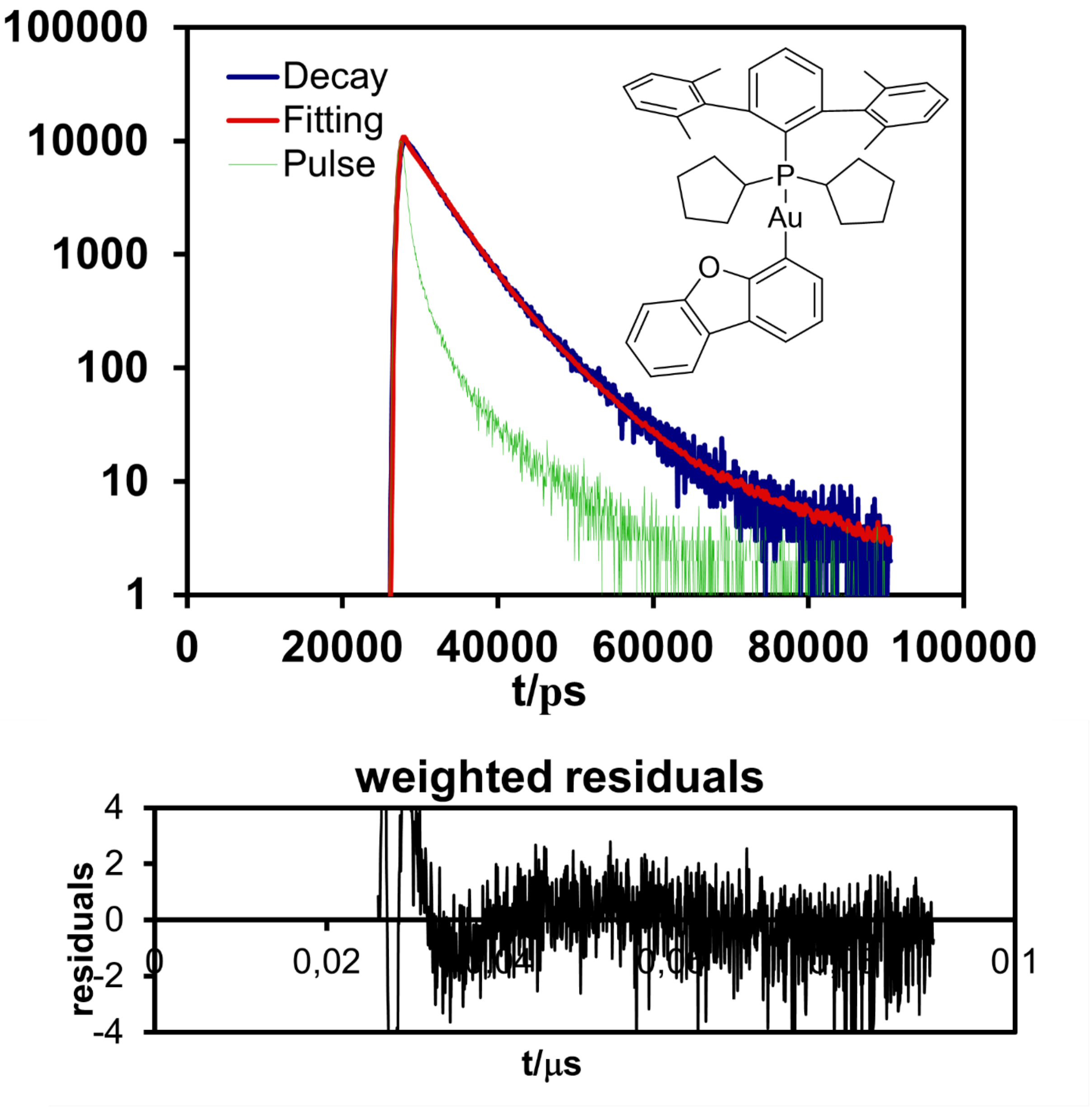

Figure S57. Fluorescence lifetime and residuals of **2c** in dichloromethane solutions in the exclusion of oxygen.

***ns*-Transient Absorption**

For that, we measured Δ_{OD1} which belongs to the amplitude of the depletion experiment (Bleaching), and Δ_{OD2} which is the amplitude of the transient experiment (Figure 5 below) that allowed us to calculate the ϕ_{T1} and τ for all the compounds (see below, Experimental Section for details).

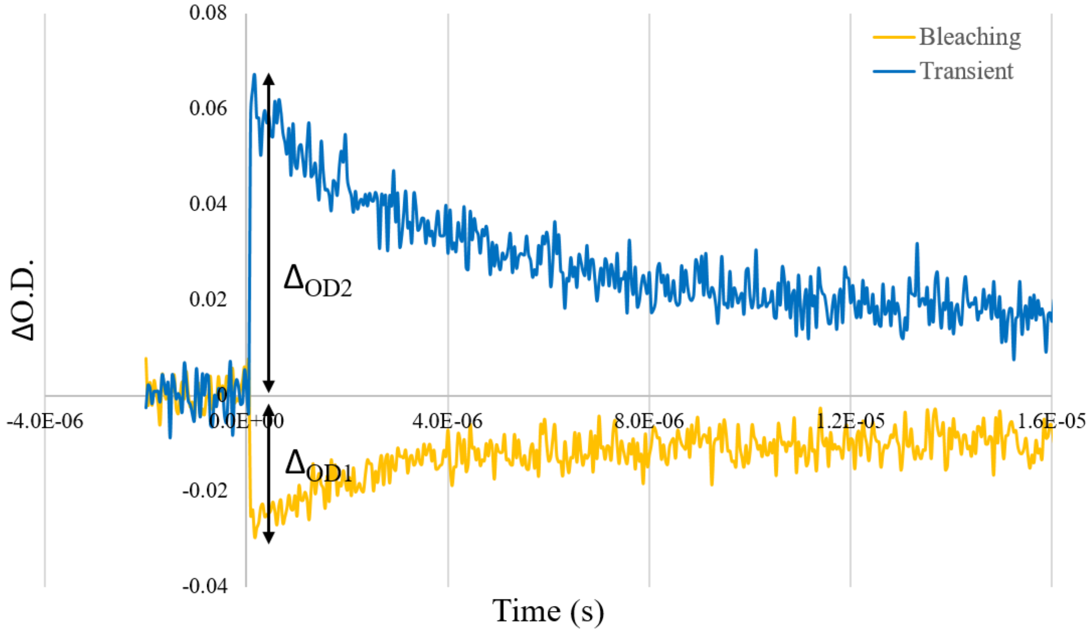

Figure S58. Bleaching and Transient spectra of **2a** in the absence of oxygen.

The transient absorptions were collected after a 365 nm laser pulse in the case of **a** and **b** complexes and after a 266 nm laser pulse in the case of **c** derivatives; the calculated Φ_T values are displayed in Table 4 above.

For **a** and **c** derivatives we worked with the ***Singlet Depletion*** methodology, which consists in, firstly, calculate the ε_T using equation 1 with the experimental ε_S (see Table 2) and the optical density amplitude (Δ_{OD1} belongs to the amplitude of the depletion experiment and Δ_{OD2} to the amplitude from the transient experiment), which can be calculated by the fitting using a monoexponential equation

$$\varepsilon_T = \varepsilon_S \frac{\Delta OD2}{\Delta OD1} \qquad (1)$$

After that, we could know the Φ_T thanks to the “singlet-depletion” method (eq 2) using benzophenone as the reference actinometer

$$\phi_T = \phi_{Tstd} \cdot \frac{\Delta OD2}{\Delta OD3} \cdot \frac{\varepsilon_{Tstd}}{\varepsilon_T} \qquad (2)$$

optically matching a benzophenone solution in acetonitrile (Φ_T = 1; εTstd = 6500 cm^{-1} M^{-1} at 520 nm) with the absorption samples at their corresponding laser excitation wavelength. The extinction coefficient of the triplet–triplet absorption at 600nm (**1a**, **2a**), 450nm (**1c**) and 400nm (**2c**) was calculated from depletion at 300nm (**1a**, **2a**) and 280nm (**1c**,**2c**) using the extinction coefficient of the ground-state absorption at that wavelength, ε_T, previously measured.

For b derivatives, the measured value of the quantum yield of singlet oxygen production was used as the lower limit of the Φ_T of the photosensitizer;

$$\Phi_\Delta = \Phi_T\, \Phi_{ET} \qquad (3)$$

Where Φ_Δ is the quantum yield of singlet oxygen production, Φ_T is the quantum yield of triple formation and is the efficiency of energy transfer from the triplet of the sensitizer to molecular oxygen.

When the efficiency of energy transfer to oxygen is close to the unity yields, then

$$\Phi_\Delta = \Phi_T$$

Lower values of Φ_{ET} efficiencies lead to higher values of Φ_T. and as a consequence, Φ_Δ is a reliable lower limit of Φ_T.

The rate constants related to the intersystem crossing (k_{ISC}) and the $S_0 \leftarrow S_1$ internal conversion transition (k_{IC}) have been calculated in non-aerated conditions, to suppress the oxygen-quenching phenomenon, which is involved in the deactivation constants, by equations (2) and (3) below:

$$k_{ISC} = \frac{\phi_{T,N2}}{\tau_{S,N2}} \quad (2);\; k_{IC} = k_{nr}^S - k_{ISC} \quad (4)$$

where $\tau_{S,N2}$ is the lifetime of the $S_0 \leftarrow S_1$ decay measured in the absence of oxygen, to study this lifetime without the possibility of quenching processes, which could modify the population of the excited states and, therefore, their lifetime value. To know k_r^S, equation (4) can be used:

$$k_r^S = \frac{\phi_{Fluo}}{\tau_S} \qquad (5)$$

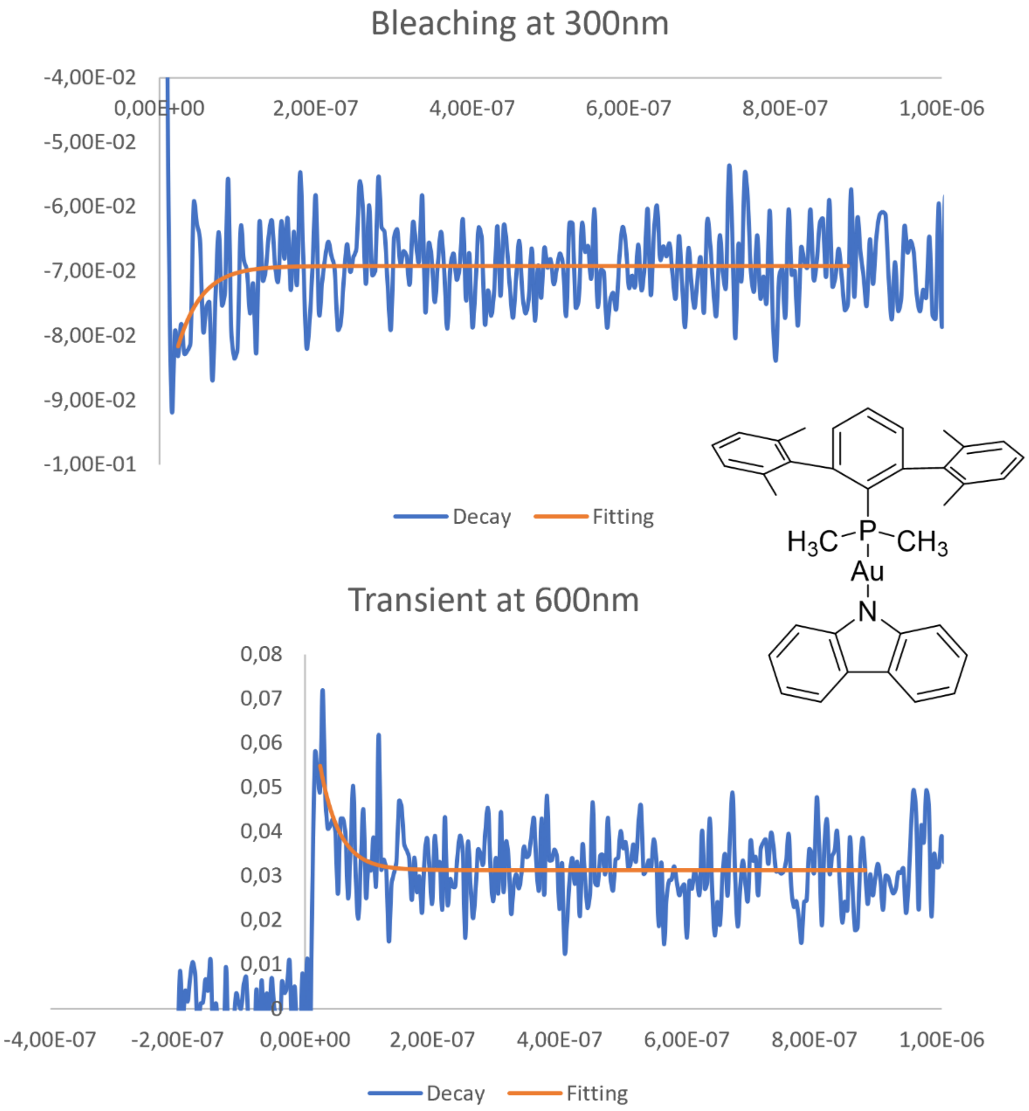

Figure S59. Bleaching and Transient spectra of **1a** in the presence of oxygen.

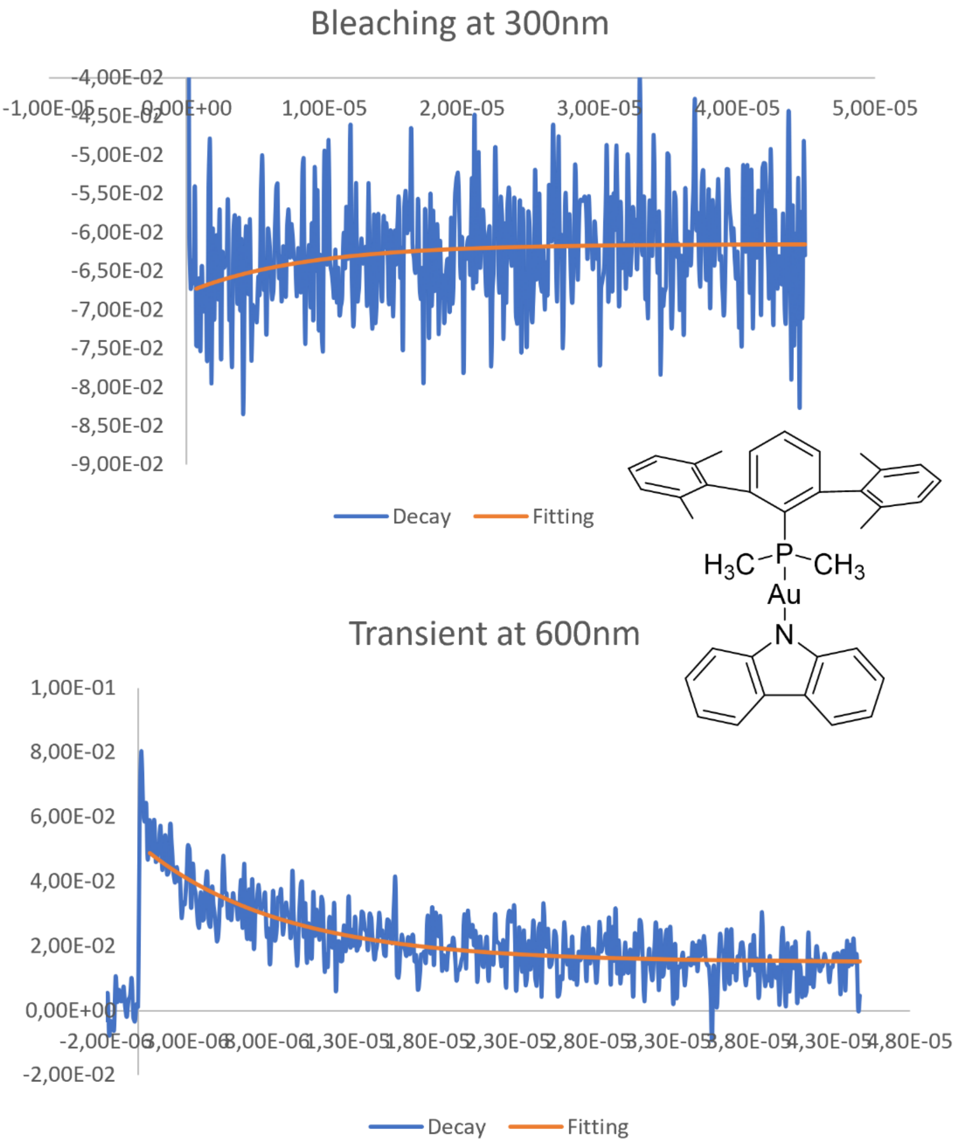

Figure S60. Bleaching and Transient spectra of **1a** in the exclusion of oxygen.

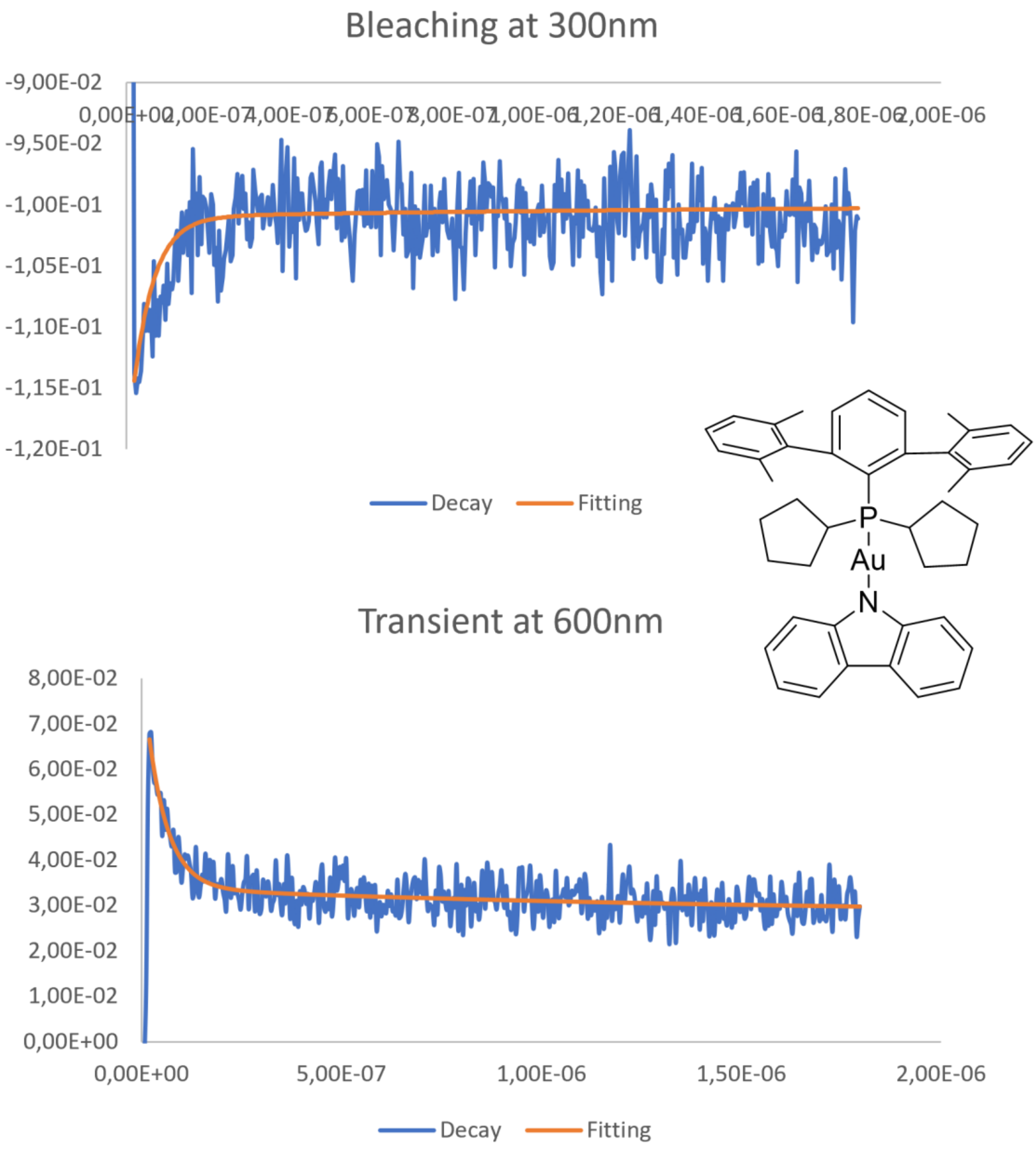

Figure S61. Bleaching and Transient spectra of **2a** in the presence of oxygen.

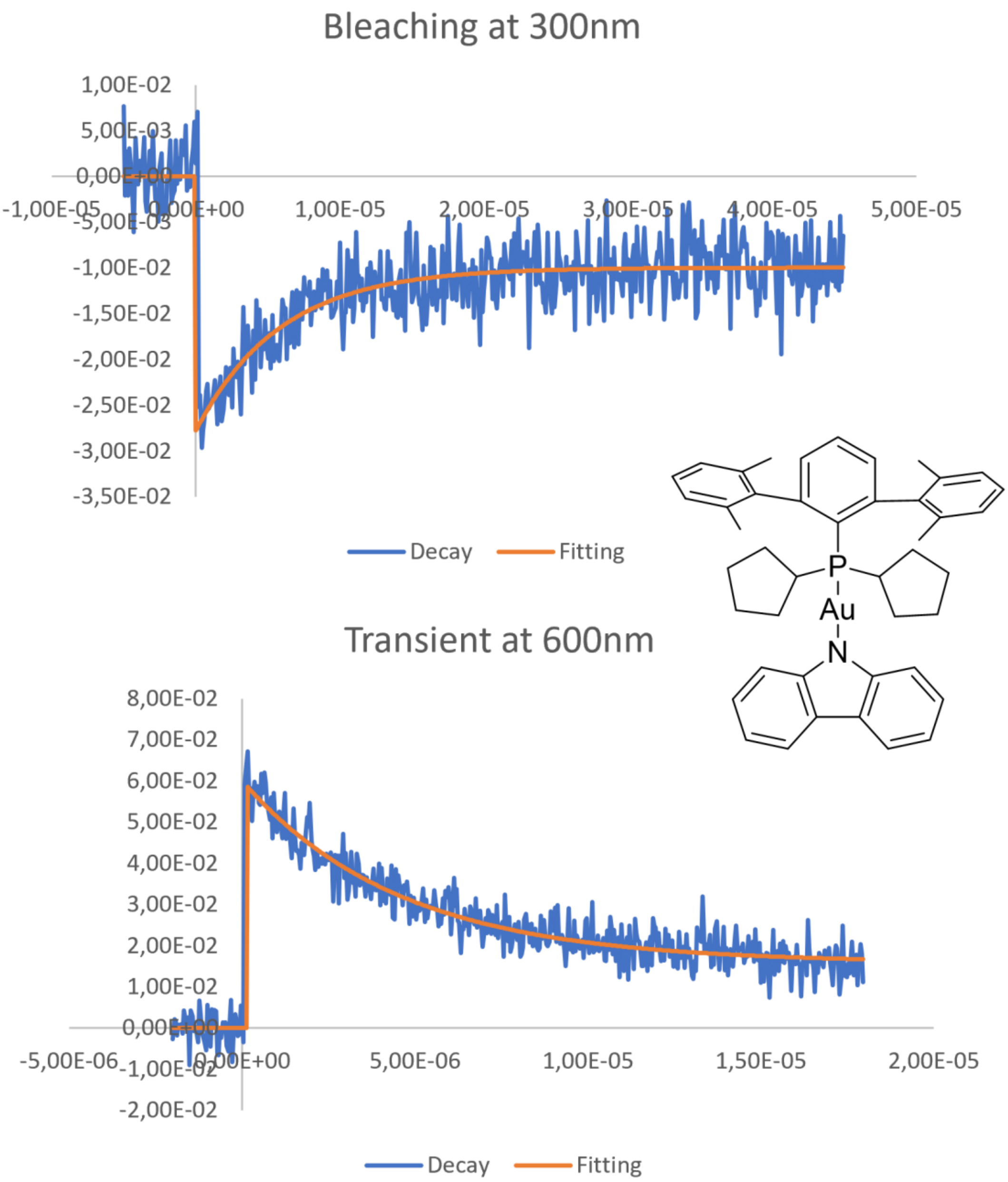

Figure S62. Bleaching and Transient spectra of **2a** in the exclusion of oxygen.

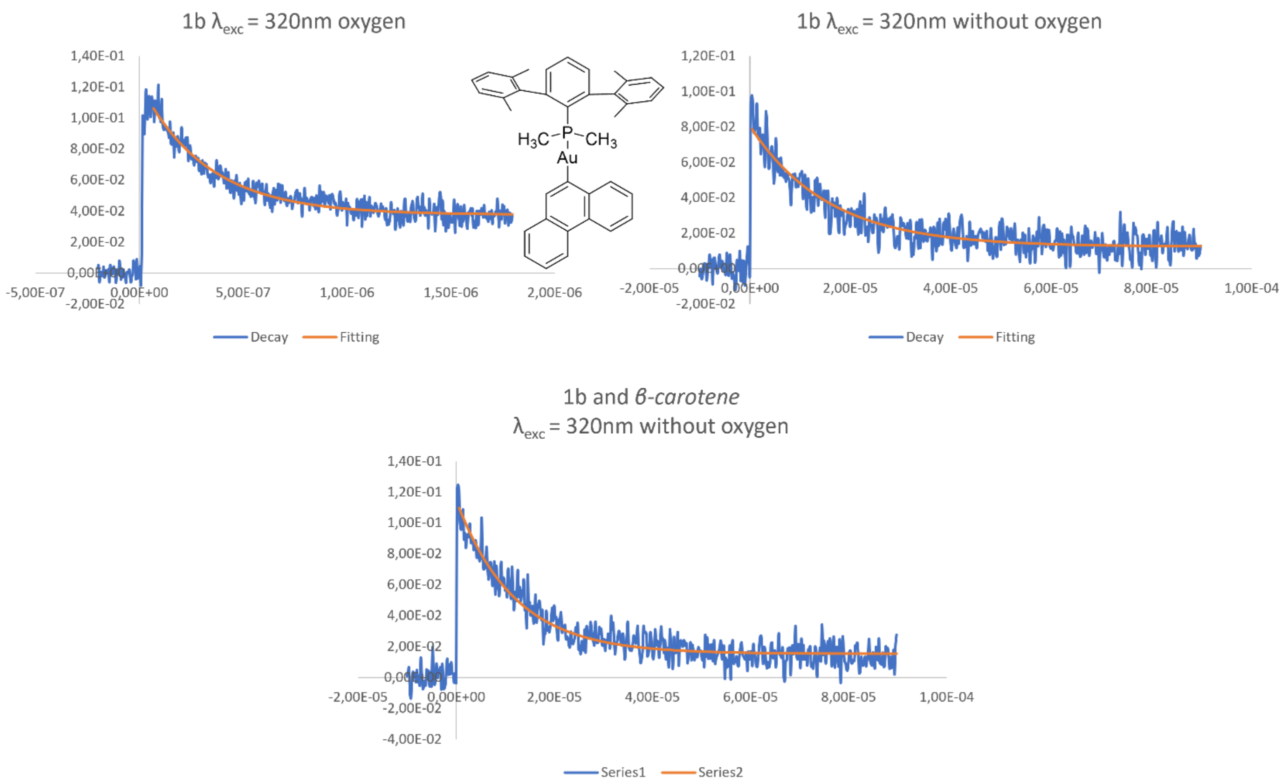

Figure S63. Spectra of **1b** measured with the Energy Transfer method.

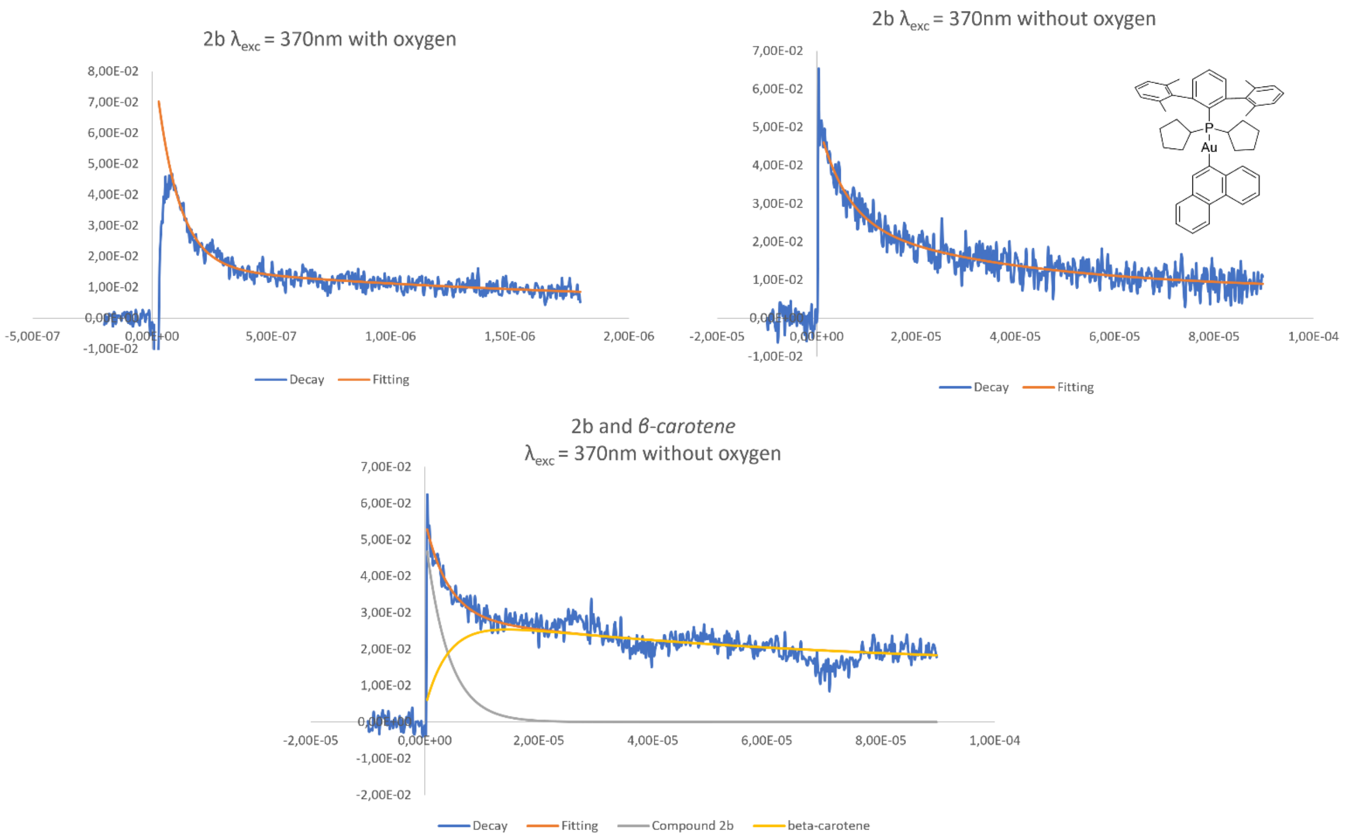

Figure S64. Spectra of **2b** measured with the Energy Transfer method.

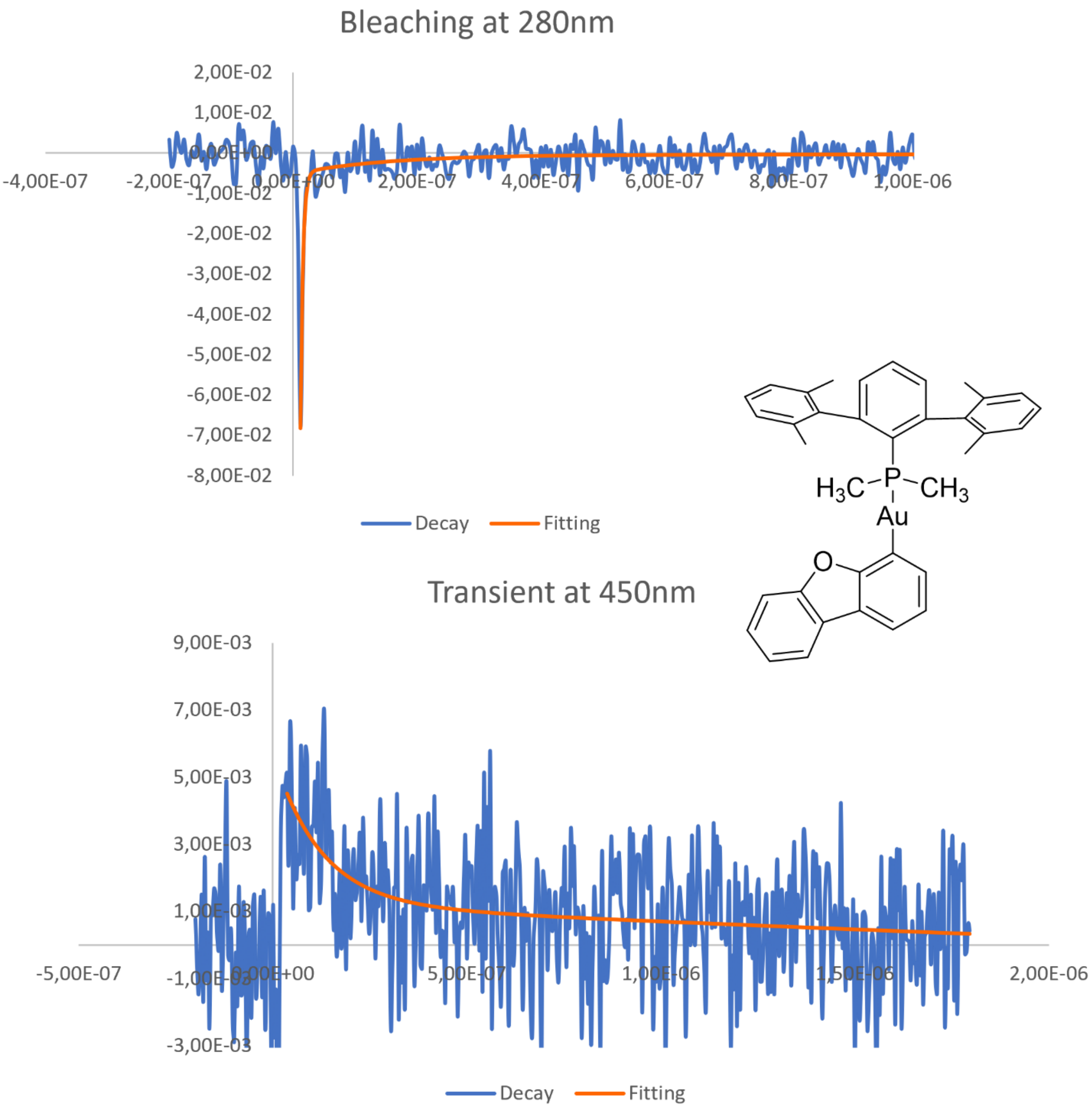

Figure S65. Bleaching and Transient spectra of **1c** in the presence of oxygen.

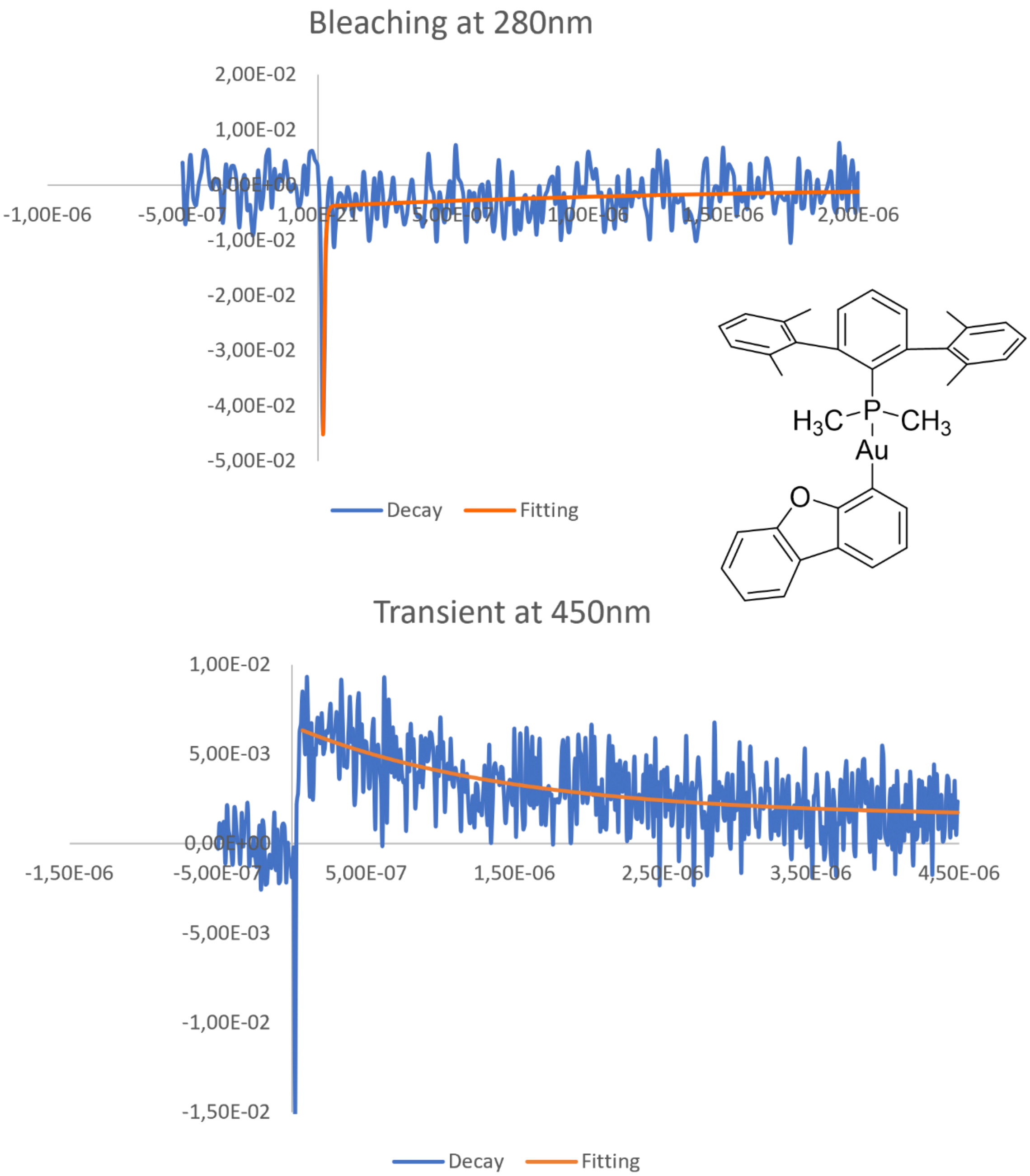

Figure S66. Bleaching and Transient spectra of **1c** in the exclusion of oxygen.

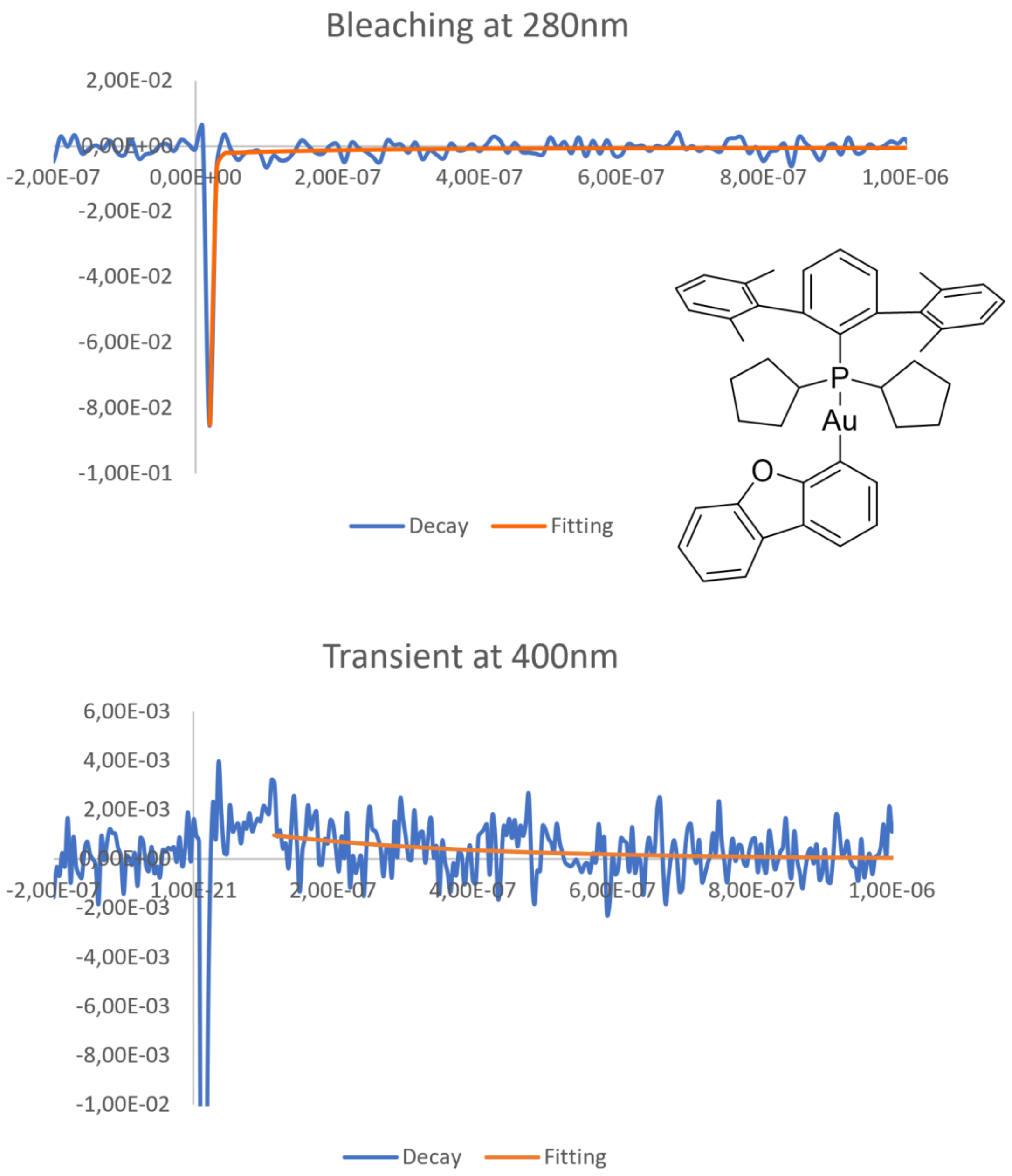

Figure S67. Bleaching and Transient spectra of **2c** in the presence of oxygen.

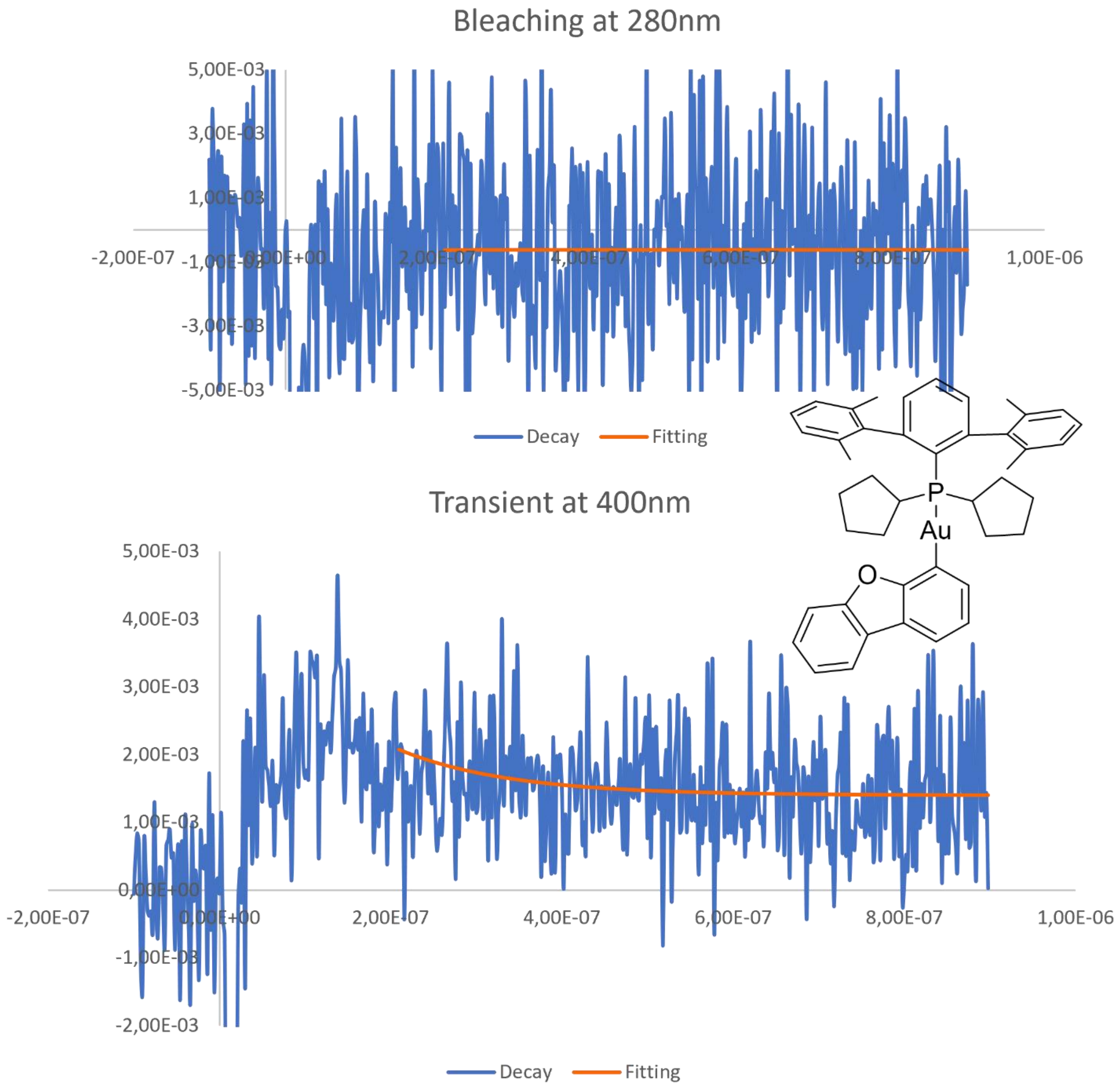

Figure S68. Bleaching and Transient spectra of **2c** in the exclusion of oxygen.

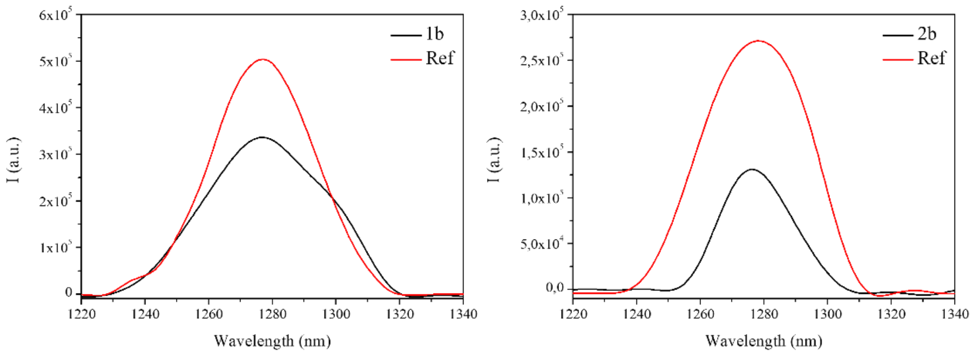

Figure S69. $^1O_2$ production of gold(I) complexes **1b** and **2b** in dichloromethane air-equilibrated solutions at λ_{exc} = 311 nm.

4. Computational study

1a

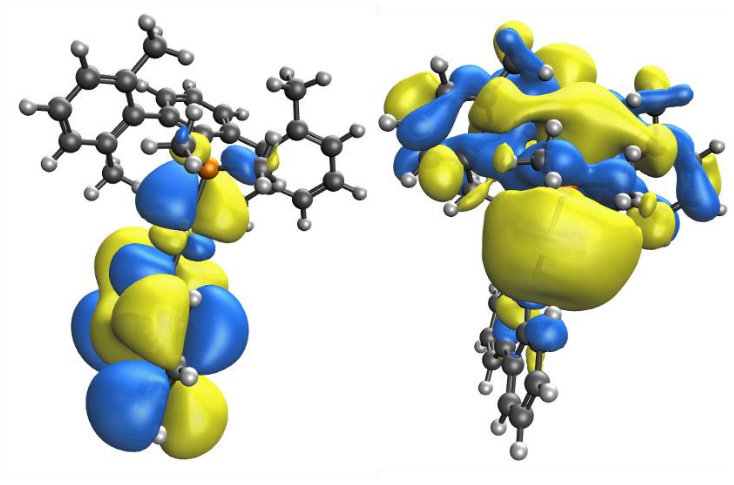

HOMO **LUMO**

Figure S70. HOMO and LUMO orbitals involved in the studied transitions of compound **1a**.

1b

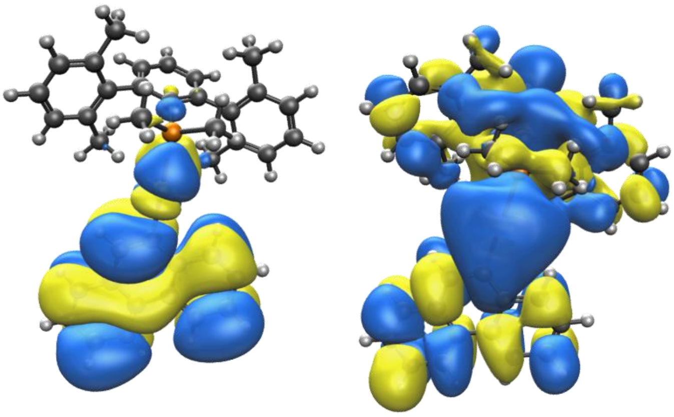

HOMO **LUMO**

Figure S71. HOMO and LUMO orbitals involved in the studied transitions of compound **1b**.

1c

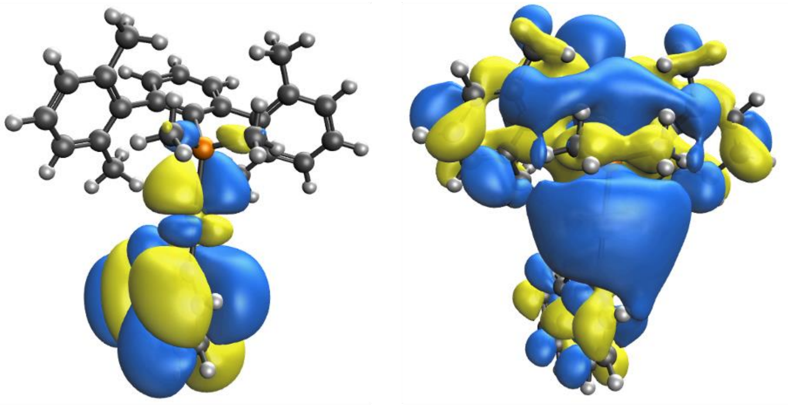

HOMO LUMO

Figure S72. HOMO and LUMO orbitals involved in the studied transitions of compound **1c**.

2a

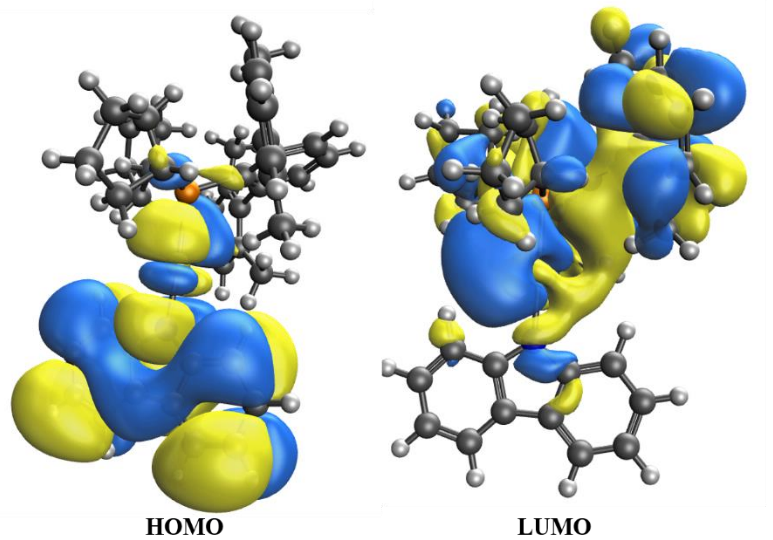

HOMO LUMO

Figure S73. HOMO and LUMO orbitals involved in the studied transitions of compound **2a**.

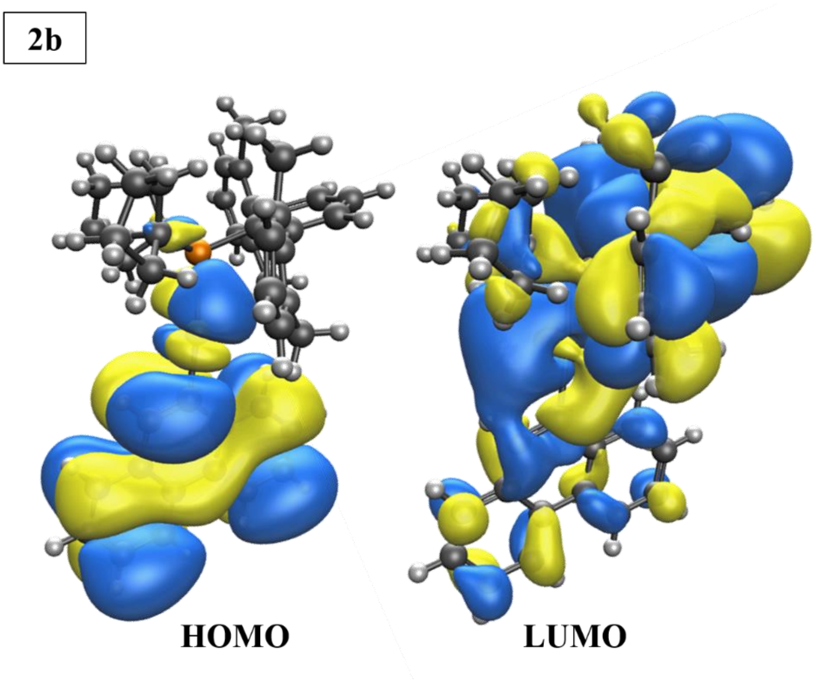

Figure S74. HOMO and LUMO orbitals involved in the studied transitions of compound **2b** and its MEP diagram.

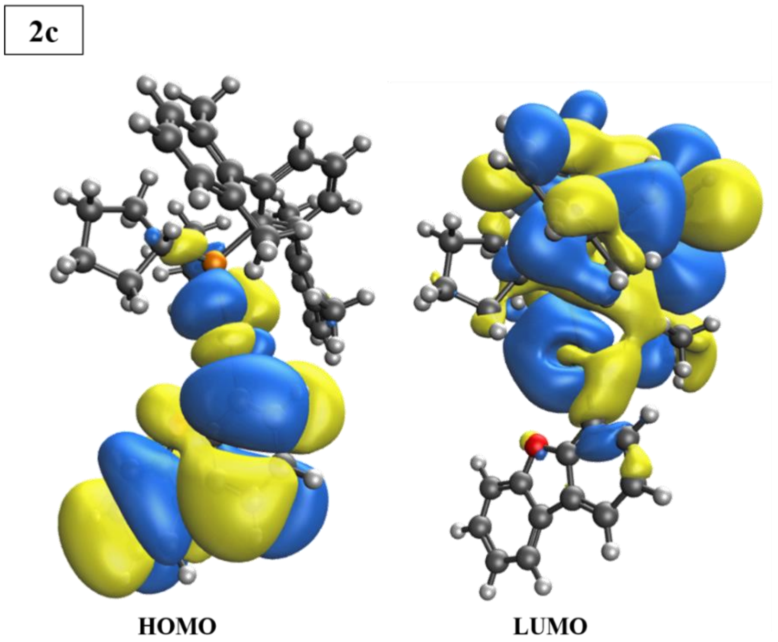

Figure S75. HOMO and LUMO orbitals involved in the studied transitions of compound **2c**.

5. Tables

Table S1. Crystal data and structure refinement for **1a**, **1b**, **2b,** and **2c**.

**Compound**	**1a**	**1b**	**2b**	**2c**
Formula	$C_{36}H_{35}AuNP$	$C_{38}H_{36}AuP$	$C_{46}H_{48}AuP$	$C_{44}H_{46}AuOP$
Crystal size, mm	0.03 x 0.05 x 0.08	0.02 x 0.03 x 0.05	0.02 x 0.03 x 0.05	0.03 x 0.03 x 0.08
Fw	709.58	702.60	828.78	818.74
Temp., K	100(2)	100(2)	100.15	120.01(10)
Wavelenght, Å	0.72932	0.72932	0.72932	0.72932
Crystal system	Monoclinic	Monoclinic	Monoclinic	Monoclinic
Space group	$P2_1/c$	$P2_1/c$	$P2_1/c$	$P2_1/c$
a, Å	13.148(3)	13.367(3)	18.2688(13)	18.2201(15)
b, Å	11.437(2)	11.658(2)	11.3675(8)	11.2865(9)
c, Å	19.754(4)	19.693(4)	18.9461(14)	18.8303(16)
α, °	90	90	90	90
β, °	94.74(3)	103.16(3)	114.632(2)	115.475(2)
γ, °	90	90	90	90
Volume, $Å^3$	2960.3(10)	2988.2(11)	3576.5(4)	3495.8(5)
Z	4	4	4	4
$D_{calc.}$, mg m^{-3}	1.592	1.602	1.539	1.556
Abs. coef., mm^{-1}	5.385	5.335	4.469	4.574
F(000)	1408	1432	1672	1648
θ range for data coll, °	1.595 to 28.672	1.605 to 27.873	1.335 to 27.161	1.335 to 27.287
Refins coll./independent	44533/6697	41434/6189	32365/6763	34977/7118
Data/restraint/parameters	6697/0/358	6189/0/367	6763/7/437	7118/7/428
GOF on F^2	1.051	1.055	1.090	1.096
Final *R* index ($I > 2\sigma(I)$)	R1 = 0.0277, wR2 = 0.0786	R1 = 0.0299, wR2 = 0.0799	R1 = 0.0776, wR2 = 0.1995	R1 = 0.0524, wR2 = 0.1215
R index (all data)	R1 = 0.0284, wR2 = 0.0792	R1 = 0.0305, wR2 = 0.0802	R1 = 0.0816, wR2 = 0.2030	R1 = 0.0602, wR2 = 0.1261
Peak and hole, e $Å^{-3}$	1.93 and -0.96	1.85 and -1.33	10.50 and -2.91	4.23 and -1.22
CCDC	2300097	2300099	2300100	230098

Table S2. Radiative (k_r) and non-radiative (k_{nr}) rate constants calculated for all the non-aerated solutions.

Compound	k_r ($10^6 s^{-1}$)	k_{nr} ($10^6 s^{-1}$)
**1a**	3.4	89.2
**2a**	7.2	75.5
**1b**	4.9	70.8
**2b**	3.4	48.4
**1c**	16.5	110.1
**2c**	10.9	64.3

Table S3. The calculated S_n and T_n energies by TD-DFT for **1a** and **2a**, with their corresponding $S_1 \rightarrow T_n$ transition and its main contribution in %.

Compound	Energy (S_n) (eV)	Transitions	Energy (T_n) (eV)	Transitions
**1a**	S_1: 3.3903<br>f = 0.0000	H → L (98.7%)	**T_3: 3.3665**<br>f = 0.0000	H → L (98.3%)
	S_2: 3.6526<br>f = 0.0244	H-1 → L+9 (2%)<br>H → L+2 (95.8%)	T_2: 3.2168<br>f = 0.0000	H-1 → L+2 (67.3%)<br>H → L+9 (15.8%)<br>H-4 → L+2 (6.5%)
	S_3: 3.8910<br>f = 0.0285	H → L+1 (97.1%)	T_1: 3.2168<br>f = 0.0000	H → L+2 (94.5%)<br>H-4 → L+9 (2.2%)
**2a**	S_1: 3.3805<br>f = 0.0020	H → L (99.0%)	T_3: 3.5186<br>f = 0.0000	H-8 → L+1 (15.5%)<br>H-9 → L (14.4%)<br>H-2 → L+1 (7.9%)
	S_2: 3.6360<br>f = 0.0371	H → L+2 (95.7%)	**T_2: 3.3559**<br>f = 0.0000	H → L (95.6%)
	S_3: 3.8209<br>f = 0.0069	H → L (98.0%)	T_1: 3.2155<br>f = 0.0000	H-1 → L+2 (68.1%)<br>H → L+9 (11.0%)

Table S4. The calculated S_n and T_n energies by TD-DFT for **1b** and **2b**, with their corresponding $S_1 \rightarrow T_n$ transition and its main contribution in %.

Compound	Energy (S_n) (eV)	Transitions	Energy (T_n) (eV)	Transitions
**1b**	S_1: 3.8532<br>f = 0.0192	H → L+1 (71.8%)<br>H-1 → L (10.5%)<br>H-1 → L+2 (9.9%)	T_7: 3.8681<br>f = 0.0000	H → L+8 (19.2%)<br>H → L+1 (14.9%)
	S_2: 3.9669<br>f = 0.2441	H → L (91.8%)<br>H-1 → L+1 (4.4%)	**T_6: 3.6815**<br>f = 0.0000	H-5 → L+5 (19.9%)<br>H-7 → L+4 (13.9%)<br>H-3 → L+7 (13.2%)
	S_3: 4.1865<br>f = 0.0462	H → L+2 (79.1%)<br>H-1 → L+1 (8.4%)	T_5: 3.6705<br>f = 0.0000	H-5 → L+4 (21.5%)<br>H-6 → L+5 (10.8%)
**2b**	S_1: 3.8416<br>f = 0.0217	H → L+2 (61.3%)<br>H → L+1 (18.4%)	T_7: 3.8858<br>f = 0.0000	H → L+8 (30.3%)<br>H-1 → L+1 (11.0%)<br>H-8 → L+1 (10.5%)
	S_2: 4.0538<br>f = 0.2226	H → L (45.4%)<br>H → L+1 (31.1%)<br>H → L+2 (12.8%)	**T_6: 3.6492**<br>f = 0.0000	H-7 → L+4 (10.4%)<br>H-3 → L+7 (10.3%)<br>H-6 → L+6 (8.9%)
	S_3: 4.0756<br>f = 0.0664	H → L (52.5%)<br>H → L+1 (35.2%)	T_5: 3.6379<br>f = 0.0000	H-7 → L+5 (12.6%)<br>H-3 → L+4 (10.9%)<br>H-4 → L+5 (8.9%)

Table S5. The calculated S_n and T_n energies by TD-DFT for **1b** and **2b**, with their corresponding $S_1 \rightarrow T_n$ transition and its main contribution in %.

Compound	Energy (S_n) (eV)	Transitions	Energy (T_n) (eV)	Transitions
**1c**	S_1: 4.3151<br>f = 0.1201	H-1 → L+1 (52.8%)<br>H → L+1 (27.9%)<br>H → L (2.4%)	**T_8: 4.3120**<br>f = 0.0000	H-9 → L (60.7%)<br>H-5 → L (12.8%)<br>H-6 → L+2 (5.7%)
	S_2: 4.4034<br>f = 0.0027	H → L (90.9%)	T_7: 4.2143<br>f = 0.0000	H → L+8 (28.0%)<br>H-8 → L+8 (14.9%)<br>H-1 → L+10 (12.9%)
**2c**	S_1: 4.3069<br>f = 0.1135	H-1 → L+1 (47.2%)<br>H → L+1 (41.2%)	T_{10}: 4.3532<br>f = 0.0000	H-4 → L+4 (19.3%)<br>H-4 → L (16.1%)<br>H-3 → L+3 (10.1%)
	S_2: 4.3977<br>f = 0.0013	H → L (95.1%)<br>H-1 → L (3.1%)	**T_9: 4.3008**<br>f = 0.0000	H-9 → L+2 (22.5%)<br>H-3 → L+2 (10.7%)<br>H-2 → L (10.6%)
	S_3: 4.4720<br>f = 0.4008	H → L+1 (48.6%)<br>H-1 → L+1 (41.1%)	T_8: 4.2205<br>f = 0.0000	H-9 → L (30.3%)<br>H-3 → L (21.9%)<br>H-5 → L (19.3%)

6. Catalyst screening

Table S6. Catalytic activity of gold(I) complexes in the hydroamination of phenylacetylene with aniline.[a]

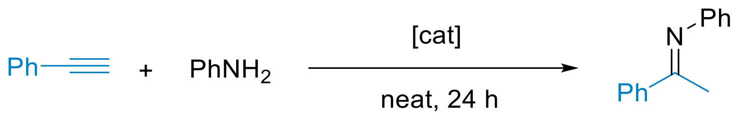

Entry	Catalyst	Au (mol %)	T (°C)	Conversion (%)
1	AuCl**P1**	0.1	50	nr
2	AuCl**P2**	0.1	50	nr
3	**2a**	0.1	50	nr
4	**2b**	0.1	50	nr
5	**2c**	0.1	50	nr

[a] Reaction conditions: phenylacetylene (5 mmol), aniline (5 mmol). Conversions determined by GC using dodecane as internal standard. nr = no reaction

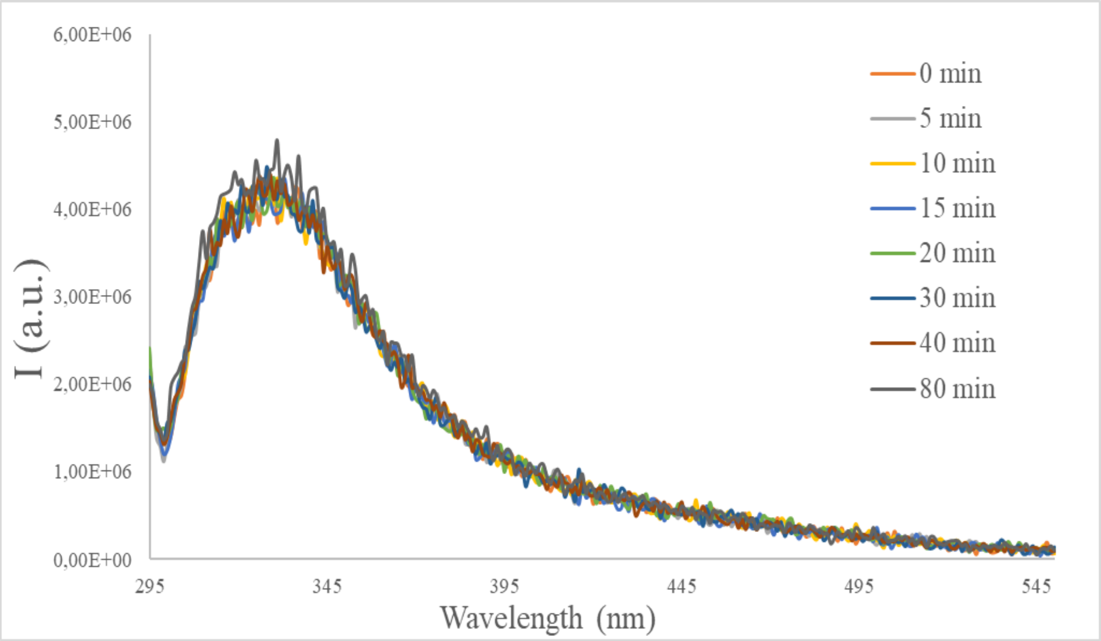

Figure S76. Emission spectra of **AuClP2** along time under the catalytic conditions (in the presence of the reagents).

7. General catalytic procedure for hydroamination reactions.

The catalyst **AuClP2** (0.1 mol%), NaBAr^F (0.1 mol%), the alkyne (2.5 mmol), and the amine (2.5 mmol) were placed into a vial equipped with a J Young tap containing a magnetic bar. The

reaction mixture was stirred at room temperature unless otherwise indicated for 24 h. After this time, the mixture was evaporated to dryness. Products were purified by crystallization or distillation in a Kugelrhor distillation apparatus under a high vacuum (dependent on the boiling point). All the products were described in the literature. However, ^{1}H NMR spectra of these compounds used to identify them have been included herein along with the references where their spectroscopic characterization appeared.

8. Characterization data of reaction products.

(*E*)-*N*-diphenylethan-1-imine (Scheme 3, aa)[1]

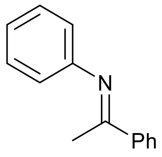

Following the general catalytic procedure, the product was obtained as a yellow solid after recrystallization in pentane. Yield: 468 mg (96%)

^{1}H NMR (300 MHz, $CDCl_3$, 25 ºC): δ 8.0-7.96 (m, 2H,), 7.48-7.43 (m, 3H), 7.38-7.33 (m, 2H), 7.12-7.06 (m, 1H), 6.82-6.79 (m, 2H), 3.0 (s, 3H).

(*E*)-*N*-(4-Methoxyphenyl)-1-phenylethan-1-imine (Scheme 3, ab)[1]

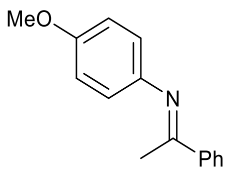

Following the general catalytic procedure, the product was obtained as a yellow solid after re-crystallization in pentane. Yield: 422 mg, (75%).

^{1}H NMR (300 MHz, $CDCl_3$, 25 ºC): δ 7.99-7.95 (m, 2H), 7.47-7.42 (m, 3H), 6.94-6.89 (m, 2H), 6.79-6.74 (M, 2H), 3.82 (s, 3H), 2.26 (s, 3H).

Ethyl (*E*)-4-((1-phenylethylidene)amino)benzoate (Scheme 3, ac)[2]

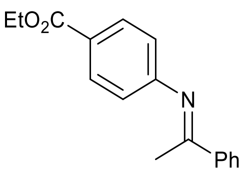

Following the general catalytic procedure, the product was obtained as a yellow solid after recrystallization in pentane. Yield: 600 mg (90%).

^{1}H NMR (300 MHz, $CDCl_3$, 25ºC): δ 8.05 (d, 2H, J = 8.3 Hz), 7.98-7.96 (m, 2H), 7.48-7.45 (m, 3H), 6.83 (d, 2H, J = 8.3 Hz), 4.37 (q, 2H, J = 7.1 Hz), 2.23 (s, 3H), 1.40 (t, 3H, J = 7.1 Hz).

---

[1] M. C. Hansen, S. L. Buchwald, *Org. Lett.* 2000, **2**, 713-715.
[2] Y. Wei, I. Deb, N. Yoshikai, *J. Am. Chem. Soc.* 2014, **136**, 2928-2928.

(*E*)-*N*-(3,5-bis(trifluoromethyl)phenyl)-1-phenylethan-1-imine (Scheme 3, ad)[3]

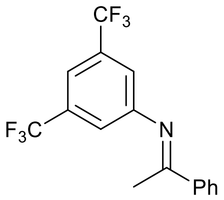

Following the general catalytic procedure, the product was obtained as a yellow solid after recrystallization in pentane. Yield: 760 mg (92%) yield.

^{1}H NMR (300 MHz, $CDCl_3$, 25 ºC): δ 7.99-7.95 (m, 2H), 7.60 (br, 1H), 7.55-7.44 (m, 3H). 7.24 (br, 2H), 2.27 (s, 3H)

(*E*)-*N*-mesityl-1-phenylethan-1-imine (Scheme 3, ae)[4]

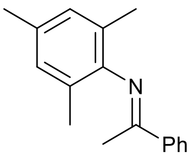

Following the general catalytic procedure, the reaction was carried out at 50 ºC. The product was obtained as a yellow solid after recrystallization in pentane. Yield: 550 mg, (93%).

^{1}H NMR (300 MHz, $CDCl_3$, 25 ºC): δ 8.04-8.01 (m, 2H), 7.49-7.45 (m, 3H), 6.88 (s, 2H), 2.29 (s, 3H), 2.07 (s, 3H), 2.0 (s, 6H).

(*E*)-*N*-(2,6-diisopropylphenyl)-1-phenylethan-1-imine (Scheme 3, af)[5]

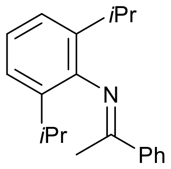

Following the general catalytic procedure, the reaction was carried out at 50 ºC. The product was obtained after recrystallization in pentane as a yellow solid. Yield: 509 mg (73%).

^{1}H NMR (300 MHz, $CDCl_3$, 25 ºC): δ 8.07-8.04 (m, 2H), 7.51-7.48 (m, 3H), 7.17-7.06 (m, 3H), 2.81-2.72 (m, 2H), 2.10 (s, 3H), 1.17-1.13 (m, 12H).

(*E*)-*N*-phenyl-1-(p-tolyl)ethan-1-imine (Scheme 3, ag)[6]

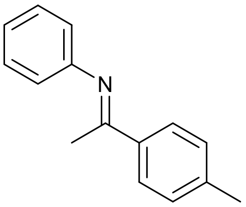

Following the general catalytic procedure, the product was obtained as a yellow solid after recrystallization in pentane. Yield: 380 mg (73%).

^{1}H NMR (300 MHz, $CDCl_3$, 25 ºC): δ 7.89-7.86 (d, 2H, J = 7.86 Hz), 7.35 (t, 2H, J = 7.33 Hz), 7.26 (d, 2H, J = 7.25 Hz), 7.10-7.05 (m, 1H), 6.81-6.78 (m, 2H), 2.42 (s, 3H), 2.22 (s, 3H)

---

[3] T. Imamoto, N. Iwadate, K. Yoshida, *Org. Lett.* 2006, **8**, 2289-2292.
[4] D. Bawari, B. Goswami, S. V. R, S. K. Thakur, R. V. V. Tej, A. R. Choudhury, S. Singh, *Dalton Trans.,* 2018, **47***,* 6274-6278.
[5] S. Katam, P. Ganesan, P. *Dalton Trans,* 2017, **46**, 16615-16622.
[6] W. Lei, G. Hou, M. Chang, X. Zhang, *Adv. Synth. Catal.* 2009, **351**, 3123-3127.

(*E*)-*N*-phenyl-1-(4-(trifluoromethyl)phenyl)ethan-1-imine (Scheme 3, ah)[7]

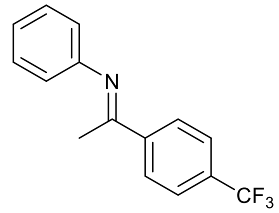

Following the general catalytic procedure, the product was obtained as a yellow solid after recrystallization in pentane. Yield: 414 mg (63%).

^{1}H NMR (300 MHz, $CDCl_3$, 25 ºC): δ 8.07 (d, 2H, *J* = 8.3 Hz), 7.70 (d, 2H, *J* = 8.3 Hz), 7.40-7.34 (m, 2H), 7.14-7.09 (m, 1H), 6.81-6.78 (m, 2H), 2.26 (s, 3H).

***N*-phenylhexan-2-imine (Scheme 3, ai)**[8]

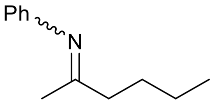

Following the general catalytic procedure, the catalyst **2** (0.2 mol%), NaBAr^F (0.2 mol%), hex-2-yne (2.5 mmol) and aniline (2.5 mmol) were placed into a vial equipped with a J Young tap at 50 ºC for 24 h. The yield and the stereoselectivity were determined by ^{1}H NMR analysis of the reaction crude. Stereoselectivity *E:Z* isomers 3:1. Yield: 71%. Purification was carried out by distillation.

E-imine: ^{1}H NMR (300 MHz, $CDCl_3$, 25 ºC): δ 7.24-7.18 (m, 2H), 6.98-6.93 (m, 1H), 6.67-6.60 (m, 2H), 2.37-2.32 (m, 2H), 1.72 (s, 3H), 1.65-1.50 (m, 2H), 1.45-1.30 (m, 2H), 0.92-0.81 (m, 3H).

Z-imine: ^{1}H NMR (300 MHz, $CDCl_3$, 25 ºC): δ 7.24-7.18 (m, 1H), 7.11-7.06 (m, 2H), 6.73-6.67 (m, 2H), 2.08 (s, 3H), 2.03-1.91 (m, 2H), 1.74-1.69 (m, 2H), 1.30-1.10 (m, 2H), 0.77-0.71 (m, 3H)

1-cyclopropyl-*N*-phenylethan-1-imine (Scheme 3, aj)[9]

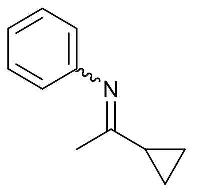

Following the general catalytic procedure, the catalyst **2** (0.2 mol%), NaBAr^F (0.2 mol%), ethynylcyclopropane (2.5 mmol) and aniline (2.5 mmol) were placed into a vial equipped with a J Young tap containing a magnetic bar at 50 ºC for 24 h. The yield and the stereoselectivity were determined by ^{1}H NMR analysis of the reaction crude. Stereoselectivity *E:Z* isomers 3:1. Yield: 92%. Purification was carried out by distillation.

E-imine: ^{1}H NMR (300 MHz, $CDCl_3$, 25 ºC): δ 7.20-7.11 (m, 2H,), 6.90-6.85 (m, 1H), 6.53 (d, *J* = 6.6 Hz, 2H), 1.68-1.60 (m, 1H), 1.60 (s, 3H), 0.88-0.85 (m, 2H), 0.75-0.71 (m, 2H).

E-*Z*-imine: ^{1}H NMR (300 MHz, $CDCl_3$, 25 ºC): δ 7.20-7.11 (m, 2H,), 6.90-6.85 (m, 1H), 6.7 (d, *J* = 6.7 Hz, 2H), 1.68 (s, 3H), 1.52-1.44 (m, 1H), 0.80-0.78 (m, 2H), 0.59-0.56 (m, 2H).

---

[7] W. Lali, F. L. Paglia, W.-F. L. Goff, D. Sredojevic, M. Pfeffer, J. –P. Djukic, *Chem. Commun,* 2012, **48**, 10310-10312.
[8] C. G. Hartung, A. Tillack, H. Trauthwein, M. Beller, *J. Org. Chem*. 2001, **66**, 6339-6343.
[9] J. Kim, D. T. Egger, C. W. Frye, E. P. Beaumier, I. A. Tonks, *Organometallics* 2023, **42**, 1331-1338.

N,1,2-triphenylethan-1-imine and N-(1,2-diphenylvinyl)aniline (Scheme 3 ak)[10]

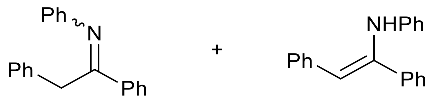

Following the general catalytic procedure, the catalyst **2** (0.2 mol%), NaBAr^F (0.2 mol%), (2.5 mmol) and aniline (2.5 mmol) were placed into a vial equipped with a J Young tap containing a magnetic bar at 80 ºC for 24 h. The yield and the regioselectivity were determined by ^{1}H NMR analysis of the reaction crude. The product was obtained as a mixture of imine:enamine = 3:1. Yield: 71%. Purification was carried out by distillation.

Imine: ^{1}H NMR (300 MHz, $CDCl_3$, 25 ºC): δ 7.87-7.84 (m, 2H), 7.48-7.45 (m, 2H), (all other imine aryl resonances overlap with aryl enamine resonances), 6.78-6.73 (m, 2H), 4.02 (s, 2H).

Enamine: ^{1}H NMR (300 MHz, $CDCl_3$, 25 ºC): δ 6.55-6.52 (m, 2H), (all other aryl enamine resonances overlap with imine aryl resonances), 6.13 (s, 1H), 5, 81 (s, 1H).

Methylphenyl(1-phenylvinyl)amine (Scheme XX, al)[11]

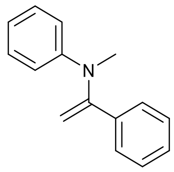

Following the general catalytic procedure, the catalyst **2** (2.5 mol%), NaBAr^F (2.5 mol%), ethynylbenzene (2.5 mmol) and N-methylaniline (2.5 mmol) were placed into a vial equipped with a J Young tap containing a magnetic bar at 80 ºC for 24 h. The yield was determined by ^{1}H NMR analysis. Yield: 79 %. Attempts to purify the compound were unsuccessful.

^{1}H NMR (300 MHz, $CDCl_3$, 25 ºC): δ 7.45-7.42 (m, 2H), 7.27-7.20 (m, 4H), 7.17-7.11 (m, 2H), 6.92-6.89 (m, 2H), 4.97 (s, 1H) 4.79 (s, 1H), 3.23 (s, 3H).

1-(1-phenylvinyl)piperidine and 1-styrylpiperidine (Scheme 3, am)[12]

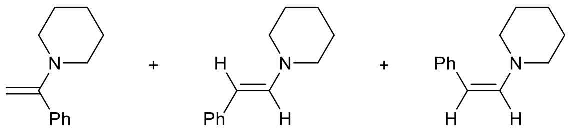

Following the general catalytic procedure, the catalyst **2** (2.5 mol%), NaBAr^F (2.5 mol%), ethynylbenzene (2.5 mmol) and piperidine (2.5 mmol) were placed into a vial equipped with a J Young tap containing a magnetic bar at 80 ºC for 24 h. The yield, regioselectivity and stereoselectivity were determined by ^{1}H NMR analysis of the reaction crude. Product was obtained as a mixture of enamines *gem*:*E*:*Z* = 1:1.4:1.4. Yield: 95%. Purification was carried out by distillation.

[10] L. L. Anderson, J. Arnold, R. G. Bergman, *Org Lett.* 2004, **6,** 2519-2522.
[11] L. Wang, H. Neumann, M. Beller, *Angew. Chem. Int. Ed.* 2019, **58**, 5417–5421
[12] A. Tillack, H. Trauthwein, C. G. Hartung, M. Eichberger, S. Pitter, A. Jansen, M. Beller, *Monatshefte für Chemie*, 2000, **131**, 1327-1334.

E-enamine: ^{1}H NMR (300 MHz, $CDCl_3$, 25 ºC): δ 7.99-7.95 (m, 2H), 7.58-7.03 (m, *trans*-enamine aryl resonances overlap with other enamine resonances), 6.68 (d, 1H, J = 15.5 Hz), 6.33 (d, 1H, J = 15.5 Hz), 2.91-2.90 (m, 2H), 2.82-2.78 (m, 2H), 1.68-1.50 (m, 6H).

Z-enamine: ^{1}H NMR (300 MHz, $CDCl_3$, 25 ºC): δ 7.58-7.03 (m, *cis*-enamine aryl resonances overlap with other enamine resonances), 6.64 (d, 1H, J = 10.8 Hz), 5.60 (d, 1H, J = 10.8 Hz), 2.91-2.90 (m, 2H), 2.82-2.78 (m, 2H), 1.68-1.50 (m, 6H).

gem-enamine: ^{1}H NMR (300 MHz, $CDCl_3$, 25 ºC): δ 7.58-7.03 (m, enamine aryl resonances overlap with other enamine resonances), 4.24 (s, 1H), 4.15 (s, 1H), 2.91-2.90 (m, 2H), 2.82-2.78 (m, 2H), 1.68-1.50 (m, 6H).
